# Higher education institutions and the use of marketing-mix choice architecture strategies to encourage plant-rich menu options and sustainable dietary patterns: a scoping review

**DOI:** 10.3389/fnut.2026.1774451

**Published:** 2026-03-24

**Authors:** Nicole L. Furr, Jessica R. Spence, Eojina Kim, D. Enette Larson-Meyer, Elena Serrano, Vivica I. Kraak

**Affiliations:** 1 Department of Human Nutrition, Foods, and Exercise, Virginia Tech, Blacksburg, VA, United States; 2 Department of Agriculture, Leadership, and Community Education, Virginia Tech, Blacksburg, VA, United States; 3 Howard Feiertag Department of Hospitality and Tourism Management, Pamplin College of Business, Virginia Tech, Blacksburg, VA, United States

**Keywords:** choice architecture, foodservice, higher education, marketing-mix, nudge, plant-rich dietary pattern, sustainable dietary patterns

## Abstract

**Introduction:**

Higher education dining services can leverage their institutional food procurement and marketing influence to accelerate and support eating patterns and food environments aligned with planetary health. Although behavioral economics and nudge-based choice architecture offer low-cost tools to shift demand, sector-wide evidence on how university and college foodservice operations apply these marketing-mix choice architecture strategies beyond the lab and trials in dining settings is limited.

**Methods:**

We conducted a systematic scoping review (2010–2024) across five databases, Google Scholar, gray literature resources, news articles, media releases, and targeted organizational websites to identify United States higher education institutions applying marketing-mix choice architecture strategies to encourage customers to select plant-rich menu options. We categorized evidence describing higher education institutions and their use of behavior change strategies into eight marketing-mix choice architecture strategy domains: place, profile, portion, pricing, promotion, default picks, priming/prompting, and proximity.

**Results:**

The search yielded 363 reports covering 166 higher education institutions located across 36 states and the District of Columbia. Institutions most commonly used profile (96.99%) followed by priming/prompting (45.18%), promotion (39.76%), portion (15.66%), default picks (10.24%), proximity (6.02%), pricing (5.42%), and place (1.20%).

**Discussion:**

Our results contribute to the knowledge to practice gap by revealing how higher education institutions have used behavioral economics strategies to encourage sustainable dietary patterns. This study may provide guidance for university decision-makers, campus dining and foodservice management, and researchers aiming to foster sustainable food environments by documenting the interventions that are commonly applied by higher education institutions at scale beyond the lab setting.

## 1 Introduction

Planetary health, defined as “how well the planet sustains the conditions necessary for life on Earth, including humans” (1) (p. 9), impacts how the planet sustains the conditions necessary for human and ecological health. In 2025, the Planetary Health Check Report described planetary health is threatened due to the risk of exceeding seven out of nine planetary boundaries including: climate change, change in biosphere integrity, land system change, freshwater change, modification of biogeochemical flows, introduction of novel entities, and ocean acidification (1). Further, evidence shows the current food system undermines human and planetary health due to the high demand of land and freshwater use, contribution to greenhouse gas emissions (GHGE), impact on biodiversity loss, and promotion of unhealthy eating patterns (2, 3).

In 2015, the United Nations (UN) Member States adopted the Sustainable Development Goals (SDGs) to build an environmentally sustainable world by 2030; this includes SDG 12, which emphasizes responsible consumption, sustainable production patterns, and the need for individuals to adopt sustainable lifestyles (4). Healthy, sustainable, and plant-rich dietary patterns have lower environmental impacts compared to a Western dietary pattern (5, 6). Therefore, an increase in plant-rich dietary patterns enhances the potential to reach SDG 12 through both sustainable food production and consumption.

Within the United States (US), population dietary patterns align with a Western-style pattern due to the overconsumption of added sugars, saturated fat, and red and processed meats (7, 8). Health and sustainability experts recommend shifts in these widespread dietary patterns. For example, in 2019, the EAT-*Lancet* Commission on Healthy Diets from Sustainable Food Systems recommended a dietary shift from an unhealthy Western diet to the planetary health diet consisting of whole, minimally processed, plant-sourced foods (i.e., whole grains, vegetables, fruits, legumes, and nuts) and to limit the overconsumption of animal-sourced foods (i.e., red meats, processed meats, and dairy products) (3). Not only would these changes improve general health among the US population, but shifts to plant-rich dietary patterns are also needed to meet climate, biodiversity, and sustainability goals.

The US population makes more than 50% of food purchases in food-away-from-home settings, revealing the growing trend of US consumers purchasing meals from foodservice establishments instead of preparing and cooking meals at home (9). The foodservice industry, including campus and dining options, functions as both a service provider and marketing enterprise that can influence consumer demand (10). By implementing sustainable food sourcing, production, and promotional strategies, institutional foodservice managers can drive population transitions to plant-rich dietary patterns and maintain customer satisfaction and operational performance to create healthy and sustainable food environments (10–13).

Institutional food procurement, described as the purchase and preparation of foods and beverages among public and private institutions, could increase the accessibility of nutritious foods and support sustainable food systems (14). Food procurement strategies can influence food environments. Food environments are the settings in which different types of food are made available and accessible to consumers (15). Through their food procurement, establishments, accessibility, and promotion, universities and colleges create institutional food environments that influence the dietary patterns of their students, faculty, and staff (16). There are approximately 6,000 higher education institutions in the US, including both public and private colleges and universities enrolling millions of young adults in 2- and 4-year degree programs annually (17, 18). Incoming college and university students are exposed to a new food environment through campus dining during a critical time of their lives when they are developing and refining preferences, dietary patterns, behaviors, and lifestyles for lifelong health (19). Yet, the dietary intake of young adult college students are sub-optimal and do not align with the Dietary Guidelines for Americans for whole grains, fruits and vegetables, or dairy and exceed recommendations for added sugars (20). Higher education students are influenced by the taste, cost, and availability of foods within the campus food environment demonstrating the influence the foodservice sector has on young adults and their dietary patterns (21).

Scholars describe colleges and university settings as “living learning labs,” but they also operate as large-scale foodservice businesses that test marketing and menu innovations later adopted in other institutional or commercial contexts, which may include the promotion of plant-rich dietary patterns and other sustainable practices (11, 22). The development of higher education sustainability and climate action plans, strategies, and policies are an approach to address planetary health goals and create a more sustainable future (23).

In 2021, approximately 155 US colleges and universities implemented sustainability plans and many participated in the Association for the Advancement of Sustainability in Higher Education's (AASHE) Sustainability Tracking, Assessment & Rating System (STARS) consisting of voluntarily tracking, self-reporting, and sharing sustainability practices (24, 25). The AASHE's STARS framework includes long-term sustainability goals for institutions and enables meaningful comparisons within and among universities and colleges over time due to the common set of metrics. The STARS framework measures sustainability efforts related to academics, engagement, operations, and planning and administration (26). Institutional efforts are tracked and higher education institutions earn a final score, individual report, and rating of reporter, bronze, silver, gold, or platinum across all sustainability efforts. Food and dining initiatives, including dining service procurement, are measured under the operations domain and specific criteria. For instance, the criteria for measuring sustainable dining service procurement is measured based on the percentage of food and beverage spending that meets sustainability criteria (i.e., sustainable or ethically produced food and plant-based foods). Many colleges and universities also voluntarily pledged to decrease the climate impact of foods served on campus through the Coolfood and Forward Food pledge by increasing the proportion of plant-rich menu options across institutional food settings (27, 28).

Evaluations of US higher education sustainability efforts have revealed that sustainability plans often address campus food including: recycling, composting, trayless dining, and local food procurement practices; (29) and outline innovations in procurement, menu planning, and kitchen operations through dining services (11). A global study of 108 universities revealed that approximately 75% of universities implemented a vegan dining program and about half provided informational labeling about low-impact food choices to procure and encourage plant-rich menu options (30).

Contrary to planetary health goals and emerging sustainability plans, higher education institutions often procure, advertise, and serve an abundance of animal-sourced products to customers (31). Further, higher education institutional food environments commonly encourage unhealthy foods, beverages, and meal choices (21, 32–35) due to institutional governance policies, food purchasing practices, exclusive contracts with food and beverage corporations, fast food franchise leasing agreements, and beverage pouring rights contracts (36–39). This system poses issues to student health and planetary health. An evaluation of 19 US universities' food purchases revealed procurement practices did not align with the EAT-*Lancet* planetary health diet and exceeded the targets for beef, poultry, and eggs and did not meet the targets for legumes, nuts, and vegetables (40). However, change in higher education institutional policies can reduce these issues by serving as a model and altering the campus food environment. From a foodservice business perspective, colleges and universities can leverage procurement, menu design, and marketing communication as integrated strategies to nudge consumers toward plant-rich dietary patterns and also provide customers with alternative choices (16, 31, 33). This approach can combine sustainability goals with customer experience management.

Although dietary choices are complex and multifaceted, psychologists conceptualize decision-making as a dual process (41, 42). This dual process being either: (1) food-related decisions that are determined quickly, automatically, and unconscious resulting in a gut reaction; or (2) food-related decisions that are made through effortful, slow, reflective, and conscious decision-making to make rational choices (41–43). Institutional foodservices can both influence customers' decision-making processes (44, 45) and also change the physical environment to nudge people to select sustainable dietary choices (41).

Thaler and Sunstein (43) defined nudging as “any aspect of choice architecture that alters people's behavior in a predictable way without forbidding any options or significantly changing their economic incentive” (p. 8). Alternatively, Hollands et al. (46) described choice architecture as “altering the properties or placement of objects or stimuli within micro-environments with the intention of changing health-related behavior” (p. 3). Specifically within higher education settings, researchers have operationalized nudging strategies for meat reduction into three categories: (1) altering the choice architecture of the food retail environment by modifying the presentation and arrangement of menu items, adding to the existing meal options, or manipulating the layout of the dining area; (2) targeting conscious decision-making processes by utilizing promotional messages or introducing pricing incentives; or (3) multimodal interventions targeting both systems simultaneously (47).

Choice architecture and nudge strategies often use prompts to encourage healthy choices or alter the accessibility, availability, and presentation of menu options without eliminating individual choice or altering economic incentives through pricing strategies (41, 43). Libertarian paternalism and the preservation of individual freedom and choice is central to nudge theory to encourage healthy dietary options instead of removing unhealthy and unsustainable food and beverage options for customers (41). Applying nudges in foodservice settings have been emphasized as a low-cost and easy-to-implement environmental change strategy to promote the selection of healthy food choices (48). Some evidence describes implementing nudge strategies to encourage healthy foods in food retail settings may have favorable or neutral food cost and profitability outcomes. University and college foodservice managers perceive the low cost of nudge implementation to be a facilitator to encourage healthy and sustainable menu options, such as fruits and vegetables (49).

Although altering economic incentives is not a traditional nudge strategy, equitably priced menu options influence university and college students' decision making (21, 50). Guided by behavioral economics and nudge theory, Kraak et al. (51) proposed a marketing-mix choice architecture (MMCA) framework for the foodservice sector that included price among other strategies (i.e., place, profile, portion, promotion, picks, priming/prompting, and proximity) that can evaluate US restaurants and food retailers use of behavioral economics (52–54). MMCA strategies can be used to change the priorities of the food environments and/or food, beverage, and menu items served and sold within the food environment to influence customers' purchasing and consumption behaviors through place, profile, portion, pricing, and promotion strategies (51). Furthermore, MMCA strategies can be made to the placement of food, beverages, and menu items served and sold in the food environment to influence customers' purchasing and consumption behaviors through healthy default picks, priming or prompting, and proximity strategies.

Place strategies can be described as making changes to the internal setting of a food environment to influence the customers' expectations about the ambience or atmosphere, which can highlight healthy food and beverage products (51). Foodservice management may consider reducing excessive stimuli that can influence customers to make impulsive decisions and purchase unhealthy or sustainable foods. Profile strategies consist of changing the nutrient profile, quality, smell, taste, texture, or flavor of menu options to align with nutrition or sustainability guidelines. Portion strategies include foodservice management reducing and/or standardizing the portion size of unhealthy or unsustainable foods to better align with nutrition and sustainability guidelines. Pricing strategies may be applied by setting proportionate pricing for smaller portions, limiting price promotions on unhealthy or unsustainable menu options, or introducing price promotions on healthy and sustainable menu options. Thus, it is recommended that these strategies be combined within a single food environment where people make dietary decisions to influence their health.

Promotion strategies involve foodservice management to adhere to responsible food and beverage marketing practices to promote menu options that meet recommended nutrition and sustainability guidelines (51). Healthy default picks involve foodservice management making the automatic choice the healthy and sustainable choice. Developing healthy default picks may socially normalize the menu options that align with nutrition and sustainability guidelines. Priming or prompting strategies involve foodservice management providing information for customers at the point-of-choice or point-of-purchase. This may include the use of menu labeling, symbol, icons, or contextual information. Proximity strategies involve the foodservice management placing healthy and sustainable menu options at eye level or physically closer to customers to ensure they are visible and easy to select.

The eight MMCA strategies can be adapted and applied to various food environments to meet nutrition and sustainable dining goals. Table 1 summarizes the eight MMCA strategies adapted to higher education institutional food environments with the aim to encourage customers to select plant-rich menu options. Due to the importance of the cost of food among university and college students, the MMCA framework and its inclusion of pricing strategies provides a valuable conceptual framework to apply when assessing higher education institutions and their comprehensive use of behavior change strategies in food environments. Further, the MMCA framework uses a novel approach by combining traditional marketing-mix (i.e., product, place, price, and promotion) and nudge strategies that results in a conceptual framework that can be applied to various food environments to facilitate healthy dietary choices (51).

**Table 1:** Summarized MMCA framework adapted to US higher education food and dining settings to encourage customers to select plant-rich menu options.

**Category**	**Strategy**
Alterations made to the properties of the dining service food environment and/or food or beverage products (i.e., menu items served and sold) to influence students, faculty, and staff's selection and consumption behaviors	**Place:** Changing the internal settings of the food environment using lighting, visual cues, or sensory cues to influence student, faculty, and staff's expectation about the ambience or the atmosphere to highlight plant-rich menu options
	**Profile:** Expanding the availability of plant-rich menu options in the food environment by introducing new options, changing the nutritional profile, quality, smell, taste, texture, and flavor of menu options, or increasing the number or ratio of meals aligning with a plant-rich dietary pattern
	**Portion:** Reducing and/or standardizing the portion of food and beverage items (i.e., animal-sourced foods or meals) within a menu option to align with a plant-rich dietary pattern
	**Pricing:** Using pricing strategies (i.e., proportionate pricing for plant-rich options, limiting price promotions on unhealthy and unsustainable menu options, or price promotions on plant-rich menu options) to increase sales and revenue for products aligning with a plant-rich dietary pattern
	**Promotion:** Using responsible food and beverage marketing practices (i.e., changing the name, appearance, appeal, and attractiveness of menu options or food/beverage products) to promote consumer selection of plant-rich menu options
Adjustments made to the placement of food, beverage, and menu items served and sold at dining service food environment and on campus to influence students, faculty, and staff's selection and consumption behaviors	**Default picks:** Using default menu choices by introducing plant-rich menu options as the default option and animal-sourced options available upon request
	**Priming or prompting:** Using information (i.e., menu labeling, logos, icons, semiotics, or contextual information) to influence customers' knowledge and help students, faculty, and staff to select plant-rich menu options at the point-of-choice or point-of-purchase
	**Proximity:** Placing plant-rich menu options at eye level or physically closer to customers at the point-of-choice and point-of-purchase

MMCA: Marketing-mix choice architecture.

MMCA strategies adapted from Kraak et al. (51).

Empirical evidence and evaluations of MMCA strategies in higher education settings determined environmental change strategies effectively encouraged target populations to select plant-rich menu options. In a French university setting, altering the product profile of the menu options by doubling the availability of vegetarian options increased the number of participants that selected vegetarian meals from 23 to 45% during a 4-week study (55). In a Canadian university setting, combined marketing strategies (i.e., proportion, placement, taste-focused labeling, prompting, social media, and promotional posters) significantly increased plant-rich menu sales (56). In a US higher education setting, altering the proximity by ordering the menu options according to carbon footprint increased the sale of lower impact menu options when placed at the beginning of the menu (57). At two US higher education institutions, making plant-rich meals the default choice at university catering events increased the consumption of plant-sourced foods and decreased meat consumption (58). Similarly, individuals exposed to plant-rich default menu choices in a US dining hall setting were more likely to choose a meat-free menu option (59). This demonstrates that MMCA strategies are promising approaches for higher education institutions to translate and apply in foodservice settings to encourage healthy and sustainable eating patterns for university and college populations. Yet, how these strategies have been applied at scale beyond the lab and trials in dining settings is unclear.

Evidence synthesis reviews evaluating the effectiveness of behavioral economics strategies within food environments recommended promising interventions to be translated into real-world foodservice settings to encourage plant-rich dietary patterns (47, 60–62). Yet, findings are often limited to published research and current evidence reviews have not captured gray literature and unpublished university and college commitments, policies, programs, and practices that describe whether and how MMCA strategies have been adopted in real-world dining settings.

Research that relies exclusively on peer-reviewed publications may exclude university and college real-world application of MMCA strategies that are not described or disseminated through scientific literature and there is a need for research exploring novel and underutilized research methods with real-world evidence. Emerging research has identified how universities and colleges in various countries are addressing sustainable dining (30, 63, 64). Most of the US population follow unsustainable and unhealthy dietary patterns (7, 8). Due to the potential impact university and college foodservice decisions have on students' accessibility and availability of sustainable foods, (31) it is important and timely to explore how US higher education institutions are promoting personal and planetary health by alerting the food environment. Notably, the Humane World for Animals published the 2025 College and University Protein Sustainability Scorecard that described the results of a survey of US higher education institutions to create sustainable dining operations on campus by increasing plant-based meals and reducing animal-sourced proteins (65). However, the extent to which and the range of how higher education institutions are applying behavior change theory and using comprehensive marketing strategies in real-world settings is unknown. This represents a gap in the current literature to understand how choice architecture and nudge strategies are being translated into university and college foodservice settings to encourage healthy and sustainable meal selection.

The purpose of this study is to contribute to the theory-practice gap by providing an evidence review and synthesis of how behavioral economics theory and MMCA strategies are being translated into practice across US colleges and universities. These results will inform higher education institutional policies and practices, foodservice operations, and marketing communication to foster sustainable food environments. This research will contribute to the growing body of literature that describes the application of behavior science and sustainability initiatives in higher education settings. Thus, this study will provide practical and feasible strategies to guide foodservice management and operations to implement marketing, choice architecture, and nudge strategies into their dining practices, programs, and policies to promote shifts to healthy, sustainable, and plant-rich dietary patterns.

## 2 Methods

We applied a systematic scoping review guided by two research questions:

Research question 1: Which US higher education institutions used MMCA strategies to encourage customers to select plant-rich menu options that support plant-rich dietary patterns for higher education populations?

Research question 2: To what extent have US higher education institutions used MMCA strategies to encourage customers to select plant-rich menu options?

To address research question one, we conducted a systematic scoping review to identify peer-reviewed, gray literature, and media evidence describing higher education institutional strategies to encourage customers to select plant-rich menu options. Scoping review methodology is used to explore key concepts related to a research area, disseminate findings to policymakers, practitioners, and consumers, and identify research gaps in the existing literature (66). Thus, the exploratory nature of our research questions aligned with the purpose of scoping review methodology to explore the extent, range, or nature of the evidence on a topic (67). The researchers used the Preferred Reporting Items for Systematic Reviews and Meta-Analyses for Extension Scoping Reviews (PRISMA-ScR) checklist (67) (Supplementary File 1) and five steps outlined by Arksey and O'Malley (66): (1) identify the research question; (2) identify relevant literature; (3) select the literature; (4) chart the data; and (5) collate, summarize, and report the results.

Components of a plant-rich dietary pattern can vary and range from the exclusion of animal products (i.e., vegan or vegetarian) to dietary patterns that include limited amounts of meat, fish, eggs, and dairy (i.e., flexitarian) (68, 69). There is a wide range of terms and definitions referring to dietary patterns high in plant foods in scientific literature and media (68). We defined plant-rich menu options as items consisting of plant foods with little or no animal-sourced products (70, 71). Specifically, our search strategy could identify interventions that could increase the consumption of plant-source foods and/or decrease the consumption of animal-source foods. Moreover, we also included terms that foodservice and dining experts frequently use, such as plant-based and plant-forward. Plant-based dietary patterns can be described as excluding animal products, such as following a vegan or vegetarian dietary pattern, or including some meat, fish, eggs, and dairy, such as a flexitarian dietary pattern (68, 69). Plant-forward dietary patterns consists of a variety of plant foods without excluding or eliminating food groups (72).

Table 1 summarizes the eight MMCA strategies to encourage customers to select plant-rich menu options in US higher education settings. The detailed version of the adapted MMCA framework is provided in Table 1 in Supplementary File 2. The lead investigator (NLF) worked with research librarians to design and implement a comprehensive search strategy for the scoping review across five electronic databases [i.e., PubMed, Education Resources Information Center (ERIC), Business Source Complete, ProQuest One Business (ABI/INFORM), and Core Collection], Google Scholar, and NewsBank from January 1, 2010, through December 31, 2024. We selected the year 2010 to capture recent efforts due to the emergence of university and college sustainability initiatives and ranking systems during this time (24, 25).

The researchers constructed the search strategy based on three concepts: university or college campuses (population); plant-rich foods (concept); and MMCA strategies (context). Table 2 summarizes the search strategy for this systematic scoping review. Table 2 in Supplementary File 2 outlines the full search strategy for this systematic scoping review. NLF conducted the final searches on November 15, 2024, and follow-up searches on December 31, 2024, to identify records published after the initial search. NLF conducted the Google Scholar search from January 1, 2010, through December 31, 2024.

**Table 2:** Summary of inclusion and exclusion criteria and search strategy for scoping review.

**Inclusion criteria**	**Exclusion criteria**
1. Peer-reviewed journal articles, gray literature, media stories, news releases, news articles, or US higher education institution website that reports commitments, policies, programs, or practices adopted by a traditional tertiary education setting (i.e., US higher education dining services) to encourage plant-rich menu options through one or more MMCA strategy. 2. Records or reports were published as peer-reviewed articles, book chapters, gray literature reports, or websites between January 1, 2010 and December 31, 2024. 3. Records or reports described commitments, policies, programs, or practices adopted by US higher education dining services. 4. Records or reports with full-text English language version available.	1. Peer-reviewed journal articles, gray literature reports, media stories/news releases, or higher education websites that describe findings from a one-time experimental study or program or annual events that occurred once a year. 2. Only one food, menu item, or recipe change. 3. Records or reports describe interventions outside of the scope of MMCA. 4. Records or reports describe higher education dining services commitments, policies, programs, or practices promoting aspects of sustainable diets or sustainability not related to plant-rich dietary patterns. 5. Records or reports that mention providing plant-rich menu options but there lacks a clear MMCA promotion or strategy. 6. Records or reports take place outside of tradition university or college setting. 7. Records or reports were published before January 1, 2010. 8. Records or reports that describe commitments, policies, programs, or practices adopted by higher education dining services outside of the US. 9. Records or reports that describe a foodservice company commitment to increase plant-rich menu options without evidence that a higher education institution has adopted it in a dining services setting. 10. Full-text English language version of the evidence source was not available. 11. Full books describing MMCA strategies tested in college settings. 12. Systematic reviews or literature reviews describing MMCA strategies tested in university and college settings. 13. Theses, dissertations, patents, conference, or poster abstracts.
**Peer-reviewed literature search strategy**	
**Five electronic databases** 1. PubMed 2. Education Resources Information Center (ERIC) 3. Business source complete 4. ProQuest One Business (ABI/Inform) 5. Core collection	**Search terms** **Concept 1: University/college campuses** ***(Population)*****:** (university OR universities OR undergrad OR undergrads OR undergraduate OR undergraduates OR college OR colleges OR collegiate OR “higher education” OR campus^*^ OR “post-secondary” OR postsecondary OR “post secondary” OR “Post-graduate” OR “Post-graduates” OR “post graduate” OR “post graduates” OR “tertiary education”) AND **Concept 2: Plant-rich foods** ***(Concept)*****:** ((food^*^ OR cater^*^ OR eatery OR eateries OR nutrition OR diet^*^ OR meal^*^ OR dining OR canteen^*^ OR menu^*^ OR cafe^*^ OR lunch^*^ OR breakfast^*^ OR dinner^*^ OR “snack bar^*^” OR grill^*^ OR restaurant^*^) AND (“plant-rich” OR “plant rich” OR “plant-based” OR “plant based” OR “plant alternative^*^” OR “plant-forward” OR “plant forward” OR “plant protein^*^” OR “plant derived” OR “non-animal protein^*^” OR vegetable^*^ OR fruit^*^ OR grain^*^ OR soy OR nut OR nuts OR seed^*^ OR tofu OR tempeh OR bean^*^ OR legume^*^ OR vegan^*^ OR vegetarian^*^ OR flexitarian^*^ OR “meat-free” OR “meat free” OR “alternative protein^*^” OR meatless OR “meat reduction” OR “reduce meat^*^” OR “reduced meat^*^”)) AND **Concept 3: MMCA strategies** ***(Context)*****:** (policy OR policies OR guideline^*^ OR program^*^ OR commitment^*^ OR initiative^*^ OR campaign^*^ OR standard^*^ OR recommendation^*^ OR intervention^*^ OR nudg^*^ OR “choice architecture^*^” OR “behavioral economics” OR “default choice^*^” OR “nutrition profile^*^” OR “nutrient profile^*^” OR “nutritional profile^*^” OR portion^*^ OR promot^*^ OR proximity OR prompting OR pric^*^ OR cost^*^ OR placement OR label^*^ OR “point of purchase” OR “point-of-purchase” OR marketing OR “marketing-mix” OR advertis^*^)
Two gray literature databases	
1. Google Scholar (first 300 search hits)	
Search string: (university AND dining) AND (food OR nutrition OR catering OR “plant-rich” OR “plant-forward” OR “plant-based” OR vegetarian OR vegan OR “meat-free” OR meatless OR commitment OR policy OR program OR practices OR marketing OR nudge OR “choice architecture”)	
2. Access World News (NewsBank; first 300 search hits + follow-up search: first 100 search hits)	
Search string: (university OR universities OR college OR colleges OR campus) AND (“dining services” OR food OR nutrition OR catering OR “plant-rich” OR “plant-forward” OR “plant-based” OR vegetarian OR vegan OR “meat-free” OR meatless OR commitment OR policy OR program OR practices OR marketing OR nudge OR “choice architecture”)	
**Supplemental search strategy**	
**Supplemental search of targeted higher education sustainability ranking systems, commitments, or programs related to plant-rich menu options**	
1. Humane World for Animals	
2. The Coolfood Pledge	
3. Forward Food Pledge	
4. The Menus of Change University Research Collaborative	
5. Meatless Monday website and corresponding resources	
6. AASHE STARS report among previously identified higher education institutions	
**Supplemental search of targeted higher education websites** Supplemental search of targeted higher education websites was conducted by the lead researcher, NLF, after the report was included to identify supplemental information to support the MMCA strategy or other promotional efforts of the previously identified higher education institution.	**Google search string** “higher education name” AND “sustainable OR dining” **Google search string** “higher education name” AND “program, policy, or commitment name” if applicable

MMCA: Marketing-mix choice architecture.

NLF conducted the literature search, compiled the evidence using Covidence (73), and independently screened the titles and abstracts for relevance. During the title and abstract screening, NLF excluded the evidence sources if they did not clearly describe a MMCA strategy, described a sustainability effort that was not related to plant-rich dietary patterns, referenced an initiative outside of the US, or clearly took place outside of a higher education setting. For media and news articles published without an abstract, NLF reviewed the full article prior to including or excluding it from the full-text review.

Two co-investigators (NLF and JRS) screened the full texts using the inclusion and exclusion criteria summarized in Table 2 in Supplementary File 2. The objective of a scoping review is to map the key concepts underpinning a research area and the main sources and types of evidence available (66). Therefore, we decided *a priori* to include any traditional tertiary higher education settings in the US, including colleges and universities. Although it was expected residential institutions would be represented across the evidence sources more frequently than non-residential institutions, all evidence that described the use of MMCA strategies in a university or campus food environment was included in the review. Evidence describing findings from a one-time research study, program, or event were not included in this review due to the research objective to document university and college long-term and real-world sustainability initiatives. The co-investigators first reviewed a subset of the references (*n* = 85), met to discuss alignment with the research questions, and updated the inclusion and exclusion criteria before reviewing all the references.

To address research question two, we categorized the evidence sources using a deductive approach (74). We used a deductive approach guided by the adapted MMCA conceptual framework (Table 1 in Supplementary File 2) to build upon previous literature that has applied the MMCA framework to guide behavior change strategies. We applied the MMCA framework to university and college dining settings due to its inclusion of pricing strategies and novel combination of nudge and marketing-mix principles (51). The deductive analysis and extraction approach was guided by Pollock et al. (74) and consisted of extracting relevant information from data evidence sources and organizing the information according to the establish MMCA framework. NLF and JRS met to resolve any discrepancies in the interpretation of the results.

Due to the nature of behavior change interventions and strategies, it is possible that higher education institutions may have applied multiple MMCA principles into one strategy. If the co-investigators determined the evidence described a behavior change strategy that was not mutually exclusive to only one MMCA strategy, it was included in multiple MMCA category domains simultaneously. NLF iteratively reviewed the organized data and ensured the evidence appropriately described the adapted MMCA framework and corresponding strategies (74).

NLF applied supplemental targeted searches following the initial MMCA categorization to support data triangulation. First, NLF searched the websites of five organizations that described university and college sustainability efforts related to providing plant-rich menu options on higher education campuses including the: (1) Humane World for Animals; (75) (2) Coolfood Pledge participant website; (27) (3) Forward Food Pledge participant website; (28) (4) Menus of Change University Research Collaborative (MCURC) (76) and the Operational Research Publication website and corresponding Plant-Forward Diet Promotion records; (77) and (5) Meatless Monday website and corresponding resources filtered by case studies and success stories (78).

Next, NLF conducted searches of the nutrition and dining websites of the previously identified universities and colleges. Iterative Google searches were conducted March 21, 2025, through August 20, 2025. Lastly, NLF reviewed the current AASHE STARS reports “Food & Dining section” for the previously identified universities and colleges that were active participants in the ranking system during the time of the study. NLF identified and reviewed the AASHE STARS reports via the STARS website between June 17, 2025, and August 13, 2025. Additional sources supporting the MMCA strategy or identifying additional MMCA strategies were organized in a citation manager and included in the final analysis. NLF independently categorized the additional reports according to the MMCA framework.

Next, NLF categorized the identified higher education institutions by geographic region, location, institutional size, and campus setting. The location (i.e., city and state), institutional size, and campus settings were categorized using the Carnegie Classification of Institutions of Higher Education, which is a leading framework for recognizing and describing institutional diversity in US higher education settings (79) and corresponding data set based on the 2025 institutional classification (80). NLF categorized the higher education institutions by geographic region using the Census Regions and Divisions of the US that includes the West, Midwest, Northeast, and South regions (81). The results and common applications of the MMCA strategies are presented as a narrative summary.

## 3 Results

**Figure 1:**
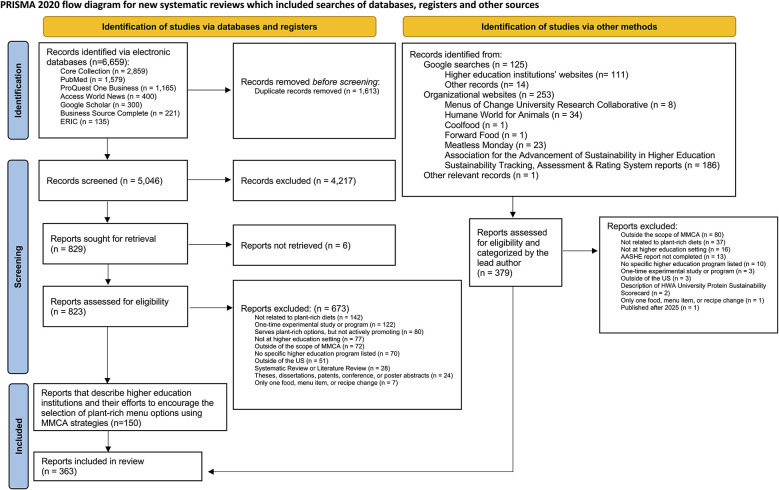
PRISMA flow diagram for systematic scoping review to identify US higher education institutions using MMCA strategies. Source: Page MJ, et al. BMJ 2021; 372:n71. doi: 10.1136/bmj.n71. This work is licensed under CC BY 4.0. To view a copy of this license, visit https://creativecommons.org/licenses/by/4.0/. MMCA: Marketing-mix choice architecture.

Figure 1 presents the PRISMA flow diagram for the systematic scoping review. Tables 3–5 in Supplementary File 2 describe the final search results across the database and supplemental searches. This systematic scoping review and interdisciplinary database search identified 6,659 records that were screened by title and abstract, of which 823 full-text reports were assessed for eligibility. A total of 150 reports met the inclusion criteria describing one or more US higher education institutions and their use of one or more MMCA strategies to encourage customers to select plant-rich menu options. Organizational website searches describing US higher education institutional efforts and iterative Google searches identified an additional 213 reports, such as higher education websites, organizational reports, and additional news articles. Across the electronic databases, Google scholar, and supplemental searches, a total of 363 unique reports that described 166 unique higher education institutions that applied one or more MMCA strategies to encourage their customers to select plant-rich menu options.

**Figure 2:**
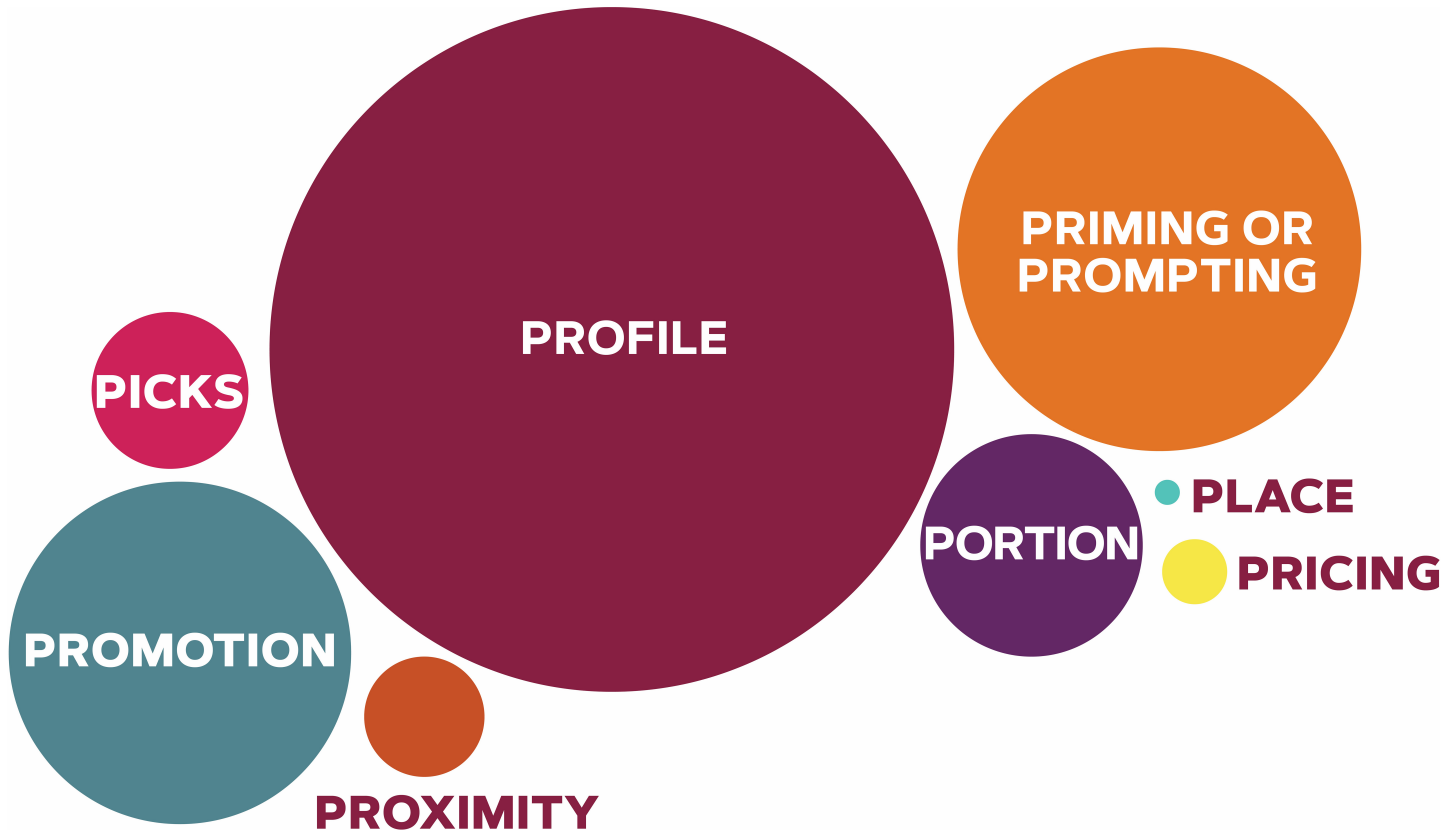
Proportion of higher education institutions and their use of MMCA strategies to encourage customers to select plant-rich menu options. MMCA: Marketing-mix choice architecture. Higher education institutions most commonly used profile (*n* = 161; 96.99%) followed by priming/prompting (*n* = 75; 45.18%), promotion (*n* = 66; 39.76%), portion (*n* = 26; 15.66%), default picks (*n* = 17; 10.24%), proximity (*n* = 10; 6.02%), pricing (*n* = 9; 5.42%), and place (*n* = 2; 1.20%).

The systematic scoping review identified 166 US higher education institutions that used MMCA strategies to encourage customers to select plant-rich menu options between 2010 and 2024 (Table 2). Figure 2 describes the proportion of MMCA strategies that US higher education institutions applied to encourage plant-rich dietary patterns and menu selection. Among the 166 higher education institutions, 2 (1.2%) are categorized as very small (i.e., fewer than 500 students), 32 (19.3%) are categorized as small (i.e., between 500 and 4,000 students), 54 (32.5%) are categorized as medium (i.e., between 4,000 and 20,000 students), 45 (27.1%) are categorized as large (i.e., between 20,000 and 40,000 students), and 32 (19.3%) are categorized as very large (i.e., at least 40,000 students) (80, 82). Of the 166 higher education institutions reviewed, 78 (47.0%) are highly residential, 55 (33.1%) are primarily residential, 26 (15.7%) are residential, 3 (1.8%) are online and on-campus learning, 1 (0.6%) is graduate-focused, 1 (0.6%) is mostly full-time non-residential, and 1 (0.6%) is primarily non-residential (80). One higher education institutions was not included in the Carnegie Classification due to its closure in 2021 (80, 82, 83).

**Figure 3:**
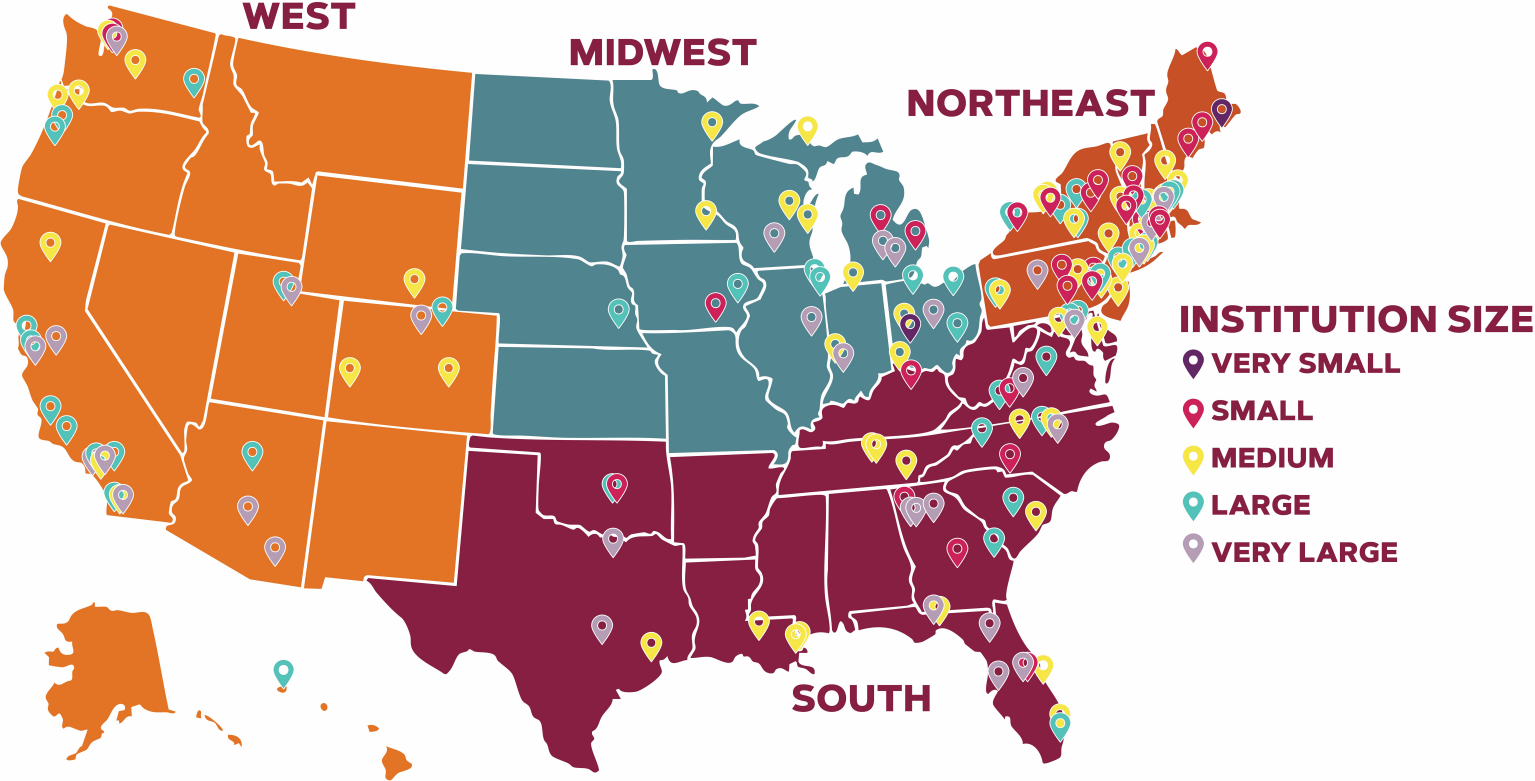
Higher education institutions that used MMCA strategies to encourage customers to select plant-rich menu options across US states and regions. MMCA: Marketing-mix choice architecture. This systematic scoping review identified 166 US higher education institutions that used MMCA strategies to encourage customers to select plant-rich menu options between 2010 and 2024.

We identified higher education institutions across 36 US states and the District of Columbia. Among the 166 higher education institutions, 60 (36.1%) are in the Northeast region, 25 (15.1%) are in the Midwest region, 45 (27.1%) are in the South region, and 36 (21.7%) are in the West region (81). Table 3 summarizes the higher education institutions and their use of MMCA strategies categorized by place, profile, portion, pricing, promotion, picks, priming/prompting, and proximity. Table 4 summarizes the use of MMCA strategies across the identified 166 higher education institutions. Figure 3 outlines the higher education institutions across the US and describes the institution by size. Supplementary File 3 includes Tables 1–8 that outlines the 166 higher education institutions and their applied MMCA strategies categorized by place, profile, portion, pricing, promotion, picks, priming or prompting, and proximity. Supplementary File 4 outlines the identified higher education institutions and their corresponding region, state, institutional size, and MMCA strategies applied.

**Table 3:** Summary of higher education institutions and their use of MMCA strategies categorized by place, profile, portion, pricing, promotion, picks, priming/prompting, and proximity.

**MMCA strategy**	**US higher education institutions that applied MMCA strategy to encourage the selection of plant-rich menu options between 2010–2024**
Place	*n* = 2; 1.20%
Profile	*n* = 161; 96.99%
Portion	*n* = 26; 15.66%
Pricing	*n* = 9; 5.42%
Promotion	*n* = 66; 39.76%
Picks	*n* = 17; 10.24%
Priming or prompting	*n* = 75; 45.18%
Proximity	*n* = 10; 6.02%

MMCA: Marketing-mix choice architecture.

**Table 4:** Summary of the use of MMCA strategies across 166 higher education institutions.

**Higher education institution (State)**	**MMCA strategies**
Alma College (Michigan)	Profile (28), proximity (185)
American University (Washington, D.C.)	Profile (105), promotion (105)
Antioch College (Ohio)	Profile (28)
Appalachian State University (North Carolina)	Profile (186–189)
Arizona State University (Arizona)	Profile (105, 190), priming/prompting (190)
Bastyr University (Washington)	Profile (191)
Belmont University (Tennessee)	Profile (28)
Benedict College (South Carolina)	Profile (28, 192–194)
Bennington College (Vermont)	Profile (195)
Bentley University (Massachusetts)	Profile (28, 196–198), priming/prompting (196)
Berry College (Georgia)	Profile (199)
Binghamton University (New York)	Profile (200–202), promotion (200, 203), priming/prompting (203, 204)
Boston College (Massachusetts)	Profile (205, 206)
Boston University (Massachusetts)	Profile (207–210), promotion (207), priming/prompting (207–209)
Bowdoin College (Maine)	Profile (211–213), priming/prompting (211)
Bowling Green State University (Ohio)	Profile (214, 215)
Brandeis University (Massachusetts)	Profile (27, 216), priming/prompting (216, 217)
Brigham Young University (Utah)	Profile (65, 218), promotion (65), Picks (65), priming/prompting (65)
Bryn Mawr College (Pennsylvania)	Profile (219, 220), priming/prompting (221)
Bucknell University (Pennsylvania)	Profile (222, 223), promotion (223)
California Polytechnic State University (Cal Poly) (California)	Profile (224, 225)
California State University, Chico (Chico State) (California)	Profile (176)
Canisius University (New York)	Profile (105), pricing (105)
Carnegie Mellon University (Pennsylvania)	Profile (105, 226, 227), priming/prompting (226)
Central Washington University (Washington)	Profile (228, 229), portion (230), promotion (228), priming/prompting (229, 231)
Clark University (Massachusetts)	Profile (232), promotion (232)
Colby College (Maine)	Profile (191, 213)
Colgate University (New York)	Profile (233–236), promotion (236, 237)
College of Charleston (South Carolina)	Profile (238, 239), promotion (238), priming/prompting (240)
College of the Atlantic (Maine)	Profile (213)
College of the Holy Cross (Massachusetts)	Profile (232), promotion (232)
Colorado Mountain College (Colorado)	Profile (28, 241)
Colorado State University (Colorado)	Profile (65, 242), promotion (65, 242), picks (65), priming/prompting (242, 243)
Columbia University in the City of New York (New York)	Profile (106, 244–246), portion (246), picks (246, 247), priming/prompting (248)
Cornell University (New York)	Profile (28, 99, 249–252), portion (118, 253)
Davidson College (North Carolina)	Profile (28)
DePaul University (Illinois)	Profile (254)
Drexel University (Pennsylvania)	Profile (138, 255–257), priming/prompting (138)
Duke University (North Carolina)	Profile (144, 258), promotion (258)
Endicott College (Massachusetts)	Profile (28, 259)
Fairfield University (Connecticut)	Profile (28)
Florida Agricultural and Mechanical University (Florida)	Profile (260)
Florida Institute of Technology (Florida Tech) (Florida)	Profile (261, 262)
Florida State University (Florida)	Profile (263), pricing (122–124), promotion (264), priming/prompting (263, 264)
Framingham State University (Massachusetts)	Profile (28), priming/prompting (265)
Franklin & Marshall College (Pennsylvania)	Profile (28, 266)
Georgetown College (Kentucky)	Profile (28)
Georgia Institute of Technology (Georgia Tech) (Georgia)	Profile (267–269), priming/prompting (267)
Georgia Southern University (Georgia)	Profile (28)
Georgia State University (Georgia)	Profile (65), promotion (65)
Hamilton College (New York)	Profile (270, 271)
Harvard University (Massachusetts)	Profile (27, 107, 272–274), picks (273)
Indiana State University (Indiana)	Priming/prompting (275)
Indiana University Bloomington (Indiana)	Profile (65), promotion (65, 276), priming/prompting (277–279)
Ithaca College (New York)	Profile (28, 280, 281), promotion (280), proximity (280, 281)
John Jay College of Criminal Justice (CUNY John Jay College of Criminal Justice) (New York)	Profile (282)
Johns Hopkins University (Maryland)	Profile (106, 244, 283), promotion (284), priming/prompting (285)
Johnson & Wales University (North Miami campus)^*^ (Florida)	Profile (286), portion (286)
Kent State University (Ohio)	Profile (28, 99, 102, 205, 287–289)
Lee University (Tennessee)	Profile (28)
Lehigh University (Pennsylvania)	Profile (28, 180, 290, 291)
Liberty University (Virginia)	Profile (100, 292)
Loyola Marymount University (California)	Profile (108)
Loyola University New Orleans (Louisiana)	Priming/prompting (293)
Madonna University (Michigan)	Profile (28)
Maharishi International University (formerly Maharishi University of Management) (Iowa)	Profile (191)
Marist University (formerly Marist College) (New York)	Profile (28)
Marquette University (Wisconsin)	Priming/prompting (294)
Michigan State University (Michigan)	Profile (295), portion (295, 296), priming/prompting (295, 297)
Minnesota State University, Mankato (Minnesota)	Profile (108)
Montclair State University (New Jersey)	Profile (298, 299), promotion (298)
Moravian University (Pennsylvania)	Profile (28)
Muhlenberg College (Pennsylvania)	Promotion (300), priming/prompting (300, 301)
Nazareth University (formerly Nazareth College) (New York)	Profile (28)
New York University (New York)	Profile (27, 302–304), promotion (279, 302), priming/prompting (302), proximity (304)
North Carolina State University (North Carolina)	Profile (65, 305, 306), promotion (65), picks (65), priming/prompting (305, 307)
Northeastern University (Massachusetts)	Profile (233, 234, 254, 308, 309), portion (254), priming/prompting (308, 310)
Northern Arizona University (Arizona)	Profile (311), promotion (311, 312), priming/prompting (311, 312), proximity (146)
Northern Kentucky University (Kentucky)	Priming/prompting (181)
Northern Michigan University (Michigan)	Profile (28, 313), priming/prompting (313)
Northwestern University (Illinois)	Profile (306, 314–318), promotion (317)
Ohio University (Ohio)	Profile (109), promotion (109)
Oklahoma City University (Oklahoma)	Profile (110)
Oregon State University (Oregon)	Profile (28, 65, 144, 319), portion (65, 118), promotion (65, 319), picks (65), priming/prompting (319, 320)
Providence College (Rhode Island)	Profile (321, 322)
Quinnipiac University (Connecticut)	Profile (132), promotion (132)
Rice University (Texas)	Profile (96), promotion (96)
Rider University (New Jersey)	Profile (88, 323)
Roanoke College (Virginia)	Profile (28)
Rochester Institute of Technology (New York)	Profile (324)
Rollins College (Florida)	Profile (28)
Rutgers, The State University of New Jersey (Rutgers University) (New Jersey)	Profile (65, 325–328), promotion (65, 327)
Salisbury University (Maryland)	Profile (329, 330), promotion (329–331), priming/prompting (329–331)
San Diego State University (California)	Profile (223, 332–335), promotion (65), priming/prompting (65, 332)
Seattle Pacific University (Washington)	Profile (336)
Seattle University (Washington)	Profile (100, 147, 215, 337), portion (147), priming/prompting (147), proximity (147)
Skidmore College (New York)	Profile (90, 91, 306, 338, 339), priming/prompting (340, 341)
Smith College (Massachusetts)	Profile (28, 205, 342)
Southern University and Agricultural & Mechanical College (Louisiana)	Profile (343)
St. John Fisher University (New York)	Profile (233, 234, 344)
Stanford University (California)	Place (84, 85), profile (84, 85, 174, 345–348), portion (107, 118, 174), promotion (174), picks (107, 174), priming/prompting (174, 348), proximity (84, 85, 174)
Stony Brook University (New York)	Profile (28), promotion (100, 133), priming/prompting (100, 133)
Syracuse University (New York)	Profile (191, 349–352), promotion (350, 352), priming/prompting (349, 350, 353)
The Ohio State University (Ohio)	Profile (65, 106, 115–117, 354–356), pricing (115), promotion (65), picks (65), priming/prompting (116)
The Pennsylvania State University (Penn State) (Pennsylvania)	Profile (65, 100, 357, 358), portion (357), promotion (357), priming/prompting (357)
The University of Arizona (Arizona)	Profile (65, 359, 360), portion (65), pricing (65), promotion (65), picks (65)
The University of Iowa (Iowa)	Profile (124, 129, 130), promotion (129, 130), priming/prompting (361)
The University of Oklahoma (Oklahoma)	Profile (65, 362), promotion (65)
The University of Texas at Austin (Texas)	Profile (27, 28, 65, 205, 363–365), portion (65), promotion (65, 363)
The University of Utah (Utah)	Profile (366, 367), priming/prompting (366)
Towson University (Maryland)	Profile (368–371), promotion (368)
Tulane University of Louisiana (Louisiana)	Profile (28, 106)
University at Albany (New York)	Profile (372, 373), promotion (372), priming/prompting (372)
University at Buffalo (New York)	Profile (65), promotion (65), priming/prompting (374–376)
University of California, Berkeley (California)	Profile (28, 65, 99, 107, 144, 244, 377, 378), portion (65, 142), promotion (65), picks (65), priming/prompting (65, 142, 143, 379)
University of California, Davis (California)	Profile (65, 119, 380), portion (65, 119), promotion (65), picks (65), priming/prompting (119), proximity (119)
University of California, Irvine (California)	Place (86), profile (381), pricing (105), priming/prompting (381)
University of California, Los Angeles (California)	Profile (28, 65, 92, 93, 205, 382–389), portion (65, 384), promotion (65, 92), priming/prompting (57, 65, 144, 145, 382, 390)
University of California, Riverside (California)	Profile (28, 131, 391), portion (118), promotion (131)
University of California, San Deigo (California)	Profile (28, 65, 94, 97, 98, 106, 109, 110, 335, 392, 393), portion (65), pricing (65), promotion (65, 392), picks (65), priming/prompting (392, 394)
University of California, Santa Barbara (California)	Profile (95, 102, 144), promotion (95)
University of California, Santa Cruz (California)	Profile (94, 395), promotion (94)
University of Central Florida (Florida)	Profile (94), priming/prompting (396)
University of Colorado Boulder (Colorado)	Profile (65, 144), portion (65, 118), promotion (65), picks (65), priming/prompting (397)
University of Colorado Colorado Springs (Colorado)	Profile (398, 399), priming/prompting (400)
University of Connecticut (Connecticut)	Profile (65, 321, 401, 402)
University of Dayton (Ohio)	Profile (28, 358, 403–406)
University of Florida (Florida)	Profile (108, 407)
University of Georgia (Georgia)	Profile (65), portion (65), promotion (65), picks (65)
University of Hawaii at Mānoa (Hawaii)	Profile (408), priming/prompting (408)
University of Illinois at Urbana-Champaign (Illinois)	Profile (65, 296, 409), priming/prompting (409)
University of Maine at Presque Isle (Maine)	Profile (213), priming/prompting (410)
University of Maryland, College Park (Maryland)	Profile (65, 94, 107, 188, 411, 412), portion (65), promotion (65, 411, 412), priming/prompting (411)
University of Massachusetts Amherst (Massachusetts)	Profile (99–102, 413–417), promotion (416), priming/prompting (418, 419)
University of Miami (Florida)	Profile (420–422), priming/prompting (421–423)
University of Michigan-Ann Arbor (Michigan)	Profile (28, 65, 84, 100, 177–179, 358, 424), promotion (65), picks (65, 179), priming/prompting (179, 425–427)
University of Minnesota Duluth (Minnesota)	Profile (99, 102), priming/prompting (428)
University of Nebraska-Lincoln (Nebraska)	Profile (65, 429), promotion (65)
University of New Hampshire (New Hampshire)	Profile (430), portion (118), promotion (136, 137, 431), priming/prompting (431)
University of North Carolina at Chapel Hill (North Carolina)	Profile (432)
University of North Texas (Texas)	Profile (28, 65, 105–114), Portion (65), promotion (65)
University of Notre Dame (Indiana)	Profile (254), priming/prompting (433, 434)
University of Oregon (Oregon)	Profile (435, 436), priming/prompting (435, 437)
University of Pennsylvania (Pennsylvania)	Profile (438), priming/prompting (438, 439)
University of Pittsburgh (Pennsylvania)	Profile (440–443), priming/prompting (294, 441)
University of Portland (Oregon)	Profile (11)
University of Rochester (New York)	Profile (444), promotion (444)
University of San Diego (California)	Profile (335, 445, 446), priming/prompting (445, 447)
University of South Carolina (South Carolina)	Profile (192)
University of South Florida (Florida)	Profile (125), pricing (125)
University of Southern California (California)	Profile (65, 448), portion (65, 118, 448), pricing (126–128), promotion (65), picks (65), priming/prompting (448)
University of Vermont (Vermont)	Profile (139, 449), priming/prompting (139, 140, 449, 450)
University of Virginia (Virginia)	Profile (191, 451–454), promotion (451), priming/prompting (451, 453, 454)
University of Washington (Washington)	Profile (65, 134), promotion (65, 134), priming/prompting (65)
University of Wisconsin-Madison (Wisconsin)	Profile (28, 65, 99, 100, 102, 358), promotion (65)
University of Wisconsin-Oshkosh (Wisconsin)	Profile (135), promotion (135)
University of Wyoming (Wyoming)	Profile (455), proximity (455)
Vanderbilt University (Tennessee)	Profile (99, 102, 120, 239, 296, 456), portion (120, 456), priming/prompting (456)
Villanova University (Pennsylvania)	Profile (457–459), portion (460), priming/prompting (458, 460)
Virginia Polytechnic Institute and State University (Virginia Tech) (Virginia)	Profile (461–463), promotion (65, 461, 463)
Wake Forest University (North Carolina)	Profile (121, 148, 464), portion (121, 148), priming/prompting (121), proximity (148)
Washington State University (Washington)	Profile (28, 65), pricing (65), promotion (65), picks (65), priming/prompting (465), proximity (65)
Wellesley College (Massachusetts)	Profile (314)
Western Oregon University (Oregon)	Profile (28, 466)
Williams College (Massachusetts)	Profile (27, 89, 467–469)
Yale University (Connecticut)	Profile (103, 104, 296, 470, 471), promotion (470), priming/prompting (470)

Higher education institution name is listed as displayed on the up-to-date university or college website and/or included evidence; AASHE STARS ratings are based on the overall submission and not specific to food and dining categories of STARS.

^*^Johnson & Wales University North Miami campus closed in 2021 (Johnson & Wales University, n.d.).

MMCA: Marketing-mix choice architecture.

### 3.1 Place

Two higher education institutions (1.20%) used place strategies to encourage customers to select plant-rich menu options. Stanford University encouraged customers to select fresh vegetables by using rotisseries in dining halls to stimulate customers' multisensory experiences (i.e., sight and smell) and replaced meats with whole and cut up vegetables to showcase visually appealing menu options aligning with a plant-rich dietary pattern (84, 85). The University of California, Irvine altered the ambiance of an on-campus food retailer called the “Anteatery” by showcasing the campus garden outside of the food retail establishment and encouraging customers to be “planteaters” (86).

### 3.2 Profile

Nearly all the identified higher education institutions (*n* = 161;96.99%) used one or more profile strategies to encourage customers to select plant-rich menu options. Common applications included: voluntary commitments to increase the proportion and ratio of plant-rich menu options; alterations to the weekly menu options with meatless and/or plant-rich designated days; expansion of plant-rich menu options; and introduction of plant-rich food retail concepts. Examples are discussed below.

#### 3.2.1 Commitments to increase the ratio of plant-rich menu options

Higher education institutions made commitments to increase the number or ratio of meals aligning with a plant-rich dietary pattern. Higher education institutions committed to the Coolfood pledge to decrease food-related GHGE by 25% by 2030 and shifting toward plant-rich food provisions (27) and/or the Forward Food pledge to transition approximately 30% of meat-based entrees to plant-based entrees through the end of 2027 (28).

#### 3.2.2 Implementation of meatless and plant-rich designated days as a profile strategy

Several of the higher education institutions implemented a meat-free and/or plant-rich day to alter the product profile of menu offerings by increasing the plant-rich menu options, reducing the animal-sourced menu options, or exclusively serving meatless menu options once a week across at least one dining setting. Higher education institutions frequently adopted Meatless Monday, a global movement to encourage people to reduce their meat consumption once a week to support human and planetary health, (87) by reducing animal-sourced menu options and increasing plant-rich menu options on Mondays.

Higher education institutions implemented several other meatless day initiatives such as: Rider University's “Wellness Wednesday,” the University at Buffalo's “Plant-Powered Mondays,” and Williams College's “Plant-Rich Mondays” by increasing plant-rich menu options without eliminating meat options (65, 88, 89). Skidmore College's “Low Impact Wednesday,” University of California, Los Angeles's “Beefless Thursdays,” University of California, Santa Barbara's “Climate Friendly Monday,” University of California, Santa Cruz's “Meat-Free Monday,” and University of Southern California's “EcoMonday” focused on reducing animal-sourced menu options (65, 90–95).

#### 3.2.3 Expanding vegan, vegetarian, and plant-based meat recipes and menu options

Higher education institutions frequently expanded whole food vegan and vegetarian menu options or introduced commercial and house-made plant-based meat alternatives (PBMA) to develop plant-rich menu options comparable with animal-sourced menu options. The culinary team at Rice University altered the taste and texture of plant-rich ingredients to develop a vegan brisket menu item (96). The University of California, San Diego's Plant Power Fast Food menu focused on vegan-fast food classics (i.e., burgers, chicken tenders, and milkshakes) (97, 98).

Higher education institutions partnered with food industry stakeholders to procure PBMA and expand plant-rich menu options. The University of Massachusetts, Amherst partnered with Nestle Professional and Wholesome Crave through the Purpose-Driven Plant-Based Incubator to introduce sustainable and plant-rich menu options across university and college foodservice settings (99–101). Additionally, Cornell University, the University of Minnesota Duluth, Kent State University, Vanderbilt University, the University of California, Berkeley, the University of California, Santa Barbara, and the University of Wisconsin-Madison participated in the Purpose-Driven Plant-Based Incubator program to expand plant-rich menu options on campus (99, 102). Yale University partnered with Beyond Meat and Nestle to offer PBMA and expand their plant-rich menu options (103, 104).

#### 3.2.4 Introduction of plant-rich food retail concepts

Higher education institutions commonly introduced new plant-rich food retail concepts as a strategy to expand plant-rich menu options. For instance, the University of North Texas opened the first all-vegan dining concept, Mean Greens, setting an example for higher education foodservice operations across the US (65, 105–114). Similarly, The Ohio State University introduced an on-campus food truck called Thyme & Change 2.0 that featured a vegan menu with plant-rich counterparts to popular animal-sourced menu options (106, 115–117).

### 3.3 Portion

Twenty-six higher education institutions (15.66%) reduced or standardized the portion of animal-source foods within menu options to encourage the consumers to select plant-rich menu options. Higher education institutions commonly procured beef and mushroom blend burgers as a portion strategy. Plant-rich burgers ranged from 25% mushrooms to a 50/50 blend of mushroom and beef patties (65, 107, 118–121). The University of Southern California reduced animal proteins to no more than four ounces per servings across some food retail stations on campus to decrease the portion sizes of animal-sourced foods by designating dining staff to serve customers their portions (65).

### 3.4 Pricing

Nine higher education institutions (5.42%) used pricing strategies to encourage customers to select plant-rich menu options that included: introducing plant-rich menu options at comparable rates compared to animal-sourced products, establishing a discount for plant-rich options, or adding a surcharge on animal-based options.

Higher education institutions initiated weekly price promotions to encourage customers to select plant-rich menu options once a week. Florida State University offered $1 off meals for students who participated in Meatless Monday (122–124). The University of South Florida and the University of California, Irvine offered coupons or discounts for meat-free options on Mondays, yet there was no evidence specifying the amount discounted (105, 125). The University of Arizona implemented $5 Fridays at a plant-forward dining hall on campus to encourage customers to select plant-rich menu options (65).

Some campus dining services strategically used surcharges to encourage customers to select plant-rich menu options. The University of California, San Diego removed the surcharge for plant-based milks and Washington State University encouraged plant-rich defaults with the option to add meat or dairy for a surcharge (65). Additional pricing strategies included the introduction of new plant-rich menu options to counterpart animal-sourced options at a comparable price (i.e., The Ohio State University); (115) offering free vegan meals once a week to students, staff, and faculty who volunteer once a semester (i.e., University of Southern California); (126–128) and using pricing incentives at plant-based retail stations in which plant-based menu options were priced fifty cents less than animal-sourced meals (i.e., Canisius University) (105).

### 3.5 Promotion

More than a third (*n* = 66; 39.76%) of higher education institutions used promotion strategies or initiated marketing campaigns to encourage customers to select plant-rich menu options including: encouraging meatless days, using social media, and informing customers about the benefits of plant-rich dietary patterns.

#### 3.5.1 Encouraging meatless days as a promotional strategy

Higher education institutions used programs such as meat-free or plant-rich days by altering the product profile of campus food provisions. Moreover, the Meatless Monday concept has also been applied as a promotional campaign. Higher education institutions have adopted Meatless Monday concepts as an educational opportunity to promote a “different way of eating,” encourage customers to consider the environmental impacts of meat, and eliminate meat from their diet once a week (94, 129–131). Higher education institutions also used the Meatless Monday concept as a promotional campaign through marketing strategies such as implementing vegetarian specials and promoting plant-rich menu options on social media platforms and websites (105, 131, 132).

#### 3.5.2 Using social media

Higher education institutions used social media platforms to promote plant-rich menu options and initiatives. For example, Rice University's culinary team used marketing tools and the Instagram hashtag #ourplantbasedjourney as a promotion strategy to encourage students to try new plant-rich menu options (96). Ohio University partnered with the football team to create social media videos promoting plant-rich menu options (109). Stony Brook University teamed up with CulinArt dining services to create a Plant-based Eating Road Map to encourage customers to select plant-rich menu options on campus and promoted it through email, social media, and websites (100, 133).

#### 3.5.3 Informing customers about the benefits of a plant-rich dietary pattern

Higher education institutions presented customers with information about the benefits of a plant-rich dietary pattern in the campus dining environment. Higher education institutions such as The University of Texas at Austin and the University of Washington used informational displays to highlight plant-based offerings (65, 134). The University of Wisconsin-Oshkosh participated in the Meatless Monday program by presenting information about the benefits of reducing meat on television and kiosks in the dining environment (135). The University of New Hampshire displayed the “Wildcat Plate” throughout campus providing customers with information on portion control and guidance to “reduce meat and choose fish, poultry, beans, nuts, and seeds” (136, 137).

### 3.6 Default picks

Seventeen higher education institutions (10.24%) used default picks strategies to encourage customers to select plant-rich menu options. We identified most higher education institutions applying plant-rich defaults through the 2025 Humane World for Animals College and University Protein Sustainability Scorecard. These higher education institutions used plant-rich defaults with the option for customers to add meat and/or dairy within at least one dining hall or station (65).

### 3.7 Priming or prompting

Higher education institutions used written or visual priming/prompting strategies by providing contextual information to help students, staff, and faculty at the point-of-purchase to encourage plant-rich menu options or discourage animal-sourced options. Seventy-five higher education institutions (45.18%) used menu icons, symbols, or labeling to identify, indicate, or signify vegan, vegetarian, and plant-rich menu options or applied carbon footprint labeling to encourage customers to select plant-rich menu options.

#### 3.7.1 Vegan, vegetarian, and plant-rich menu labeling

Over one third of higher education institutions identified in this review applied priming and prompting strategies by identifying and labeling vegan and/or vegetarian menu options through a menu icon or label represented at the point-of-purchase or point-of-selection. Some higher education institutions specifically labeled menu items as plant-rich, plant-based, or plant-forward. Stony Brook University used signage displaying “I'm Plant Based” to identify plant-rich menu options (100, 133). Drexel University and the University of Vermont identified plant-based menu options with a leaf symbol (138–140).

#### 3.7.2 Carbon footprint labeling

Our evidence review demonstrated that less than 10% of US higher education institutions applied carbon footprint labels in the context of encouraging customers to select plant-rich menu options. At least seven higher education institutions applied the Coolfood Meals badge in their dining environment identifying low impact menu options. Certified by the World Resources Institute, the Coolfood Meals badge communicates to customers which menu options have a low carbon footprint and environmental impact (141). Other higher education institutions applied their own carbon footprint labeling system. Notably, the University of California, Berkeley, applied a stoplight logo system identifying high-impact foods (i.e., red) compared to low-impact foods (i.e., green) (65, 142, 143). Similarly, the University of California, Los Angeles used green low carbon footprint icons and Earth symbols to identify menu items with 0%−25% of the Daily Value Dietary Carbon Footprint (i.e., vegan menu items, some vegetarian menu items, and certified sustainable fish) and orange-red high carbon footprint icons and Earth symbols to identify menu items with more than 50% of the Daily Value Dietary Carbon Footprint (i.e., beef, lamb, bison, and menu items with more than 3.5 ounces of cheese) to align with the EAT-*Lancet* Commission planetary health diet (144, 145).

### 3.8 Proximity

Ten higher education institutions (6.02%) applied proximity strategies by making plant-rich menu options easy to locate within the dining environment and prominently displaying plant-rich menu options to encourage customers to select plant-rich menu options. One example includes Northern Arizona University's efforts to make vegan and vegetarian menu options easy to locate at their Plant Forward dining station with the intention of customers seeing the plant-rich menu options first (146). Similarly, Stanford University applied choice architecture through the proximity of a 28 foot long “performance bar” near the entrance of the dining hall to encourage students to select plant-rich menu options (84, 85). Seattle University, University of California, Davis, and Wake Forest University placed plant-rich menu options before animal-sourced menu options on menus or buffet lines to encourage the selection of plant-rich menu items (119, 147, 148).

## 4 Discussion

This scoping review identified a total of 363 reports that described 166 higher education institutions that adapted one or more MMCA strategies to encourage customers, such as students, faculty, and staff, to select plant-rich menu options between 2010 and 2024. Among these institutions, 2 (1.2%) were categorized as very small, 32 (19.3%) were categorized as small, 54 (32.5%) were categorized as medium, 45 (27.1%) were categorized as large, and 32 (19.3%) were categorized as very large. The higher education institutions spanned across 36 US states and the District of Columbia. Most higher education institutions (47.0%) were highly residential, primarily residential (33.1%), or residential (15.7%). However, some institutions were online and on-campus learning (1.8%), graduate-focused (0.6%), mostly full-time non-residential (0.6%), or primarily non-residential (0.6%). Institutions most commonly applied profile (96.99%) followed by priming/prompting (45.18%), promotion (39.76%), portion (15.66%), default picks (10.24%), proximity (6.02%), pricing (5.42%), and place (1.20%) strategies in university and college food environments.

There are approximately 6,000 higher education institutions in the US (17, 18). Moreover, our research outlines the extent that a subset of higher education institutions used MMCA strategies to support nutrition and sustainability foodservice goals and dietary transitions in the real-world. The findings contribute to the current research on behavioral economics approaches by documenting real-world US higher education institutions and their innovative strategies to encourage shifts to plant-rich dietary patterns, which offers evidence-based insights for foodservice managers seeking to integrate sustainability and nutrition goals with menu and marketing strategies.

Previously published systematic reviews that evaluated the evidence on behavioral economics strategies to decrease animal proteins and/or increase plant proteins revealed that menu redesign and increasing the availability of plant-rich menu items (i.e., altering the product profile) and menu labeling to provide information about menu items (i.e., priming/prompting) were promising strategies to influence customers to select plant-rich menu options (61, 62). Other evidence describes applying default picks, decreasing animal portions in a “normal range,” and proximity strategies as promising avenues to encourage healthy and sustainable dietary patterns (149, 150).

Our results demonstrated US higher education institutions commonly used profile (96.99%) and priming/prompting (45.18%) strategies. This aligns with evidence describing the use of behavior change strategies to alter the availability of plant-rich menu options and provide information about plant-rich menu options to influence customers' menu selection. These results demonstrate the potential feasibility for the higher education foodservice industry to prioritize sustainability and adopt profile and priming/prompting strategies. Many of the higher education institutions identified by our research frequently used profile and priming/prompting strategies demonstrating that they may be promising strategies for this demographic.

### 4.1 Profile as a feasible, impactful, and cost-effective strategy to encourage plant-rich menu options

Higher education institutions are unique from other institutions procuring food, such as public schools, because students are paying customers and important stakeholders who influence foodservice management procurement decision-making (16, 151). This represents the importance for institutional foodservice businesses to be responsive to customer expectations and purchasing patterns.

Nearly all higher education institutions identified in this review used profile strategies to encourage customers to select plant-rich menu options. The World Resources Institutes' Playbook for Guiding Diners Toward Plant-Rich Dishes in Food Services outlined behavioral science strategies to encourage consumers to select plant-rich menu options and included foodservice industry experts' input on the identified strategies (152). This research described several product profile strategies scoring above the median values for both feasibility and impact criteria. Moreover, shifting to sustainable menu procurement can be financially beneficial for higher education institutions (153, 154). It has been estimated that food costs for plant-rich meals are on average 30% cheaper than meat-based meals and a medium-sized university could save approximately $650,000 annually on food procurement costs by adopting 100% plant-rich procurement plans (153).

The high-feasibility, high-impact, and low-cost of product profile strategies could be one explanation as to why nearly all the identified higher education institutions used profile strategies. This provides insight for higher education foodservice management teams that aim to transform and enhance their menu options while also aligning new recipes with sustainability and nutrition goals. Our study demonstrated that altering the product profile and changing the nutritional content, quality, smell, taste, texture, and flavor to expand plant-rich menu options is a commonly used and standard strategy among higher education foodservice management. This outlines an important trend in sustainability-focused foodservice procurement that researchers and foodservice decision-makers can use as a guide when developing and re-designing menus across higher education settings.

Several higher education institutions applied profile strategies by expanding plant-rich menu options, but strategies varied from specific commitments to increase the ratio of plant-rich meals to less explicit descriptions of expanding or introducing plant-rich recipes. Higher education institutions that made explicit and specific commitments to increase the ratio of plant-rich menu options often committed to an increase of approximately 30%−55%. The University of Colorado Boulder made the largest commitment to make 75% of menu options plant-rich by 2025 (65).

An online randomized control trial demonstrated profile and availability interventions increasing the ratio of vegetarian menu options were most effective to influence meat eaters' food choices when 75% of menu options were vegetarian (155). Foodservice settings, such as higher education dining environments, should offer predominantly plant-rich menu options to encourage significant shifts toward sustainable menu selection without restricting customers' choices. Higher education institutions must evaluate the extent in which their commitments to increase the ratio of plant-rich meals have influenced customers' menu selection in campus dining environments and determine if the commitments need to be adjusted to achieve institutional long-term climate, sustainability, and nutrition goals. These findings suggest that altering product availability and menu composition may be a feasible and high-impact approach for foodservice operators, as such strategies require minimal infrastructure change yet directly shape consumer choice at the point-of-purchase. Moreover, it is important for researchers and foodservice management to evaluate the acceptability of new menu options among their customers (156).

### 4.2 Menu labeling and priming/prompting customers to select plant-rich menu options

Menu labeling may influence individual food choices by providing customers with calorie or nutrition information, contextual information (i.e., daily calorie recommendation), food information (i.e., ingredients, allergy alerts, or symbols to signify healthy foods), or traffic light labeling (157–159). Higher education institutions often applied priming/prompting strategies by using descriptive (i.e., providing nutrient information) and evaluative (i.e., providing interpretive nutrition information or adding special icons and semiotics to menu options) food labeling to influence customers' food knowledge to make informed and rational food decisions at the point-of-purchase (41, 160).

Our study determined higher education institutions commonly applied priming and prompting using vegan and vegetarian menu labeling through a menu icon system offering customers food-related information and aiming to influence their selection of plant-rich menu options. Although menu labeling with point-of-selection nutrition information could be a cost effective and feasible strategy for foodservice management, (161, 162) empirical evidence demonstrates vegan and/or vegetarian labels may not be an effective strategy in foodservice settings to encourage customers to select plant-rich meals (155, 163, 164).

An online randomized control trial tested the impact of a “V” symbol to influence meat eaters' food choice and revealed no significant impact on menu selection (155). Field studies at US higher education institutions determined participants were less likely to select menu items when they were labeled as vegan or vegetarian (163). Other researchers concluded food labels such as “healthy,” “sustainable,” and “healthy and sustainable” were a more effective strategy to encourage consumers to select plant-rich options compared to “vegan” labels (164). Although some researchers recommend removing vegan and vegetarian labels, (163) this could present challenges for institutional foodservice management that use vegan and vegetarian labels to provide transparency to customers that do not consume meat for dietary, ethical, lifestyle, or religious reasons.

Adding qualitative information (i.e., healthy-food symbols and traffic-light systems) may be a promising strategy to promote healthy eating in foodservice settings (157, 159). For instance, Kim et al. (159) determined physical activity-based labeling was a more effective strategy to encourage customers to select healthier foods compared to numeric or color-coded labeling. Additionally, traffic-light labeling may be an effective strategy to encourage healthy and discourage unhealthy menu options in some foodservice settings (165). At least five higher education institutions applied qualitative menu labeling techniques by adapting a rating system in which plant-rich menu options were highlighted as a healthy and sustainable option compared to meat options. Comprehensive labeling strategies to encourage nutrient-dense plant-rich menu options and discourage animal-sourced menu options could provide customers with contextual information to make choices supporting human and planetary health. Yet, more evidence outlining the effectiveness of carbon footprint labeling may be needed for foodservice managers to make an informed and evidence-based decisions about plant-rich menu labeling.

In the context of encouraging plant-rich menu options, some evidence demonstrated traffic-light labels, in which all meat dishes labeled as “High CO_2_,” all fish dishes labeled as “Medium CO_2_,” and all vegetarian dishes labeled as “Low CO2,” decreased meat sales in university cafeteria settings in the short-term (166). On the contrary, an evaluation of traffic-light style climate change menu labels, educational events, and promotional events in a university dining hall resulted in no significant changes in food procurement, student diet quality, or consumption of plant-based foods (167). While carbon footprint labeling is an important strategy to increase customers awareness of the impact of food choices, foodservice management should consider applying multiple MMCA strategies in dining settings during operational planning. Foodservice management should use evidence-based tools and behavior change theories when developing nutrition and sustainability labeling systems and examine consumer behaviors toward the labeling (158).

### 4.3 Encouraging plant-rich menu options through effective promotional messages

Our evidence review identified 66 higher education institutions that applied promotional strategies. The extent to which these higher education institutions applied promotional strategies varied and there was little evidence describing the details of specific messaging that higher education institutions used in campaigns and marketing tools. There is a growing body of literature describing the importance of message framing when developing marketing communication tools to promote plant-rich dietary patterns. Researchers should engage with foodservice leadership to test, adopt, and scale effective promotional messages to encourage plant-rich menu options.

Pro-environmental message framing, social message framing, and neutral message framing have been demonstrated to increase the likelihood of vegetarian menu selection compared to vegetarian framed messages (i.e., “Vegetarian Main Courses”) (168). Similarly, an evaluation of sustainability messages to encourage customers to select vegetarian meals demonstrated descriptive messages communicating the benefits of plant-rich dishes influenced the participants' selection (169). Several higher education institutions described the benefits of plant-rich dietary patterns as a promotional strategy demonstrating that this could be a trend among foodservice management teams.

Taste-focused and appealing menu labeling can encourage customers to select plant-rich menu options (170–172). In university settings, attractive and taste-focused vegetable labeling (i.e., “Herb n'Honey Balsamic Glazed Turnips” vs. “Healthy Choice Turnips”) increased consumers selection and consumption of vegetable menu options (172). These results demonstrated the importance of menu descriptions, message framing, and strategically promoting the taste and appeal of sustainable foods when designing menus.

Promotional messaging could be an effective and low-cost strategy that should be applied across foodservice settings, such as higher education dining environments. However, an examination of menus and their effectiveness to nudge sustainable food options demonstrated some menu design strategies (i.e., recommendation and descriptive menus) increased the likelihood of vegetarian dish choices for infrequent eaters of vegetarian foods but led to the reverse effect on frequent vegetarian eaters (173). This indicates the importance for foodservice management to understand consumers' past eating behaviors when designing MMCA strategies and sustainability-focused foodservice marketing.

The specific ways that higher education institutions used message framing and applied promotional strategies was often unclear throughout our evidence representing a gap in the real-world evidence on how universities and college use messaging to promote plant-rich menu options. However, some higher education institutions described explicit promotional strategies that aligned with empirical evidence on plant-rich menu promotion. For example, Quinnipiac University used social media to share pro-environmental messages such as “cut Broccoli, not rainforests; protect the rainforests from becoming land for grazing cattle; eat more plant-based meals, good for you, good for the planet; and save water, producing $\frac{1}{4}$ pound of hamburger requires an astounding 449 gallons of water, preserve our water sources” (132). Notably, Stanford University has applied taste-focused labeling as a promotional strategy to reframe plant-rich menu items as flavorful, fun, and delicious (174). Future research could assess the use of taste-focus labeling across higher education institutions' menu boards to understand the implementation of this strategy.

### 4.4 Meatless days and plant-rich designated days

Higher education institutions used Meatless Monday as a profile strategy by increasing the plant-rich menu options, reducing the animal-sourced menu options, or exclusively serving meatless menu options once a week. However, the results of this study identified some higher education institutions applied the Meatless Monday concept as a promotional, pricing, or priming/prompting strategy.

When applied as a promotional strategy, higher education institutions used a variety of marketing materials to promote or feature plant-rich menu options on meatless or plant-rich designated days. An evaluation of Meatless Monday messaging revealed health-focused and environment-focused messages were an effective communication strategy to attract participants attention, increase the negative perception of meat consumption, influence consideration of the health and environmental impacts of meat consumption, and stimulate participants' interest in talking about the Meatless Monday campaign (175). This evidence revealed applying meatless or plant-rich day concepts using promotional strategies could be an impactful approach to reduce US meat consumption. Higher education institutions should evaluate their current promotional efforts to encourage meatless or plant-rich designated days to understand if the health-focused and environment-focused messages are influencing customers' food selection at the point-of-purchase and consumption.

Although higher education institutions identified in this review commonly adopted a meat-free day (i.e., Meatless Monday), our review also found resistance from customers at some higher education institutions after offering a meat-free day representing barriers for implementation. For instance, California State University, Chico attempted to adopt Meatless Monday to encourage customers to reduce meat, but due to push back from students and faculty, the dining services decided to expand vegetarian meals but drop the Meatless Monday concept (176).

In contrast, some higher education institutions, such as the University of California, Santa Cruz, adopted meat-free days due to student encouragement for dining services to exclusively offer vegetarian meals once per week (94). Students at The University of Iowa led the Meatless Monday initiative that was supported through the university's “Climate for Change” themed semester, which resulted in complaints from the Iowa meat and livestock industry and sparked controversy on social media (129, 130).

The University of Michigan-Ann Arbor's adoption of the Meatless Monday concept resulted in fewer customers and negative student feedback (177). Therefore, the University of Michigan-Ann Arbor implemented a modified initiative called “Sustainable Mondays” to serve less red and processed meat (i.e., beef, lamb, goat, pork, and pepperoni) and replace these choices with lower climate-impact proteins (i.e., chicken, fish, and plants) rather than eliminating animal-sourced menu options (177–179). Dining services did not explicitly advertise this initiative resulting in less student resistance (177). This creative solution demonstrates a feasible and acceptable strategy for foodservice decision-makers to address potential challenges to implementing Meatless Mondays by considering stakeholder and customer acceptability.

### 4.5 Future research and recommendations

The findings of this study have major practical implications. We concluded that higher education institutions have made progress to apply behavior change theory to practice to encourage customers to select plant-rich menu options. Specifically, higher education institutions made substantial efforts to expand and introduce menu items that align with a plant-rich dietary pattern through profile strategies. These findings contribute to the knowledge-practice gap by documenting how higher education institutional foodservices are implementing choice architecture and nudge strategies into real-world university and college foodservice settings to encourage healthy and sustainable meal selection. Our findings highlight that there are several opportunities for higher education institutions to comprehensively apply multiple MMCA strategies to promote sustainable dietary patterns in campus food environments. Our results can be applied as a guide for university and college foodservice leadership that aim to improve their campus food environment to support personal and planetary health.

Furthermore, our results contribute to the current academic knowledge on nudge theory application and the use of MMCA strategies across foodservice settings. Our adaption of the MMCA framework adds to the growing body of literature on nutrition and food behavior change interventions across food environments by outlining the extent that higher education institutions are using various MMCA strategies. These findings may be useful for other researchers aiming to explore the application and impact of behavior change models and theory across foodservice settings.

There are numerous opportunities for higher education institutions to procure and promote plant-rich menu options to support planetary health. This study provided a scan of higher education institutions and their use of MMCA strategies. Future research should explore the effectiveness of these strategies on improving students' knowledge, attitudes, and behaviors toward plant-rich dietary patterns, as well as the feasibility, adoption, implementation, and accessibility of these strategies from foodservice directors and managers' perspectives. Future research could conduct surveys, interviews, or focus groups among higher education foodservice directors from the higher education institutions and managers identified through this study to learn about the progress of the MMCA strategies, current initiatives, barriers, and other aspects of sustainable dining and foodservice solutions.

This systematic scoping review identified 166 higher education institutions that made progress to encourage customers to select plant-rich menu options in campus dining environments using interdisciplinary databases and gray literature searches. Our evidence review found some higher education institutions introduced written relevant policies to encourage the selection of plant-rich menu options (i.e., the University of California system, LeHigh University, and Northern Kentucky University), (65, 92, 93, 180, 181) but most of the identified efforts were practices rather than written and formal policies. Future research should conduct a policy scan of US higher education institutions to explore the extent MMCA strategies are enforced through policy change to foster sustainable food environments.

We aimed to explore US higher education institutions and their adoption of MMCA strategies. An evaluation of the effectiveness of MMCA strategies was outside of the scope of this study. Future research is needed to determine the effectiveness of long-term higher education institutional strategies in real-world campus dining environments. Researchers and foodservice leadership could collaborate to explore which MMCA strategies are feasible, impactful, and effective for their university and college community. Participatory research methodology and partnership with higher education stakeholders (i.e., foodservice management, students, and researchers) is an important next step at the community and institutional level. Researchers can partner with these key stakeholders to benchmark the current university and college food environment and recommend promising interventions. The results of this study could be used as a guide for higher education institutions aiming to apply practical behavior change interventions.

Multimodal interventions targeting the conscious decision making and the retail environment have been determined to be an impactful approach to reduce the consumption of meat menu options in university settings (47). MMCA strategies should be combined within a single food environment where people make dietary decisions to influence their health (51). Over half of higher education institutions applied more than one MMCA strategy to encourage customers to select plant-rich foods between 2010 and 2024. An examination of whether and how the 166 higher education institutions applied the MMCA strategies in combination at the same time and in the same setting was outside of the scope of this systematic scoping review. Higher education institutions should consider targeting the consumers' dual decision-making processes by altering the retail environment when applying MMCA strategies for maximum impact (47). An evaluation of the higher education institutions application of multimodal strategies that target both the unconscious and conscious decision-making concurrently would be an important next step for researchers to understand how colleges and universities are strategically applying MMCA strategies.

Our evidence review focused on environmental change strategies used in higher education foodservice and campus food environments to change consumer behavior (i.e., students, faculty, and staff). We only included foodservice providers and their commitments to increase plant-rich recipes if there was evidence that a US higher education institution partnered with the foodservice provider to incorporate plant-rich procurement in their institutional dining setting and food environment. Several foodservice companies (i.e., Guckenheimer, Metz Culinary Management, Sodexo, Fresh Ideas, and Elior North America) committed to increase the plant-rich menu offerings across a range of foodservice settings such as K-12 schools, higher education institutions, corporate offices, sports arenas, and other public facilities (182). Researchers should continue to evaluate the commitments made across different foodservice settings to increase selection and consumption of sustainable foods across the broader food environment and foodservice sector. This evidence could help close the current gap between research and practice for behavior change interventions.

### 4.6 Limitations

There were several limitations to this study. We developed a comprehensive search strategy to capture the recent efforts to encourage customers to select plant-rich menu options in US higher education institutions settings. However, it is likely some strategies were not captured by the evidence search process. This study only captured published efforts among higher education institutions and excluded any ongoing and undocumented efforts. Although the higher education institutions' food and nutrition websites were searched, it is likely these websites are regularly updated. Additional dining services efforts aligning with the MMCA framework may be added to the higher education institutions' websites that were not captured by this review.

We defined profile strategies as institutions changing the nutritional profile, quality, smell, taste, texture, and flavor of menu options to increase the number or ratio of meals that align with a plant-rich dietary pattern. Supplementary File 2 outlines the range of approaches that higher education institutions could apply profile strategies including reformulating, developing, or introducing new menu options; introducing a food retailer with the intention of expanding plant-rich menu options; increasing the ratio of plant-rich menu options served; introducing one plant-rich day per week; and introducing plant-rich alternatives compared to popular animal-sourced menu options. As described in our results section, higher education institutions commonly applied profile strategies by committing to increase the proportion and ratio of plant-rich menu options; altering the weekly menu options with meatless and/or plant-rich designated days; expanding plant-rich menu options; and introducing plant-rich food retail concepts. However, the current study and application of the MMCA framework did not quantify the number of interventions that made alterations to the existing menu options, interventions that added to the existing set of offerings, and interventions that applied profile strategies by both adding new menu options and changing existing menu options. This specific analysis of profile strategies was outside the scope of this study. Due to the large representation of higher education institutions applying profile strategies, further research is needed to understand the range in which foodservice management teams are applying multiple product profile techniques.

Due to the limited peer-reviewed evidence describing real-world and long-term dining initiatives in higher education settings, our review relied on gray literature published between 2010 and 2024. Although the reviewed evidence aligned with the predetermined timeframe, the authors could not assume that multiple MMCA strategies implemented by higher education institutions always occurred at the same time and in one single dining setting (i.e., dining hall). Determining whether the combination of MMCA approaches worked to increase the selection of plant-rich menu options was outside of the scope of this research. However, future research should address whether and how foodservice management has applied MMCA strategies concurrently.

Our evidence review focused solely on US higher education institutions and did not capture global efforts and use of MMCA strategies to encourage plant-rich dietary patterns. Future research should conduct global systematic scoping reviews to learn from other countries and their real-world foodservice innovations and creative sustainability-focused solutions. For example, in the United Kingdom, more than 650 academics urged universities to shift toward 100% plant-based procurement through the Plant-Based Universities campaign (183). Similarly, the École Polytechnique Fédérale de Lausanne has set dining service goals and action items relevant to behavioral economics strategies by serving 80% vegetarian meals by 2030, reducing red meat by 80% by 2030, and promoting plant-based dishes (184).

We did not review social media platforms (i.e., Instagram, Facebook, TikTok, YouTube, etc.) for each of the identified higher education institutions. Thus, marketing and promotional efforts only available via the social media platform were not captured through this review. Future research and scoping reviews could be conducted with the primary aim to explore the extent that higher education institutions are using promotional strategies via social media to promote plant-rich dietary patterns and other sustainable food efforts.

One reviewer (NLF) conducted the title and abstract screening and the supplemental searches, extraction, and categorization for the additional references (i.e., organizational searches and website searches) demonstrating a limitation in the screening process. Due to the study objectives focus on real-world higher education institution initiatives, we excluded one-time studies and experimental research from this review. Thus, the results of this evidence synthesis review were predominantly reports described as news articles, media sources, trade journals, or higher education institution websites. However, the lead author applied supplemental searches to support data triangulation.

## 5 Conclusion

This systematic scoping review identified 166 US higher education institutions that applied one or more MMCA strategies to encourage plant-rich menu selections. Most higher education institutions used profile strategies, followed by priming/prompting, promotion, portion, default picks, proximity, pricing, and place. Over half implemented multiple strategies, yet no higher education institution adopted all eight MMCA strategies comprehensively.

Beyond mapping these applications, the findings highlight how campus dining operations function as foodservice businesses that use marketing, menu engineering, and behavioral design to influence customer choices. For practitioners, understanding which MMCA strategies are most feasible and visible can guide operational planning and marketing communication in sustainability initiatives. From a consumer perspective, these strategies shape how students, faculty, and staff perceive, evaluate, and ultimately choose plant-rich menu options in encouraging sustainable behaviors. For institutional policymakers, campus dining services, and researchers, the results outline innovative, creative, and scalable approaches to align business performance with environmental goals in institutional foodservice while supporting meaningful and lasting dietary change among campus consumers.

## Data Availability

The original contributions presented in the study are included in the article/Supplementary material, further inquiries can be directed to the corresponding author.
