# Supplementary material for: Higher education institutions and the use of marketing-mix choice architecture strategies to encourage plant-rich menu options and sustainable dietary patterns: a scoping review
Source: Front Nutr. 2026 Mar 24;13:1774451. doi: 10.3389/fnut.2026.1774451 (PMC13053224; doi:10.3389/fnut.2026.1774451)
Supplement: Supplementary file 2 [file Supplementary_File_2.pdf]

**Supplemental File 2:** MMCA Framework Adapted to US Higher Education Settings to Encourage Customers to Select Plant-Rich Menu Options, Comprehensive Search Strategy for Systematic Scoping Review, and Final Search Results

**Supplemental Table 1.** MMCA Framework Adapted to US Higher Education Settings to Encourage Plant-Rich Menu Options

| Category | Strategy | Metrics |
|---|---|---|
| Alterations made to the **properties** of the dining service food environment and/or food, beverage and the **product** (i.e., menu options served and sold) to influence students, faculty, and staff's selection and consumption behaviors. | **Place:** Change the internal settings (i.e., lighting) and use sensory cues (i.e., smell or sight) to influence student, faculty, and staff's expectation about the ambience or the atmosphere to highlight food and beverage products that align with a plant-rich dietary pattern. | • Higher education institutions used lighting, visual cues, or sensory cues to highlight plant-rich menu options that align with a plant-rich dietary pattern. |
| | **Profile:** Change the nutritional profile, quality, smell, taste, texture, and flavor of menu options to increase the number or ratio of meals that align with a plant-rich dietary pattern. | • Higher education institutions reformulated, developed, or introduced new menu options to improve the variety, appearance, nutritional profile, quality, smell, taste, texture and flavor of menu options that align with a plant-rich dietary pattern.<br>• Higher education institutions introduced and promoted a stand-alone restaurant or food retailer on campus with the intention of expanding plant-rich menu options.<br>• Higher education institutions increased the relative number or ratio of plant-rich menu options by introducing new entrees, designing new recipes, adding to the existing set of menu options, or creating a greater variety of plant-rich menu options.<br>• Higher education institutions introduced one plant-rich day per week in which plant-rich menu options were increased or only meat-free menu options were served (i.e., *Meatless Monday).<br>• Higher education institutions introduced plant-rich alternatives compared to popular animal-sourced menu options. |

| | | |
|---|---|---|
| | **Portion:** Reduce and/or standardize the portion of food and beverage options (i.e., animal-sourced foods or meals) within a menu option to align with a plant-rich dietary pattern. | • Higher education institutions reduced and/or standardized the portion of animal-sourced foods to align with a plant-rich dietary pattern.<br>• Higher education institutions reduced the amount of animal-sourced ingredients in a dish while increasing the amount of plant-sourced ingredients. |
| | **Pricing:** Use pricing strategies (i.e., proportionate pricing for plant-rich menu options and limiting price promotions on unhealthy and unsustainable menu options) to increase sales and revenue for products that align with a plant-rich dietary pattern. | • Higher education institutions used pricing strategies to increase sales and revenue for products that align with a plant-rich dietary pattern.<br>• Higher education institutions sold plant-rich menu options at a lower price than meat dishes.<br>• Higher education institutions implemented coupons or loyalty points to redeem on plant-rich menu options. |
| | **Promotion:** Use responsible food and beverage marketing practices (i.e., changing the name, appearance, appeal and attractiveness of menu options or food/beverage products) and promote consumer selection of menu options and products that align with a plant-rich dietary pattern. | • Higher education institutions implemented and enforced a policy or program to use responsible food marketing practices to promote menu options and products that align with a plant-rich dietary pattern.<br>• Higher education institutions implemented and enforced a policy or program to restrict the promotion of unsustainable and unhealthy food and beverage options (i.e., animal-source foods) to students, faculty and staff.<br>• Higher education institutions used promotional language on the menu to emphasize positive attributes of a plant-rich menu option.<br>• Higher education institutions used promotional language on the menu to recommend a plant-rich menu options or discourage an animal-sourced menu option.<br>• Higher education institutions promoted the benefits of plant-rich menu options through marketing materials and/or communications on social media on campus or in the dining hall. |
| Adjustments made to the **placement** of food, beverage, and menu options served and sold in the dining service food environment and on campus to influence students, faculty, and staff's purchasing and consumption behaviors. | **Default Picks:** Use environmental cues that are convenient, accepted, and expected to socially normalize healthy and sustainable default menu choices by introducing plant-rich menu options as the default option and animal-sourced options available upon request. | • Higher education institutions implemented and enforced a policy, program, or practice to offer healthy and sustainable default picks (i.e., plant-rich menu options).<br>• Higher education institution only offers plant-rich menu options on the main menu with animal-soured menu options available on request or through a separate menu. |
| | **Priming or Prompting:** Use information (i.e., menu labeling, logos, icons, semiotics or | • Higher education institutions implemented and enforced a policy, program, or practice to include menu labeling, icons, |

| | | |
|---|---|---|
| | contextual information) to influence customers' knowledge and help students, faculty, and staff make healthy and sustainable choices at the point-of-choice and point-of-purchase that align with a plant-rich menu option. | digital boards to guide students, faculty, and staff to dietary selections aligned with a plant-rich menu option.<br>• Higher education institutions implemented a menu icon system to help customers recognize that plant-rich menu options support personal and ecological health.<br>• Higher education institutions added **carbon footprint labels to menus, food labels or display shelves. |
| | **Proximity:** Place plant-rich menu options at eye level and physically closer to customers at the point-of-choice and point-of-purchase. | • Higher education institutions placed plant-rich menu options and products closer to customers' point-of-choice (i.e., buffet lines) and point-of-purchase (cash register) locations.<br>• Higher education institutions manipulated the layout of the dining area to highlight plant-rich menu options and move them to a more visible position.<br>• Higher education institutions introduced a section of the food retailer dedicated to only plant-rich foods and placed it closer to customers. |

Adapted from: Attwood et al. (2020); Kraak et al. (2017)

Note: *Higher education institutions adoption of Meatless Monday or other meat-free days were categorized as profile strategies due to dining services efforts to increase the number/ratio of plant-rich menu options served unless additional evidence described the meat-free day as a promotional effort, educational campaign, or price strategy; **Higher education institutions use of carbon footprint labeling were only included in the results if there was evidence that the labels were developed to encourage customers' selection of low-impact plant-rich menu options and discourage customers' selection of high-impact animal-sourced menu options rather than encouraging sustainable food efforts outside the scope of plant-rich dietary pattern (i.e., local food options)

**Supplemental Table 2.** Comprehensive Search Strategy for Systematic Scoping Review

| Inclusion criteria | Exclusion criteria |
|---|---|
| 1. Peer-reviewed journal articles that report commitments, policies, programs, or practices adopted by a traditional tertiary education setting (i.e., US higher education dining services) to encourage plant-rich menu options through one or more MMCA strategy.<br>2. Gray literature reports that describe commitments, policies, programs, or practices adopted by a by a traditional tertiary education setting (i.e., US higher education dining services) to encourage plant-rich menu options through one or more MMCA strategy.<br>3. Media stories, news releases, and news articles that describes commitments, policies, programs, or practices adopted by a traditional tertiary education setting (i.e., US higher education dining services) to encourage plant-rich menu options through one or more MMCA strategy.<br>4. US higher education website that reports commitments, policies, programs, or practices adopted by universities or college dining services to encourage plant-rich menu options through one or more MMCA strategy.<br>5. Records or reports were published as peer-reviewed articles, book chapters, gray literature reports, or websites between January 1, 2010 and December 31, 2024*.<br>6. Records or reports described commitments, policies, programs, or practices adopted by US higher education dining services.<br>7. Records or reports with full-text English language version available. | 1. Peer-reviewed journal articles, gray literature reports, media stories/news releases, or higher education websites that describe findings from a one-time experimental study or program (i.e., the MMCA strategy was not continued after the research experiment) or annual events that occurred once a year (i.e., celebrating National Nutrition Month or Earth Day with meatless options, annual plant-rich dinners, etc.).<br>2. Only one food, menu item, or recipe change.<br>3. Peer-reviewed journal articles, gray literature reports, media stories/ news releases or higher education website that describe interventions outside of the scope of MMCA (i.e., cooking classes, food and nutrition courses, etc.).<br>4. Peer-reviewed journal articles, gray literature reports, media stories/news releases or higher education websites that describe higher education dining services commitments, policies, programs or practices promoting aspects of sustainable diets or sustainability not related to plant-rich dietary patterns (i.e., composting, food recovery, food security, or local food).<br>5. Peer-reviewed journal articles, gray literature reports, media stories/news releases or higher education websites that mention providing plant-rich menu options but there lacks a clear MMCA promotion or strategy.<br>6. Peer-reviewed journal articles, gray literature reports, or media stories/ news releases that take place outside of tradition university or college setting (i.e., university hospital systems, elementary school, middle school or high school). |

| | |
|---|---|
| *Websites with the copyright date of 2025 were included if the websites described active implementation of MMCA strategies | 7. Records or reports were published before January 1, 2010.<br>8. Records or reports that describe commitments, policies, programs, or practices adopted by higher education dining services outside of the US.<br>9. Records or reports that describe a foodservice company commitment to increase plant-rich menu options without evidence that a higher education institution has adopted it in a dining services setting.<br>10. Full-text English language version of the evidence source was not available.<br>11. Full books describing MMCA strategies tested in college settings.<br>12. Systematic reviews or literature reviews describing MMCA strategies tested in university and college settings.<br>13. Theses, dissertations, patents, conference, or poster abstracts. |
| **Peer-reviewed Literature Search Strategy** | |
| **Five electronic databases:**<br>1. PubMed<br>2. Education Resources Information Center (ERIC)<br>3. Business Source Complete<br>4. ProQuest One Business (ABI/Inform)<br>5. Core Collection | **Search Terms:**<br>Concept 1: **University/college campuses** *(Population)*: (university OR universities OR undergrad OR undergrads OR undergraduate OR undergraduates OR college OR colleges OR collegiate OR "higher education" OR campus* OR "post-secondary" OR postsecondary OR "post secondary" OR "Post-graduate" OR "Post-graduates" OR "post graduate" OR "post graduates" OR "tertiary education")<br><br>AND<br><br>Concept 2: **Plant-rich foods** *(Concept)*:<br><br>((food* OR cater* OR eatery OR eateries OR nutrition OR diet* OR meal* OR dining OR canteen* OR menu* OR cafe* OR lunch* OR breakfast* OR dinner* OR "snack bar*" OR grill* OR restaurant*) |

<table>
<tr><td></td><td>AND<br><br>(“plant-rich” OR “plant rich” OR “plant-based” OR “plant based” OR “plant alternative*” OR “plant-forward” OR “plant forward” OR “plant protein*” OR “plant derived” OR “non-animal protein*” OR vegetable* OR fruit* OR grain* OR soy OR nut OR nuts OR seed* OR tofu OR tempeh OR bean* OR legume* OR vegan* OR vegetarian* OR flexitarian* OR “meat-free” OR “meat free” OR “alternative protein*” OR meatless OR “meat reduction” OR “reduce meat*” OR “reduced meat*”))<br><br>AND<br><br>Concept 3: MMCA Strategies (Context): (policy OR policies OR guideline* OR program* OR commitment* OR initiative* OR campaign* OR standard* OR recommendation* OR intervention* OR nudg* OR “choice architecture*” OR “behavioral economics” OR “default choice*” OR “nutrition profile*” OR “nutrient profile*” OR “nutritional profile*” OR portion* OR promot* OR proximity OR prompting OR pric* OR cost* OR placement OR label* OR “point of purchase” OR “point-of-purchase” OR marketing OR “marketing-mix” OR advertis*)</td></tr>
<tr><td colspan="2">Gray Literature Search Strategy</td></tr>
<tr><td colspan="2">Two Gray Literature Databases:<br>1. Google Scholar (first 300 search hits)<br>Search string: (university AND dining) AND (food OR nutrition OR catering OR "plant-rich" OR "plant-forward" OR "plant-based" OR vegetarian OR vegan OR "meat-free" OR meatless OR commitment OR policy OR program OR practices OR marketing OR nudge OR “choice architecture”)<br>2. Access World News (NewsBank) (first 300 search hits + follow-up search: first 100 search hits)<br>Search string: (university OR universities OR college OR colleges OR campus) AND (“dining services” OR food OR nutrition OR catering OR "plant-rich" OR "plant-forward" OR "plant-based" OR vegetarian OR vegan OR "meat-free" OR meatless OR commitment OR policy OR program OR practices OR marketing OR nudge OR “choice architecture”)</td></tr>
<tr><td colspan="2">Supplemental Search Strategy</td></tr>
</table>

| Supplemental search of targeted higher education sustainability ranking systems, commitments, or programs related to plant-rich menu options | |
|---|---|
| 1. *Humane World for Animals (reviewed May-August 2025)<br>2. The Coolfood Pledge (reviewed May 2025)<br>3. Forward Food Pledge (reviewed May 2025)<br>4. The Menus of Change University Research Collaborative (reviewed May 2025)<br>5. Meatless Monday website and corresponding resources filtered by case studies and success stories (reviewed June 2025)<br>6. **AASHE STARS report among previously identified higher education institutions (reviewed June-August 2025)<br><br>*Humane World for Animals College and University Protein Sustainability Scorecard and AASHE STARS reports were included due to the description of higher education dining services efforts implemented prior to 2025<br>**STARS Ratings are based on the overall submission and should always be communicated in that manner (i.e., ratings are not awarded in specific categories of STARS). The information published through STARS is self-reported. | |
| **Supplemental search of targeted higher education websites**<br>Supplemental search of targeted higher education websites was conducted by the lead researcher, NLF, after the report was included to identify supplemental information to support the MMCA strategy or other promotional efforts. | **Google search string: "higher education name" AND "sustainable OR dining"<br><br>***Google search string: "higher education name" AND "program, policy, or commitment name" if applicable |

**A supplement search of higher education websites or additional media was only conducted among higher education institutions previously identified through the systematic search of records

***A supplemental search of higher education programs, policies, or commitments were only conducted if a clear program, policy, or commitment name was previously identified through the systematic search of records

**Abbreviations and acronyms:** Association for the Advancement of Sustainability in Higher Education (AASHE); Education Resources Information Center (ERIC); Marketing-Mix Choice Architecture (MMCA); Sustainability Tracking, Assessment & Rating System (STARS); United States (US)

**Supplemental Table 3.** Final Search Results

| Database Searched | Database Coverage | Platform/ Manager | Final Search Strategy* | Date Searched | Number of Results |
|---|---|---|---|---|---|
| Initial Search | | | | | |
| PubMed | English; 01/01/2010-11/15/2024 (present date of search)<br><br>Title/Abstract | National Library of Medicine | ((("Universities"[Mesh]) OR (university[Title/Abstract] OR universities[Title/Abstract] OR undergrad[Title/Abstract] OR undergrads[Title/Abstract] OR undergraduate[Title/Abstract] OR undergraduates[Title/Abstract] OR college[Title/Abstract] OR colleges[Title/Abstract] OR collegiate[Title/Abstract] OR "higher education"[Title/Abstract] OR campus*[Title/Abstract] OR "post-secondary"[Title/Abstract] OR postsecondary[Title/Abstract] OR "post secondary"[Title/Abstract] OR "Post-graduate"[Title/Abstract] OR "Post-graduates"[Title/Abstract] OR "post graduate"[Title/Abstract] OR "post graduates"[Title/Abstract] OR "tertiary education"[Title/Abstract])) AND ((((food*[Title/Abstract] OR cater*[Title/Abstract] OR eatery[Title/Abstract] OR eateries[Title/Abstract] OR nutrition[Title/Abstract] OR diet*[Title/Abstract] OR meal*[Title/Abstract] OR dining[Title/Abstract] OR canteen*[Title/Abstract] OR menu*[Title/Abstract] OR cafe*[Title/Abstract] OR lunch*[Title/Abstract] OR breakfast*[Title/Abstract] OR dinner*[Title/Abstract] OR "snack bar*"[Title/Abstract] OR grill*[Title/Abstract] OR restaurant*[Title/Abstract]) OR ("Food"[Mesh] OR "Food Services"[Mesh])) AND ("plant-rich"[Title/Abstract] OR "plant rich"[Title/Abstract] OR "plant-based"[Title/Abstract] OR "plant based"[Title/Abstract] OR "plant alternative*"[Title/Abstract] OR "plant-forward"[Title/Abstract] OR "plant forward"[Title/Abstract] OR "plant protein*"[Title/Abstract] OR "plant derived"[Title/Abstract] OR "non-animal protein*"[Title/Abstract] OR vegetable*[Title/Abstract] OR fruit*[Title/Abstract] OR grain*[Title/Abstract] OR soy[Title/Abstract] OR nut[Title/Abstract] OR nuts[Title/Abstract] OR seed*[Title/Abstract] OR tofu[Title/Abstract] OR tempeh[Title/Abstract] OR bean*[Title/Abstract] OR legume*[Title/Abstract] OR vegan*[Title/Abstract] OR vegetarian*[Title/Abstract] OR flexitarian*[Title/Abstract] OR "meat-free"[Title/Abstract] OR "meat free"[Title/Abstract] OR "alternative protein*"[Title/Abstract] OR meatless[Title/Abstract] OR "meat | 11/15/24 | 1,553 |

| | | | | | |
|---|---|---|---|---|---|
| | | | reduction"[Title/Abstract] OR "reduce meat*"[Title/Abstract] OR "reduced meat*"[Title/Abstract])) OR ("Diet, Vegan"[Mesh] OR "Diet, Vegetarian"[Mesh] OR "Meat Substitutes"[Mesh] OR "Diet, Plant-Based"[Mesh]))) AND (("Policy"[Mesh] OR "Organizational Policy"[Mesh] OR "Marketing"[Mesh] OR "Advertising"[Mesh]) OR (policy[Title/Abstract] OR policies[Title/Abstract] OR guideline*[Title/Abstract] OR program*[Title/Abstract] OR commitment*[Title/Abstract] OR initiative*[Title/Abstract] OR campaign*[Title/Abstract] OR standard*[Title/Abstract] OR recommendation*[Title/Abstract] OR intervention*[Title/Abstract] OR nudg*[Title/Abstract] OR "choice architecture*"[Title/Abstract] OR "behavioral economics"[Title/Abstract] OR "default choice*"[Title/Abstract] OR "nutrition profile*"[Title/Abstract] OR "nutrient profile*"[Title/Abstract] OR "nutritional profile*"[Title/Abstract] OR portion*[Title/Abstract] OR promot*[Title/Abstract] OR proximity[Title/Abstract] OR prompting[Title/Abstract] OR pric*[Title/Abstract] OR cost*[Title/Abstract] OR placement[Title/Abstract] OR label*[Title/Abstract] OR "point of purchase"[Title/Abstract] OR "point-of-purchase"[Title/Abstract] OR marketing[Title/Abstract] OR "marketing-mix"[Title/Abstract] OR advertis*[Title/Abstract])) | | |
| ERIC | English; 01/01/2010-11/15/2024 (present date of search)<br><br>Title/Abstract/ Subject | EBSCOhost | (TI ( university OR universities OR undergrad OR undergrads OR undergraduate OR undergraduates OR college OR colleges OR collegiate OR “higher education” OR campus* OR “post-secondary” OR postsecondary OR “post secondary” OR “Post-graduate” OR “Post-graduates” OR “post graduate” OR “post graduates” OR “tertiary education” ) OR AB ( university OR universities OR undergrad OR undergrads OR undergraduate OR undergraduates OR college OR colleges OR collegiate OR “higher education” OR campus* OR “post-secondary” OR postsecondary OR “post secondary” OR “Post-graduate” OR “Post-graduates” OR “post graduate” OR “post graduates” OR “tertiary education” ) OR SU ( university OR universities OR undergrad OR undergrads OR undergraduate OR undergraduates OR college OR colleges OR collegiate OR “higher education” OR campus* OR “post-secondary” OR postsecondary OR “post secondary” OR “Post-graduate” OR “Post-graduates” OR “post graduate” OR “post graduates” OR “tertiary education” ) OR DE "Colleges" OR DE "College Environment") AND (TI ( food* OR cater* OR eatery OR eateries OR nutrition OR diet* OR meal* OR dining OR canteen* OR menu* OR cafe* OR lunch* OR breakfast* OR dinner* OR “snack bar*” OR grill* OR restaurant* ) OR AB ( food* OR cater* OR eatery OR eateries OR nutrition OR diet* OR meal* OR dining OR canteen* OR menu* OR cafe* OR lunch* OR breakfast* OR dinner* OR | 11/15/2024 | 135 |

| | | | | | |
|---|---|---|---|---|---|
| | | | “snack bar*” OR grill* OR restaurant* ) OR SU ( food* OR cater* OR eatery OR eateries OR nutrition OR diet* OR meal* OR dining OR canteen* OR menu* OR cafe* OR lunch* OR breakfast* OR dinner* OR “snack bar*” OR grill* OR restaurant* ) OR DE "Food" OR DE "Food Service" OR DE "Dining Facilities")) AND (TI ( “plant-rich” OR “plant rich” OR “plant-based” OR “plant based” OR “plant alternative*” OR “plant-forward” OR “plant forward” OR “plant protein*” OR “plant derived” OR “non-animal protein*” OR vegetable* OR fruit* OR grain* OR soy OR nut OR nuts OR seed* OR tofu OR tempeh OR bean* OR legume* OR vegan* OR vegetarian* OR flexitarian* OR “meat-free” OR “meat free” OR “alternative protein*” OR meatless OR “meat reduction” OR “reduce meat*” OR “reduced meat*” ) OR AB ( “plant-rich” OR “plant rich” OR “plant-based” OR “plant based” OR “plant alternative*” OR “plant-forward” OR “plant forward” OR “plant protein*” OR “plant derived” OR “non-animal protein*” OR vegetable* OR fruit* OR grain* OR soy OR nut OR nuts OR seed* OR tofu OR tempeh OR bean* OR legume* OR vegan* OR vegetarian* OR flexitarian* OR “meat-free” OR “meat free” OR “alternative protein*” OR meatless OR “meat reduction” OR “reduce meat*” OR “reduced meat*” ) OR SU ( “plant-rich” OR “plant rich” OR “plant-based” OR “plant based” OR “plant alternative*” OR “plant-forward” OR “plant forward” OR “plant protein*” OR “plant derived” OR “non-animal protein*” OR vegetable* OR fruit* OR grain* OR soy OR nut OR nuts OR seed* OR tofu OR tempeh OR bean* OR legume* OR vegan* OR vegetarian* OR flexitarian* OR “meat-free” OR “meat free” OR “alternative protein*” OR meatless OR “meat reduction” OR “reduce meat*” OR “reduced meat*” )) AND (TI ( policy OR policies OR guideline* OR program* OR commitment* OR initiative* OR campaign* OR standard* OR recommendation* OR intervention* OR nudg* OR “choice architecture*” OR “behavioral economics” OR “default choice*” OR “nutrition profile*” OR “nutrient profile*” OR “nutritional profile*” OR portion* OR promot* OR proximity OR prompting OR pric* OR cost* OR placement OR label* OR “point of purchase” OR “point-of-purchase” OR marketing OR “marketing-mix” OR advertis* ) OR AB ( policy OR policies OR guideline* OR program* OR commitment* OR initiative* OR campaign* OR standard* OR recommendation* OR intervention* OR nudg* OR “choice architecture*” OR “behavioral economics” OR “default choice*” OR “nutrition profile*” OR “nutrient profile*” OR “nutritional profile*” OR portion* OR promot* OR proximity OR prompting OR pric* OR cost* OR placement OR label* OR “point of purchase” OR “point-of-purchase” OR marketing OR “marketing-mix” OR advertis* ) OR SU ( policy OR policies OR guideline* OR program* OR | | |

| | | | | | |
|---|---|---|---|---|---|
| | | | commitment* OR initiative* OR campaign* OR standard* OR recommendation* OR intervention* OR nudg* OR “choice architecture*” OR “behavioral economics” OR “default choice*” OR “nutrition profile*” OR “nutrient profile*” OR “nutritional profile*” OR portion* OR promot* OR proximity OR prompting OR pric* OR cost* OR placement OR label* OR “point of purchase” OR “point-of-purchase” OR marketing OR “marketing-mix” OR advertis* ) OR DE "Guidelines" OR DE "Policy" OR DE "Marketing") | | |
| Business Source Complete | English; 01/01/2010-11/15/2024 (present date of search)<br><br>Title, Abstract or Author-Supplied Abstract, or Author-Supplied Keywords | EBSCOhost | (TI ( university OR universities OR undergrad OR undergrads OR undergraduate OR undergraduates OR college OR colleges OR collegiate OR “higher education” OR campus* OR “post-secondary” OR postsecondary OR “post secondary” OR “Post-graduate” OR “Post-graduates” OR “post graduate” OR “post graduates” OR “tertiary education” ) OR AB ( university OR universities OR undergrad OR undergrads OR undergraduate OR undergraduates OR college OR colleges OR collegiate OR “higher education” OR campus* OR “post-secondary” OR postsecondary OR “post secondary” OR “Post-graduate” OR “Post-graduates” OR “post graduate” OR “post graduates” OR “tertiary education” ) OR KW ( university OR universities OR undergrad OR undergrads OR undergraduate OR undergraduates OR college OR colleges OR collegiate OR “higher education” OR campus* OR “post-secondary” OR postsecondary OR “post secondary” OR “Post-graduate” OR “Post-graduates” OR “post graduate” OR “post graduates” OR “tertiary education” ) OR DE "UNIVERSITIES & colleges" ) AND (TI ( food* OR cater* OR eatery OR eateries OR nutrition OR diet* OR meal* OR dining OR canteen* OR menu* OR cafe* OR lunch* OR breakfast* OR dinner* OR “snack bar*” OR grill* OR restaurant* ) OR AB ( food* OR cater* OR eatery OR eateries OR nutrition OR diet* OR meal* OR dining OR canteen* OR menu* OR cafe* OR lunch* OR breakfast* OR dinner* OR “snack bar*” OR grill* OR restaurant* ) OR KW ( food* OR cater* OR eatery OR eateries OR nutrition OR diet* OR meal* OR dining OR canteen* OR menu* OR cafe* OR lunch* OR breakfast* OR dinner* OR “snack bar*” OR grill* OR restaurant* ) OR DE "FOOD service") AND (TI ( “plant-rich” OR “plant rich” OR “plant-based” OR “plant based” OR “plant alternative*” OR “plant-forward” OR “plant forward” OR “plant protein*” OR “plant derived” OR “non-animal protein*” OR vegetable* OR fruit* OR grain* OR soy OR nut OR nuts OR seed* OR tofu OR tempeh OR bean* OR legume* OR vegan* OR vegetarian* OR flexitarian* OR “meat-free” OR “meat free” OR “alternative protein*” OR meatless OR “meat reduction” OR “reduce meat*” OR “reduced meat*” ) OR AB ( “plant-rich” OR “plant rich” OR “plant-based” OR “plant based” OR “plant alternative*” OR “plant- | 11/15/2024 | 217 |

| | | | | | |
|---|---|---|---|---|---|
| | | | forward" OR "plant forward" OR "plant protein*" OR "plant derived" OR "non-animal protein*" OR vegetable* OR fruit* OR grain* OR soy OR nut OR nuts OR seed* OR tofu OR tempeh OR bean* OR legume* OR vegan* OR vegetarian* OR flexitarian* OR "meat-free" OR "meat free" OR "alternative protein*" OR meatless OR "meat reduction" OR "reduce meat*" OR "reduced meat*" ) OR KW ( "plant-rich" OR "plant rich" OR "plant-based" OR "plant based" OR "plant alternative*" OR "plant-forward" OR "plant forward" OR "plant protein*" OR "plant derived" OR "non-animal protein*" OR vegetable* OR fruit* OR grain* OR soy OR nut OR nuts OR seed* OR tofu OR tempeh OR bean* OR legume* OR vegan* OR vegetarian* OR flexitarian* OR "meat-free" OR "meat free" OR "alternative protein*" OR meatless OR "meat reduction" OR "reduce meat*" OR "reduced meat*" )) AND (TI ( policy OR policies OR guideline* OR program* OR commitment* OR initiative* OR campaign* OR standard* OR recommendation* OR intervention* OR nudg* OR "choice architecture*" OR "behavioral economics" OR "default choice*" OR "nutrition profile*" OR "nutrient profile*" OR "nutritional profile*" OR portion* OR promot* OR proximity OR prompting OR pric* OR cost* OR placement OR label* OR "point of purchase" OR "point-of-purchase" OR marketing OR "marketing-mix" OR advertis* ) OR AB ( policy OR policies OR guideline* OR program* OR commitment* OR initiative* OR campaign* OR standard* OR recommendation* OR intervention* OR nudg* OR "choice architecture*" OR "behavioral economics" OR "default choice*" OR "nutrition profile*" OR "nutrient profile*" OR "nutritional profile*" OR portion* OR promot* OR proximity OR prompting OR pric* OR cost* OR placement OR label* OR "point of purchase" OR "point-of-purchase" OR marketing OR "marketing-mix" OR advertis* ) OR KW ( policy OR policies OR guideline* OR program* OR commitment* OR initiative* OR campaign* OR standard* OR recommendation* OR intervention* OR nudg* OR "choice architecture*" OR "behavioral economics" OR "default choice*" OR "nutrition profile*" OR "nutrient profile*" OR "nutritional profile*" OR portion* OR promot* OR proximity OR prompting OR pric* OR cost* OR placement OR label* OR "point of purchase" OR "point-of-purchase" OR marketing OR "marketing-mix" OR advertis* ) OR DE "MARKETING" OR DE "MARKETING mix" OR DE "ADVERTISING" OR DE "PRODUCT placement" ) | | |
| ProQuest One Business | English; 01/01/2010-11/15/2024 | ProQuest | ((MAINSUBJECT.EXACT("Colleges & universities") OR MAINSUBJECT.EXACT("Higher education")) OR title(university OR universities OR undergrad OR undergrads OR undergraduate OR undergraduates OR college OR colleges OR collegiate OR "higher education" OR campus* OR "post-secondary" OR | 11/15/24 | 1,162 |

| | | | | | |
|---|---|---|---|---|---|
| | (present date of search)<br><br>Title/abstract | | postsecondary OR "post secondary" OR "Post-graduate" OR "Post-graduates" OR "post graduate" OR "post graduates" OR "tertiary education") OR abstract(university OR universities OR undergrad OR undergrads OR undergraduate OR undergraduates OR college OR colleges OR collegiate OR "higher education" OR campus* OR "post-secondary" OR postsecondary OR "post secondary" OR "Post-graduate" OR "Post-graduates" OR "post graduate" OR "post graduates" OR "tertiary education")) AND (((title("plant-rich" OR "plant rich" OR "plant-based" OR "plant based" OR "plant alternative*" OR "plant-forward" OR "plant forward" OR "plant protein*" OR "plant derived" OR "non-animal protein*" OR vegetable* OR fruit* OR grain* OR soy OR nut OR nuts OR seed* OR tofu OR tempeh OR bean* OR legume* OR vegan* OR vegetarian* OR flexitarian* OR "meat-free" OR "meat free" OR "alternative protein*" OR meatless OR "meat reduction" OR "reduce meat*" OR "reduced meat*") OR abstract("plant-rich" OR "plant rich" OR "plant-based" OR "plant based" OR "plant alternative*" OR "plant-forward" OR "plant forward" OR "plant protein*" OR "plant derived" OR "non-animal protein*" OR vegetable* OR fruit* OR grain* OR soy OR nut OR nuts OR seed* OR tofu OR tempeh OR bean* OR legume* OR vegan* OR vegetarian* OR flexitarian* OR "meat-free" OR "meat free" OR "alternative protein*" OR meatless OR "meat reduction" OR "reduce meat*" OR "reduced meat*")) AND ((MAINSUBJECT.EXACT("Food service") OR MAINSUBJECT.EXACT("Food")) OR title(food* OR cater* OR eatery OR eateries OR nutrition OR diet* OR meal* OR dining OR canteen* OR menu* OR cafe* OR lunch* OR breakfast* OR dinner* OR "snack bar*" OR grill* OR restaurant*) OR abstract(food* OR cater* OR eatery OR eateries OR nutrition OR diet* OR meal* OR dining OR canteen* OR menu* OR cafe* OR lunch* OR breakfast* OR dinner* OR "snack bar*" OR grill* OR restaurant*))) OR (MAINSUBJECT.EXACT("Vegetarianism") OR MAINSUBJECT.EXACT("Plant-based foods") OR MAINSUBJECT.EXACT("Veganism"))) AND ((MAINSUBJECT.EXACT("Marketing") OR MAINSUBJECT.EXACT("Advertising") OR MAINSUBJECT.EXACT("Guidelines") OR MAINSUBJECT.EXACT("Marketing mixes")) OR title(policy OR policies OR guideline* OR program* OR commitment* OR initiative* OR campaign* OR standard* OR recommendation* OR intervention* OR nudg* OR "choice architecture*" OR "behavioral economics" OR "default choice*" OR "nutrition profile*" OR "nutrient profile*" OR "nutritional profile*" OR portion* OR promot* OR proximity OR prompting OR pric* OR cost* OR placement OR label* OR "point of purchase" OR "point-of-purchase" OR marketing OR | | |

| | | | | | |
|---|---|---|---|---|---|
| | | | "marketing-mix" OR advertis*) OR abstract(policy OR policies OR guideline* OR program* OR commitment* OR initiative* OR campaign* OR standard* OR recommendation* OR intervention* OR nudg* OR "choice architecture*" OR "behavioral economics" OR "default choice*" OR "nutrition profile*" OR "nutrient profile*" OR "nutritional profile*" OR portion* OR promot* OR proximity OR prompting OR pric* OR cost* OR placement OR label* OR "point of purchase" OR "point-of-purchase" OR marketing OR "marketing-mix" OR advertis*)) | | |
| Core Collection | English; 01/01/2010-11/15/2024 (present date of search)<br><br>Topic (searches title, abstract, keyword plus, and author keywords) | Web of Science | TS=(university OR universities OR undergrad OR undergrads OR undergraduate OR undergraduates OR college OR colleges OR collegiate OR “higher education” OR campus* OR “post-secondary” OR postsecondary OR “post secondary” OR “Post-graduate” OR “Post-graduates” OR “post graduate” OR “post graduates” OR “tertiary education”) AND TS=(food* OR cater* OR eatery OR eateries OR nutrition OR diet* OR meal* OR dining OR canteen* OR menu* OR cafe* OR lunch* OR breakfast* OR dinner* OR “snack bar*” OR grill* OR restaurant*) AND TS=(“plant-rich” OR “plant rich” OR “plant-based” OR “plant based” OR “plant alternative*” OR “plant-forward” OR “plant forward” OR “plant protein*” OR “plant derived” OR “non-animal protein*” OR vegetable* OR fruit* OR grain* OR soy OR nut OR nuts OR seed* OR tofu OR tempeh OR bean* OR legume* OR vegan* OR vegetarian* OR flexitarian* OR “meat-free” OR “meat free” OR “alternative protein*” OR meatless OR “meat reduction” OR “reduce meat*” OR “reduced meat*”) AND TS=(policy OR policies OR guideline* OR program* OR commitment* OR initiative* OR campaign* OR standard* OR recommendation* OR intervention* OR nudg* OR “choice architecture*” OR “behavioral economics” OR “default choice*” OR “nutrition profile*” OR “nutrient profile*” OR “nutritional profile*” OR portion* OR promot* OR proximity OR prompting OR pric* OR cost* OR placement OR label* OR “point of purchase” OR “point-of-purchase” OR marketing OR “marketing-mix” OR advertis*) | 11/15/24 | 2,837 |
| Access World News | North America; USA; English; 01/01/2010-11/15/2024 (present date of search)<br><br>Field: Headline AND All Text | NewsBank | university OR universities OR college OR colleges OR campus [Headline] AND<br>“dining services” OR food OR nutrition OR catering OR "plant-rich" OR "plant-forward" OR "plant-based" OR vegetarian OR vegan OR "meat-free" OR meatless OR commitment OR policy OR program OR practices OR marketing OR nudge OR “choice architecture” [All-text] | 11/15/24 | First 300 results (out of 1,880,087) |

| | | | | | |
|---|---|---|---|---|---|
| | Sort by: Best Match | | | | |
| Google Scholar | Since 2010 – 2024 (12/31/2024; present date of search)<br><br>Search strategy built using advanced search feature; keywords appear anywhere in the article; sorted by relevance<br><br>Searched in incognito mode | | university dining food OR nutrition OR catering OR "plant-rich" OR "plant-forward" OR "plant-based" OR vegetarian OR vegan OR "meat-free" OR meatless OR commitment OR policy OR program OR practices OR marketing OR nudge OR "choice architecture"<br><br>university dining food OR nutrition OR catering OR "plant-rich" OR "plant-forward" OR "plant-based" OR vegetarian OR vegan OR "meat-free" OR meatless OR commitment OR policy OR program OR practices OR marketing OR nudge OR "choice architecture" | 12/31/24 | First 300 results (out of ~149,000) |
| | | | | | Total prior to deduplication: 6504 |
| Follow-up Search | | | | | |
| PubMed | English; 11/15/2024-12/31/2024 (present date of search)<br><br>Title/Abstract | National Library of Medicine | ((("Universities"[Mesh]) OR (university[Title/Abstract] OR universities[Title/Abstract] OR undergrad[Title/Abstract] OR undergrads[Title/Abstract] OR undergraduate[Title/Abstract] OR undergraduates[Title/Abstract] OR college[Title/Abstract] OR colleges[Title/Abstract] OR collegiate[Title/Abstract] OR "higher education"[Title/Abstract] OR campus*[Title/Abstract] OR "post-secondary"[Title/Abstract] OR postsecondary[Title/Abstract] OR "post secondary"[Title/Abstract] OR "Post-graduate"[Title/Abstract] OR "Post-graduates"[Title/Abstract] OR "post graduate"[Title/Abstract] OR "post | 12/31/2024 | 26 |

| | | | | | |
|---|---|---|---|---|---|
| | | | graduates"[Title/Abstract] OR "tertiary education"[Title/Abstract])) AND ((((food*[Title/Abstract] OR cater*[Title/Abstract] OR eatery[Title/Abstract] OR eateries[Title/Abstract] OR nutrition[Title/Abstract] OR diet*[Title/Abstract] OR meal*[Title/Abstract] OR dining[Title/Abstract] OR canteen*[Title/Abstract] OR menu*[Title/Abstract] OR cafe*[Title/Abstract] OR lunch*[Title/Abstract] OR breakfast*[Title/Abstract] OR dinner*[Title/Abstract] OR "snack bar*"[Title/Abstract] OR grill*[Title/Abstract] OR restaurant*[Title/Abstract]) OR ("Food"[Mesh] OR "Food Services"[Mesh])) AND ("plant-rich"[Title/Abstract] OR "plant rich"[Title/Abstract] OR "plant-based"[Title/Abstract] OR "plant based"[Title/Abstract] OR "plant alternative*"[Title/Abstract] OR "plant-forward"[Title/Abstract] OR "plant forward"[Title/Abstract] OR "plant protein*"[Title/Abstract] OR "plant derived"[Title/Abstract] OR "non-animal protein*"[Title/Abstract] OR vegetable*[Title/Abstract] OR fruit*[Title/Abstract] OR grain*[Title/Abstract] OR soy[Title/Abstract] OR nut[Title/Abstract] OR nuts[Title/Abstract] OR seed*[Title/Abstract] OR tofu[Title/Abstract] OR tempeh[Title/Abstract] OR bean*[Title/Abstract] OR legume*[Title/Abstract] OR vegan*[Title/Abstract] OR vegetarian*[Title/Abstract] OR flexitarian*[Title/Abstract] OR "meat-free"[Title/Abstract] OR "meat free"[Title/Abstract] OR "alternative protein*"[Title/Abstract] OR meatless[Title/Abstract] OR "meat reduction"[Title/Abstract] OR "reduce meat*"[Title/Abstract] OR "reduced meat*"[Title/Abstract])) OR ("Diet, Vegan"[Mesh] OR "Diet, Vegetarian"[Mesh] OR "Meat Substitutes"[Mesh] OR "Diet, Plant-Based"[Mesh]))) AND (("Policy"[Mesh] OR "Organizational Policy"[Mesh] OR "Marketing"[Mesh] OR "Advertising"[Mesh]) OR (policy[Title/Abstract] OR policies[Title/Abstract] OR guideline*[Title/Abstract] OR program*[Title/Abstract] OR commitment*[Title/Abstract] OR initiative*[Title/Abstract] OR campaign*[Title/Abstract] OR standard*[Title/Abstract] OR recommendation*[Title/Abstract] OR intervention*[Title/Abstract] OR nudg*[Title/Abstract] OR "choice architecture*"[Title/Abstract] OR "behavioral economics"[Title/Abstract] OR "default choice*"[Title/Abstract] OR "nutrition profile*"[Title/Abstract] OR "nutrient profile*"[Title/Abstract] OR "nutritional profile*"[Title/Abstract] OR portion*[Title/Abstract] OR promot*[Title/Abstract] OR proximity[Title/Abstract] OR prompting[Title/Abstract] OR pric*[Title/Abstract] OR cost*[Title/Abstract] OR placement[Title/Abstract] OR label*[Title/Abstract] OR "point of purchase"[Title/Abstract] OR "point-of-purchase"[Title/Abstract] OR | | |

| | | | | | |
|---|---|---|---|---|---|
| | | | marketing[Title/Abstract] OR "marketing-mix"[Title/Abstract] OR advertis*[Title/Abstract])) | | |
| ERIC | English; 11/01/2024-12/31/2024 (present date of search)<br><br>Title/Abstract/Subject | EBSCOhost | (TI ( university OR universities OR undergrad OR undergrads OR undergraduate OR undergraduates OR college OR colleges OR collegiate OR “higher education” OR campus* OR “post-secondary” OR postsecondary OR “post secondary” OR “Post-graduate” OR “Post-graduates” OR “post graduate” OR “post graduates” OR “tertiary education” ) OR AB ( university OR universities OR undergrad OR undergrads OR undergraduate OR undergraduates OR college OR colleges OR collegiate OR “higher education” OR campus* OR “post-secondary” OR postsecondary OR “post secondary” OR “Post-graduate” OR “Post-graduates” OR “post graduate” OR “post graduates” OR “tertiary education” ) OR SU ( university OR universities OR undergrad OR undergrads OR undergraduate OR undergraduates OR college OR colleges OR collegiate OR “higher education” OR campus* OR “post-secondary” OR postsecondary OR “post secondary” OR “Post-graduate” OR “Post-graduates” OR “post graduate” OR “post graduates” OR “tertiary education” ) OR DE "Colleges" OR DE "College Environment") AND (TI ( food* OR cater* OR eatery OR eateries OR nutrition OR diet* OR meal* OR dining OR canteen* OR menu* OR cafe* OR lunch* OR breakfast* OR dinner* OR “snack bar*” OR grill* OR restaurant* ) OR AB ( food* OR cater* OR eatery OR eateries OR nutrition OR diet* OR meal* OR dining OR canteen* OR menu* OR cafe* OR lunch* OR breakfast* OR dinner* OR “snack bar*” OR grill* OR restaurant* ) OR SU ( food* OR cater* OR eatery OR eateries OR nutrition OR diet* OR meal* OR dining OR canteen* OR menu* OR cafe* OR lunch* OR breakfast* OR dinner* OR “snack bar*” OR grill* OR restaurant* ) OR DE "Food" OR DE "Food Service" OR DE "Dining Facilities")) AND (TI ( “plant-rich” OR “plant rich” OR “plant-based” OR “plant based” OR “plant alternative*” OR “plant-forward” OR “plant forward” OR “plant protein*” OR “plant derived” OR “non-animal protein*” OR vegetable* OR fruit* OR grain* OR soy OR nut OR nuts OR seed* OR tofu OR tempeh OR bean* OR legume* OR vegan* OR vegetarian* OR flexitarian* OR “meat-free” OR “meat free” OR “alternative protein*” OR meatless OR “meat reduction” OR “reduce meat*” OR “reduced meat*” ) OR AB ( “plant-rich” OR “plant rich” OR “plant-based” OR “plant based” OR “plant alternative*” OR “plant-forward” OR “plant forward” OR “plant protein*” OR “plant derived” OR “non-animal protein*” OR vegetable* OR fruit* OR grain* OR soy OR nut OR nuts OR seed* OR tofu OR tempeh OR bean* OR legume* OR vegan* OR vegetarian* OR flexitarian* OR “meat-free” OR “meat free” | 12/31/2024 | 0 |

| | | | | | |
|---|---|---|---|---|---|
| | | | OR “alternative protein*” OR meatless OR “meat reduction” OR “reduce meat*” OR “reduced meat*” ) OR SU ( “plant-rich” OR “plant rich” OR “plant-based” OR “plant based” OR “plant alternative*” OR “plant-forward” OR “plant forward” OR “plant protein*” OR “plant derived” OR “non-animal protein*” OR vegetable* OR fruit* OR grain* OR soy OR nut OR nuts OR seed* OR tofu OR tempeh OR bean* OR legume* OR vegan* OR vegetarian* OR flexitarian* OR “meat-free” OR “meat free” OR “alternative protein*” OR meatless OR “meat reduction” OR “reduce meat*” OR “reduced meat*” )) AND (TI ( policy OR policies OR guideline* OR program* OR commitment* OR initiative* OR campaign* OR standard* OR recommendation* OR intervention* OR nudg* OR “choice architecture*” OR “behavioral economics” OR “default choice*” OR “nutrition profile*” OR “nutrient profile*” OR “nutritional profile*” OR portion* OR promot* OR proximity OR prompting OR pric* OR cost* OR placement OR label* OR “point of purchase” OR “point-of-purchase” OR marketing OR “marketing-mix” OR advertis* ) OR AB ( policy OR policies OR guideline* OR program* OR commitment* OR initiative* OR campaign* OR standard* OR recommendation* OR intervention* OR nudg* OR “choice architecture*” OR “behavioral economics” OR “default choice*” OR “nutrition profile*” OR “nutrient profile*” OR “nutritional profile*” OR portion* OR promot* OR proximity OR prompting OR pric* OR cost* OR placement OR label* OR “point of purchase” OR “point-of-purchase” OR marketing OR “marketing-mix” OR advertis* ) OR SU ( policy OR policies OR guideline* OR program* OR commitment* OR initiative* OR campaign* OR standard* OR recommendation* OR intervention* OR nudg* OR “choice architecture*” OR “behavioral economics” OR “default choice*” OR “nutrition profile*” OR “nutrient profile*” OR “nutritional profile*” OR portion* OR promot* OR proximity OR prompting OR pric* OR cost* OR placement OR label* OR “point of purchase” OR “point-of-purchase” OR marketing OR “marketing-mix” OR advertis* ) OR DE "Guidelines" OR DE "Policy" OR DE "Marketing") | | |
| Business Source Complete | English; 11/01/2024-12/31/2024 (present date of search)<br><br>Title, Abstract or Author- | EBSCOhost | (TI ( university OR universities OR undergrad OR undergrads OR undergraduate OR undergraduates OR college OR colleges OR collegiate OR “higher education” OR campus* OR “post-secondary” OR postsecondary OR “post secondary” OR “Post-graduate” OR “Post-graduates” OR “post graduate” OR “post graduates” OR “tertiary education” ) OR AB ( university OR universities OR undergrad OR undergrads OR undergraduate OR undergraduates OR college OR colleges OR collegiate OR “higher education” OR campus* OR “post-secondary” OR postsecondary OR “post secondary” OR “Post-graduate” OR “Post-graduates” OR “post graduate” OR “post | 12/31/2024 | 4 |

| | | | | | |
|---|---|---|---|---|---|
| | Supplied Abstract, or Author-Supplied Keywords | | graduates” OR “tertiary education” ) OR KW ( university OR universities OR undergrad OR undergrads OR undergraduate OR undergraduates OR college OR colleges OR collegiate OR “higher education” OR campus* OR “post-secondary” OR postsecondary OR “post secondary” OR “Post-graduate” OR “Post-graduates” OR “post graduate” OR “post graduates” OR “tertiary education” ) OR DE "UNIVERSITIES & colleges" ) AND (TI ( food* OR cater* OR eatery OR eateries OR nutrition OR diet* OR meal* OR dining OR canteen* OR menu* OR cafe* OR lunch* OR breakfast* OR dinner* OR “snack bar*” OR grill* OR restaurant* ) OR AB ( food* OR cater* OR eatery OR eateries OR nutrition OR diet* OR meal* OR dining OR canteen* OR menu* OR cafe* OR lunch* OR breakfast* OR dinner* OR “snack bar*” OR grill* OR restaurant* ) OR KW ( food* OR cater* OR eatery OR eateries OR nutrition OR diet* OR meal* OR dining OR canteen* OR menu* OR cafe* OR lunch* OR breakfast* OR dinner* OR “snack bar*” OR grill* OR restaurant* ) OR DE "FOOD service") AND (TI ( “plant-rich” OR “plant rich” OR “plant-based” OR “plant based” OR “plant alternative*” OR “plant-forward” OR “plant forward” OR “plant protein*” OR “plant derived” OR “non-animal protein*” OR vegetable* OR fruit* OR grain* OR soy OR nut OR nuts OR seed* OR tofu OR tempeh OR bean* OR legume* OR vegan* OR vegetarian* OR flexitarian* OR “meat-free” OR “meat free” OR “alternative protein*” OR meatless OR “meat reduction” OR “reduce meat*” OR “reduced meat*” ) OR AB ( “plant-rich” OR “plant rich” OR “plant-based” OR “plant based” OR “plant alternative*” OR “plant-forward” OR “plant forward” OR “plant protein*” OR “plant derived” OR “non-animal protein*” OR vegetable* OR fruit* OR grain* OR soy OR nut OR nuts OR seed* OR tofu OR tempeh OR bean* OR legume* OR vegan* OR vegetarian* OR flexitarian* OR “meat-free” OR “meat free” OR “alternative protein*” OR meatless OR “meat reduction” OR “reduce meat*” OR “reduced meat*” ) OR KW ( “plant-rich” OR “plant rich” OR “plant-based” OR “plant based” OR “plant alternative*” OR “plant-forward” OR “plant forward” OR “plant protein*” OR “plant derived” OR “non-animal protein*” OR vegetable* OR fruit* OR grain* OR soy OR nut OR nuts OR seed* OR tofu OR tempeh OR bean* OR legume* OR vegan* OR vegetarian* OR flexitarian* OR “meat-free” OR “meat free” OR “alternative protein*” OR meatless OR “meat reduction” OR “reduce meat*” OR “reduced meat*” )) AND (TI ( policy OR policies OR guideline* OR program* OR commitment* OR initiative* OR campaign* OR standard* OR recommendation* OR intervention* OR nudg* OR “choice architecture*” OR “behavioral economics” OR “default choice*” OR “nutrition profile*” OR “nutrient profile*” OR “nutritional profile*” OR portion* OR | | |

| | | | | | |
|---|---|---|---|---|---|
| | | | promot* OR proximity OR prompting OR pric* OR cost* OR placement OR label* OR “point of purchase” OR “point-of-purchase” OR marketing OR “marketing-mix” OR advertis* ) OR AB ( policy OR policies OR guideline* OR program* OR commitment* OR initiative* OR campaign* OR standard* OR recommendation* OR intervention* OR nudg* OR “choice architecture*” OR “behavioral economics” OR “default choice*” OR “nutrition profile*” OR “nutrient profile*” OR “nutritional profile*” OR portion* OR promot* OR proximity OR prompting OR pric* OR cost* OR placement OR label* OR “point of purchase” OR “point-of-purchase” OR marketing OR “marketing-mix” OR advertis* ) OR KW ( policy OR policies OR guideline* OR program* OR commitment* OR initiative* OR campaign* OR standard* OR recommendation* OR intervention* OR nudg* OR “choice architecture*” OR “behavioral economics” OR “default choice*” OR “nutrition profile*” OR “nutrient profile*” OR “nutritional profile*” OR portion* OR promot* OR proximity OR prompting OR pric* OR cost* OR placement OR label* OR “point of purchase” OR “point-of-purchase” OR marketing OR “marketing-mix” OR advertis* ) OR DE "MARKETING" OR DE "MARKETING mix" OR DE "ADVERTISING" OR DE "PRODUCT placement" ) | | |
| ProQuest One Business | English; 11/15/2024-12/31/2024 (present date of search)<br><br>Title/abstract | ProQuest | ((MAINSUBJECT.EXACT("Colleges & universities") OR MAINSUBJECT.EXACT("Higher education")) OR title(university OR universities OR undergrad OR undergrads OR undergraduate OR undergraduates OR college OR colleges OR collegiate OR "higher education" OR campus* OR "post-secondary" OR postsecondary OR "post secondary" OR "Post-graduate" OR "Post-graduates" OR "post graduate" OR "post graduates" OR "tertiary education") OR abstract(university OR universities OR undergrad OR undergrads OR undergraduate OR undergraduates OR college OR colleges OR collegiate OR "higher education" OR campus* OR "post-secondary" OR postsecondary OR "post secondary" OR "Post-graduate" OR "Post-graduates" OR "post graduate" OR "post graduates" OR "tertiary education")) AND (((title("plant-rich" OR "plant rich" OR "plant-based" OR "plant based" OR "plant alternative*" OR "plant-forward" OR "plant forward" OR "plant protein*" OR "plant derived" OR "non-animal protein*" OR vegetable* OR fruit* OR grain* OR soy OR nut OR nuts OR seed* OR tofu OR tempeh OR bean* OR legume* OR vegan* OR vegetarian* OR flexitarian* OR "meat-free" OR "meat free" OR "alternative protein*" OR meatless OR "meat reduction" OR "reduce meat*" OR "reduced meat*") OR abstract("plant-rich" OR "plant rich" OR "plant-based" OR "plant based" OR "plant alternative*" OR "plant-forward" OR "plant forward" OR "plant protein*" OR "plant derived" OR "non-animal protein*" OR vegetable* OR fruit* OR grain* | 12/31/2024 | 3 |

| | | | | | |
|---|---|---|---|---|---|
| | | | OR soy OR nut OR nuts OR seed* OR tofu OR tempeh OR bean* OR legume* OR vegan* OR vegetarian* OR flexitarian* OR "meat-free" OR "meat free" OR "alternative protein*" OR meatless OR "meat reduction" OR "reduce meat*" OR "reduced meat*")) AND ((MAINSUBJECT.EXACT("Food service") OR MAINSUBJECT.EXACT("Food")) OR title(food* OR cater* OR eatery OR eateries OR nutrition OR diet* OR meal* OR dining OR canteen* OR menu* OR cafe* OR lunch* OR breakfast* OR dinner* OR "snack bar*" OR grill* OR restaurant*) OR abstract(food* OR cater* OR eatery OR eateries OR nutrition OR diet* OR meal* OR dining OR canteen* OR menu* OR cafe* OR lunch* OR breakfast* OR dinner* OR "snack bar*" OR grill* OR restaurant*))) OR (MAINSUBJECT.EXACT("Vegetarianism") OR MAINSUBJECT.EXACT("Plant-based foods") OR MAINSUBJECT.EXACT("Veganism"))) AND ((MAINSUBJECT.EXACT("Marketing") OR MAINSUBJECT.EXACT("Advertising") OR MAINSUBJECT.EXACT("Guidelines") OR MAINSUBJECT.EXACT("Marketing mixes")) OR title(policy OR policies OR guideline* OR program* OR commitment* OR initiative* OR campaign* OR standard* OR recommendation* OR intervention* OR nudg* OR "choice architecture*" OR "behavioral economics" OR "default choice*" OR "nutrition profile*" OR "nutrient profile*" OR "nutritional profile*" OR portion* OR promot* OR proximity OR prompting OR pric* OR cost* OR placement OR label* OR "point of purchase" OR "point-of-purchase" OR marketing OR "marketing-mix" OR advertis*) OR abstract(policy OR policies OR guideline* OR program* OR commitment* OR initiative* OR campaign* OR standard* OR recommendation* OR intervention* OR nudg* OR "choice architecture*" OR "behavioral economics" OR "default choice*" OR "nutrition profile*" OR "nutrient profile*" OR "nutritional profile*" OR portion* OR promot* OR proximity OR prompting OR pric* OR cost* OR placement OR label* OR "point of purchase" OR "point-of-purchase" OR marketing OR "marketing-mix" OR advertis*)) | | |
| Core Collection | English; 11/15/2024-12/31/2024 (present date of search)<br><br>Topic (searches title, abstract, | Web of Science | TS=(university OR universities OR undergrad OR undergrads OR undergraduate OR undergraduates OR college OR colleges OR collegiate OR “higher education” OR campus* OR “post-secondary” OR postsecondary OR “post secondary” OR “Post-graduate” OR “Post-graduates” OR “post graduate” OR “post graduates” OR “tertiary education”) AND TS=(food* OR cater* OR eatery OR eateries OR nutrition OR diet* OR meal* OR dining OR canteen* OR menu* OR cafe* OR lunch* OR breakfast* OR dinner* OR “snack bar*” OR grill* OR restaurant*) AND TS=(“plant-rich” OR “plant rich” OR “plant-based” OR “plant based” OR “plant alternative*” OR “plant- | 12/31/2024 | 22 |

| | | | | | |
|---|---|---|---|---|---|
| | keyword plus, and author keywords) | | forward" OR "plant forward" OR "plant protein*" OR "plant derived" OR "non-animal protein*" OR vegetable* OR fruit* OR grain* OR soy OR nut OR nuts OR seed* OR tofu OR tempeh OR bean* OR legume* OR vegan* OR vegetarian* OR flexitarian* OR "meat-free" OR "meat free" OR "alternative protein*" OR meatless OR "meat reduction" OR "reduce meat*" OR "reduced meat*") AND TS=(policy OR policies OR guideline* OR program* OR commitment* OR initiative* OR campaign* OR standard* OR recommendation* OR intervention* OR nudg* OR "choice architecture*" OR "behavioral economics" OR "default choice*" OR "nutrition profile*" OR "nutrient profile*" OR "nutritional profile*" OR portion* OR promot* OR proximity OR prompting OR pric* OR cost* OR placement OR label* OR "point of purchase" OR "point-of-purchase" OR marketing OR "marketing-mix" OR advertis*) | | |
| Access World News | North America; USA; English; 11/15/2024-12/31/2024 (present date of search)<br><br>Field: Headline AND All Text<br><br>Sort by: Best Match | NewsBank | university OR universities OR college OR colleges OR campus [Headline] AND<br>"dining services" OR food OR nutrition OR catering OR "plant-rich" OR "plant-forward" OR "plant-based" OR vegetarian OR vegan OR "meat-free" OR meatless OR commitment OR policy OR program OR practices OR marketing OR nudge OR "choice architecture" [All-text] | 12/31/2024 | First 100 results (out of 13,218) |
| | | | | | Total added prior to deduplication: 155 |

**Abbreviations and acronyms:** Education Resources Information Center (ERIC)

**Supplemental Table 4.** Supplemental Search of Organizations and Higher Education Sustainability Ranking Systems, Commitments, or Programs Related to Plant-rich Menu Options

| | **Higher education sustainability ranking systems, commitments, or programs related to plant-rich menu options** | | | |
|---|---|---|---|---|
| Organization | Date(s) searched and/or reviewed | Search String and/or filters | Evidence Source | **Number of Results** |
| **Humane World for Animals website** | 5/8/2025-8/5/2025 | ("University" OR "college") | Humane World for Animals (n.d.) | 34 |
| **The Coolfood Pledge** | 5/8/2025-5/12/2025 | None applied | Coolfood (n.d.) | 1 |
| **Forward Food Pledge** | 5/8/2025-5/12/2025 | None applied | Forward Food (2020) | 1 |
| **The Menus of Change University Research Collaborative** | 5/13/2025-5/15/2025 | The Operational Research Publication website and corresponding Plant-Forward Diet Promotion records | The Menus of Change University Research Collaborative (n.d.) | 8 |
| **Meatless Monday website** | 6/12/2025 | Filtered by case studies and success stories | Johns Hopkins Center for a Livable Future and Meatless Monday (n.d.) | 23 |
| **AASHE STARS report among previously identified higher education institutions actively participating in AASHE STARS** | 6/17/2025-7/3/2025 | None applied | Association for the Advancement of Sustainability in Higher Education (n.d.) | 186 |

*Humane World for Animals, College and University Protein Sustainability Scorecard and AASHE STARS reports were included due to its description of higher education dining services efforts implemented prior to 2025

**Abbreviations and acronyms:** Association for the Advancement of Sustainability in Higher Education (AASHE); Sustainability Tracking, Assessment & Rating System (STARS)

**Supplemental Table 5.** Supplemental Search of Higher Education Websites

| **Targeted *higher education websites** | | | |
|---|---|---|---|
| | Search String | Date(s) searched and/or reviewed | **Number of Relevant and Included Results |
| **Google search engine (incognito mode)** | "higher education name" AND "sustainable OR dining"<br><br>"higher education name" AND "program, policy, or commitment name" if applicable | 3/21/2025-8/20/2025 | 125 |

*Websites with the copyright date of 2025 were included if the websites described the active implementation of MMCA strategies
**Due to the high number of Google results, only the total number of relevant and included results was documented
