# Supplementary material for: Higher education institutions and the use of marketing-mix choice architecture strategies to encourage plant-rich menu options and sustainable dietary patterns: a scoping review
Source: Front Nutr. 2026 Mar 24;13:1774451. doi: 10.3389/fnut.2026.1774451 (PMC13053224; doi:10.3389/fnut.2026.1774451)
Supplement: Supplementary file 3 [file Supplementary_File_3.pdf]

Supplemental File 3: Evidence Table of Higher Education Institutions and the Use of MMCA Strategies to Encourage Customers to Select Plant-Rich Menu Options Categorized by Place, Profile, Portion, Pricing, Promotion, Picks, Priming/Prompting, and Proximity

***Supplemental Tables 1-8.** Higher Education Institutions and their Use of MMCA Strategies to Encourage Customers to Select Plant-Rich Menu Options Categorized by Place, Profile, Portion, Pricing, Promotion, Picks, Priming/Prompting, and Proximity

*Plant-rich is used interchangeably with plant-based or plant-forward throughout Supplemental Tables 1-8 to align with the extracted data from the included evidence sources

Supplemental Table 1. Place Strategies Used by Higher Education Institutions to Encourage Customers to Select Plant-Rich Menu Options

**Place Strategies Used by Higher Education Institutions to Encourage Customers to Select Plant-Rich Menu Options (*n*=2)**		
**Name of Higher Education Institution**	**Summary of MMCA Strategy** (Name of Program, Commitment, or Policy bolded if applicable)	**Evidence Source(s)**
Stanford University	- Encouragement for customers to select plant-rich menu options (i.e., vegetables) through the use of rotisseries, which are traditionally used for meat, to roast large quantities of whole and cut-up vegetable with the goal of stimulating students' senses (i.e., sight and smell) to select the fresh vegetables<br>- Reinforcement of healthy dietary messages by the inclusion of greenhouses and gardens at the entrance of the dining hall	"5 take aways from menu directions" (2016) (1); Tanyeri (2017) (2)
University of California, Irvine	- Encouragement for students to be "planteaters" through the placement of local garden on the patio and encased by a plexiglass door at food retailer on campus called The Anteatery	Crain (2019) (3)

Supplemental Table 2. Profile Strategies Used by Higher Education Institutions to Encourage Customers to Select Plant-Rich Menu Options

**Profile Strategies Used by Higher Education Institutions to Encourage Customers to Select Plant-Rich Menu Options (*n*=161)**		
**Name of Higher Education Institution**	**Summary of MMCA Strategy** (Name of Program, Commitment, or Policy bolded if applicable)	**Evidence Source(s)**
Alma College	- **Forward Food Pledge:** Commitment to transition at least 33% of meat-based entrees to plant-based entrees annually through the end of 2027	ForwardFood (2020) (4)
American University	- **Meatless Monday:** Participation in the "Meatless Monday" program by increasing plant-based entrees on Mondays and replacing meat items with vegetables or vegetables entrees on Monday	Middleton and Littler (2019) (5)
Antioch College	- **Forward Food Pledge:** Commitment to transition at least 33% of meat-based entrees to plant-based entrees annually through the end of 2027	ForwardFood (2020) (4)
Appalachian State University	- Expansion of healthy and sustainable plant-based menu options, such as vegan baked goods and whole food plant-based menu options, and new dining station with a plant-based concept called **Terra Verde**	AASHE (n.d.) (6); AppState (n.d.) (7); Cavanaugh (2011) (8); Hempen (2019) (9)
Arizona State University	- Expansion of plant-based menu options through the opening of plant-based food retailer, **Daily Root**, in 2015	AASHE (n.d.) (10); Middleton and Littler (2019) (5)
Bastyr University	- Implementation of dining program that serves predominantly vegan or vegetarian menu options with one meat option	US News and World Report (2011) (11)
Belmont University	- **Forward Food Pledge:** Commitment to transition at least 33% of meat-based entrees to plant-based entrees annually through the end of 2027	ForwardFood (2020) (4)
Benedict College	- **Forward Food Pledge:** Commitment to a 5% annual increase in plant-based menu options through 2024 and to transition at least 33% of meat-based entrees to plant-based entrees annually through the end of 2027<br>- **Meatless Monday:** Participation in the "Meatless Monday" program	ForwardFood (2020) (4); Johns Hopkins Center for a Livable Future

		and Meatless Monday (n.d.) (12); "Benedict College, Let's Meat Less, and Forward Food Join Forces to Expand Plant-Based Menu Offerings on the Midlands Campus" (2021) (13); "Benedict College and the Forward Food Collaborative join forces to expand plant-based menu offerings throughout campus" (2021) (14)
Bennington College	- Expansion of the ratio of meatless options (i.e., 66 meatless entrees on cafeteria menu)	"Vt.'s Bennington College said vegan friendly, VT" (2010) (15)
Bentley University	- **Forward Food Pledge:** Commitment to transition at least 33% of meat-based entrees to plant-based entrees annually through the end of 2027 by increasing the plant-based menu options at the 921 Dining Hall<br>- Commitment that 28% of entrees will be plant-based by December 31, 2024 with the plan to meet this goal through an increment of 5% annually	AASHE (n.d.) (16); Bentley University (n.d.) (17); ForwardFood (2020) (4); Walsh and Gowett (2023) (18)

Berry College	- Expansion of vegan menu options (i.e., vegan desserts, entrees, soups, grain bowls, and breakfast options)	"Berry College Dining Services provides food to students with all dietary restrictions including vegan, vegetarian and gluten-free restrictions. In the past six months, Dining Services has intentionally made changes in the food options to accommodate more" (2023) (19)
Binghamton University	- **Meatless Monday:** Participation in the "Meatless Monday" program through the expansion of two additional vegetarian options available for dinner and lunch<br>- Expansion of vegan options through a vegan station called **Gifts from the Garden**	AASHE (n.d.) (20); Bernstein (2013) (21); Van Caeseele, (2021) (22)
Boston College	- Commitment to make 50% of meals plant-based by 2027<br>- Introduction of new menu options and technologies to increase plant-rich menu options (i.e., seaweed-ish menu options) at **Tully Café**	Block (2024) (23); Buzalka (2023) (24)
Boston University	- **Coolfood Meals:** Launch of the Coolfood Meals program in partnership with the World Resources Institute in Spring 2024 and the introduction of 271 Coolfood Meals recipes<br>- Expansion of a kosher dining room including a completely vegan component and plant-based menu<br>- Procurement of over 20 different vegan and vegetarian options throughout the dining room each meal	AASHE (n.d.) (25); Boston University Dining Services (n.d.) (26); Boston University Dining Services (2023-2024) (27);

		Buzalka (2018) (28)
Bowdoin College	- Expansion of a variety of plant-rich menu options (i.e., vegetarian entrees, salads, and six varieties of veggie burgers) through local and organic fruits, vegetables, grains, and proteins	AASHE (n.d.) (29); Bowdoin (n.d.) (30); Yale Kamila (2015) (31)
Bowling Green State University	- Incorporation of seaweed products to increase plant-based menu options and shellfish alternatives	AASHE (n.d.) (32); Buzalka (2023) (33)
Brandeis University	- **Coolfood Pledge:** Commitment to a target of reducing food related greenhouse gas emissions by 25% by 2030 relative to a 2015 baseline	Coolfood (n.d.) (34); Brandeis Hospitality (n.d.) (35)
Brigham Young University	- Introduction of new plant-based recipes or products<br>- Expansion of plant-forward, low-impact meals through food retail concept called **Choices** established in 2019	AASHE (n.d.) (36); Humane World for Animals (2025) (37)
Bryn Mawr College	- **Meatless Monday:** Participation in the “Meatless Monday” program through the procurement of plant-based menu options	AASHE (n.d.) (38); “Local colleges are well-schooled in vegan dining options" (2016) (39)
Bucknell University	- Description of menu options that align with a plant-rich Mediterranean dietary pattern	AASHE (n.d.) (40); Kiernan (2010) (41)
California Polytechnic State University (Cal Poly)	- Expansion of plant-rich menu options through the introduction of meat substitution (vegan fried chicken), vegan and vegetarian food retailer (**Health Shack**), and food truck (**Plant Ivy**)	AASHE (n.d.) (42); Nazar (2022) (43)

California State University, Chico (Chico State)	- Expansion of vegetarian menu options	Mitchell (2012) (44)
Canisius University (formerly Canisius College)	- Expansion of plant-based menus through the "On the Go" concept and 2016 opening of plant-based food retail station, **Pitchforks**<br>- Collaboration with Hungry Planet to provide more meat substitutes through plant-based food retail station, **Pitchforks**	Middleton and Littler (2019) (5)
Carnegie Mellon University	- Expansion of vegan and vegetarian menu options by expanding the hours of plant-rich food retailer, **Evgefstos**<br>- Expansion of vegetarian and vegan options across other on campus dining locations including a 100% plant-based concept, **Garden Bistro**, launched in the Fall of 2017	AASHE (n.d.) (45); Galvin (2016) (46); Middleton and Littler (2019) (5)
Central Washington University	- Introduction of a variety of plant-based menu options through **Fresh Bar**, a healthy dining and wellness focused concept	AASHE (n.d.) (47); Buzalka (2019) (48)
Clark University	- Introduction of a variety of plant-rich desserts (i.e., vegan ice cream and cookies)	Chisholm (2019) (49)
Colby College	- Allocation of 70% of **Foss Dining Hall's** menu to vegetarian options compared to 20% at other dining halls<br>- **Meatless Monday:** Participation in the "Meatless Monday" program through expansion of vegan and vegetarian options	US News and World Report (2011) (11); Yale Kamila (2015) (31)
Colgate University	- **100% Plant-Forward Dining Initiative (through partnership with Chartwells Higher Education):** Introduction of a variety of plant-based menu options through temporary pop-up events to full dedicated plant-rich dining halls<br>- Increase of plant-based food options in the dining halls to decrease the climate impact of meals and a commitment to unique menus that emphasize less beef and more produce and whole grains	Buzalka (2021) (50); "Chartwells Higher Education Launches 100% Plant-Based Dining Hall Option in Colleges Nationwide - First-ever meatless residential dining hall puts

		sustainable, plant-based options at the center of the plate" (2021) (51); Colgate University (n.d.) (52); Colgate University Dining Services (n.d.) (53)
College of Charleston	- Introduction of a kosher and vegetarian dining hall<br>- **Meatless Monday:** Participation in the “Meatless Monday” program at campus dining halls	AASHE (n.d.) (54); Gruber (2015) (55)
College of the Atlantic	- Allocation of 1/3 of the menu to vegetarian options	Yale Kamila (2015) (31)
College of the Holy Cross	- Expansion of vegan options through a vegan station called **Lean and Green**	Chisholm (2019) (49)
Colorado Mountain College	- **Forward Food Pledge:** Commitment to transition at least 33% of meat-based entrees to plant-based entrees annually through the end of 2027<br>- Allocation of 45-50% of menu items as vegan menu options	AASHE (n.d.) (56); ForwardFood (2020) (4)
Colorado State University	- Introduction of new plant-based recipes or products	AASHE (n.d.) (57); Humane World for Animals (2025) (37)
Columbia University in the City of New York	- **Meatless Monday:** Participation in the “Meatless Monday” program and encouragement for students to reduce their consumption of animal products at least one day per week<br>- **Plant-Powered Carbon Challenge:** Commitment to New York City Mayor’s Office of Food Policy Challenge to make a 25% reduction in food-related carbon emissions by 2030 through the procurement of plant-powered food a target for 50% of items in every meal to be plant-based, and making plant-based menu items a regular feature across campus	"Langara College Becomes First Campus in Western Canada to Join Global Meatless Monday Initiative" (2015) (58); Columbia University in the City of New York (2023) (59);

		Columbia University in the City of New York (n.d.) (60); Schwartz (2019) (61)
Cornell University	- **The Purpose-Driven Plant-Based Incubator:** Participation in the Purpose-Driven Plant-Based Incubator program that offers college and university foodservices support to expand plant-forward menu options<br>- **Forward Food Pledge:** Commitment to transition at least 33% of meat-based entrees to plant-based entrees annually through the end of 2027 and 2021 commitment to increase plant-based menu options by 5-10% per year<br>- Expansion of a plant-based dining program through the reduction of meat purchases by 5% and increase of produce purchases by 9% in 2017<br>- Commitment to 45% plant-based menu options by the end of 2024 and 50% by the end of 2027	AASHE (n.d.) (62); Copman (2024) (63); Cornell Dining (n.d.) (64); Cornell University College of Human Ecology and Cornell University The Hotel School Cornell SC Johnson College of Business (2018) (65); ForwardFood (2020) (4); Wholesome Crave (2023) (66)
Davidson College	- **Forward Food Pledge:** Commitment to transition at least 33% of meat-based entrees to plant-based entrees annually through the end of 2027	ForwardFood (2020) (4)
DePaul University	- Expansion of vegan and vegetarian options through a new station	McBride (2019) (67)
Drexel University	- Introduction of vegan and vegetarian menu options through the meat-free food retailer, **Vegetate**, a new plant-forward food retail concept called **U.C. Veg**<br>- Collaboration with Aramark to develop additional plant-forward menu options	AASHE (n.d.) (68); Drexel University (n.d.) (69); Tanyeri (2014) (70); Taylor (2019) (71)

Duke University	- **Meatless Monday:** Participation in the "Meatless Monday" program through the increased availability of vegetarian and vegan menu options<br>- Expansion of plant-rich menu options through chefs across campus developing and serving a veggie burger through campus wide competition	Palmisano (2015) (72); The Menus of Change University Research Collaborative (n.d.) (73)
Endicott College	- **Forward Food Pledge:** Commitment to transition at least 33% of meat-based entrees to plant-based entrees annually through the end of 2027	AASHE (n.d.) (74); ForwardFood (2020) (4)
Fairfield University	- **Forward Food Pledge:** Commitment to transition at least 33% of meat-based entrees to plant-based entrees annually through the end of 2027	ForwardFood (2020) (4)
Florida Agricultural and Mechanical University	- **Partnership for a Healthier America's (PHA) Healthier Campus Initiative:** Commitment to offer a minimum of five fruits, five vegetables, and two 100% whole grain products during lunch and dinner	"Florida agricultural and mechanical university announces commitment to promote healthier options on campus, puts university at forefront of wellness cultural shift" (2018) (75)
Florida Institute of Technology (Florida Tech)	- Expansion of vegan menu options at the on-campus food retailer called the **Rathskeller**<br>- **Meatless Monday:** Participation in the "Meatless Monday" program through the provision of multiple vegan menu options in the main dining hall on campus	AASHE (n.d.) (76); "New Vegan Food Menus Arise on Campus" (2017) (77)
Florida State University	- **Plant Forward** program: Development of an eco-conscious program through changes such as vegan stations on campus	Florida State University (n.d.) (78)

Framingham State University	- **Forward Food Pledge:** Commitment to transition at least 33% of meat-based entrees to plant-based entrees annually through the end of 2027	ForwardFood (2020) (4)
Franklin & Marshall College	- **Forward Food Pledge:** Commitment to transition at least 33% of meat-based entrees to plant-based entrees annually through the end of 2027<br>- Expansion of plant-based menu option to align with Sodexo's sustainability initiative to be 50% plant-based by 2025	Franklin & Marshall College (n.d.) (79); ForwardFood (2020) (4)
Georgetown College	- **Forward Food Pledge:** Commitment to transition at least 33% of meat-based entrees to plant-based entrees annually through the end of 2027	ForwardFood (2020) (4)
Georgia Institute of Technology (Georgia Tech)	- Introduction of a vegan retail concept called **Slutty Vegan**<br>- Expansion of vegan and vegetarian menu option across campus with plant-based offerings and ingredients comprising over 30% of total purchases and offering plant-forward meals ~30% of the time	AASHE (n.d.) (80); Georgia Tech (n.d.) (81); Zusel (2023) (82)
Georgia Southern University	- **Forward Food Pledge:** Commitment to transition at least 33% of meat-based entrees to plant-based entrees annually through the end of 2027	ForwardFood (2020) (4)
Georgia State University	- Introduction of new plant-based recipes or products	Humane World for Animals (2025) (37)
Hamilton College	- **Meatfree Monday/Meatless Monday:** Participation in the "Meatfree Monday"/"Meatless Monday" program at McEwen Dining Hall	Kopp and Meade (2019) (83); "Hamilton College: JHS Program Provides Access and Opportunity" (2022) (84)
Harvard University	- **Coolfood Pledge:** Commitment to a target of reducing food related greenhouse gas emissions by 25% by 2030 relative to a 2015 baseline<br>- Introduction of a variety of plant-rich menu options through a "plant positive" focus and climate friendly and seasonal menu<br>- Allocation of 33% of menu options as vegan or vegetarian<br>- Expansion of plant-rich menu options through plant-forward **Bistro Bowl** concept on Thursday nights	Buzalka (2023) (85); Coolfood (n.d.) (34); Harvard Office for Sustainability (n.d.) (86); Harvard

		University (n.d.) (87); The Menus of Change University Research Collaborative (n.d.) (88)
Indiana University Bloomington	- Introduction of new plant-based recipes or products	Humane World for Animals (2025) (37)
Ithaca College	- **Forward Food Pledge:** Commitment to transition at least 33% of meat-based entrees to plant-based entrees annually through the end of 2027<br>- Expansion of vegan and vegetarian menu options and commitment to increasing plant-based menu consumption by 10% and decreasing animal protein consumption by 10%	AASHE (n.d.) (89); ForwardFood (2020) (4); "Ithaca College Dining Services Recognized for Outstanding Plant-based Menu Options" (2018) (90)
John Jay College of Criminal Justice	- Expansion of meat-free offerings with the help of MBJ Cafeteria Corp. in collaboration with the Humane World for Animals (formerly Humane Society of the United States)	Johns Hopkins Center for a Livable Future (n.d.) (91)
Johns Hopkins University	- **Meatless Monday:** Participation in the "Meatless Monday" program by offering vegan and/or vegetarian menu options instead of meat on Mondays during the academic year and encouragement to students to reduce their consumption of animal products at least one day per week	"Langara College Becomes First Campus in Western Canada to Join Global Meatless Monday Initiative" (2015) (58); Johns Hopkins

		University (n.d.) (92); Schwartz (2019) (61)
Johnson & Wales University (North Miami campus)	- Expansion of vegan and vegetarian options through dining services implementation of vegan day on Wednesdays	Sentenac (2015) (93)
Kent State University	- **Forward Food Pledge:** Commitment to serve 30% plant-based meals by 2026 and commitment to transition at least 33% of meat-based entrees to plant-based entrees annually through the end of 2027<br>- **The Purpose-Driven Plant-Based Incubator:** Participation in the Purpose-Driven Plant-Based Incubator program that offers college and university foodservices recipes, workshops, and collaboration to expand plant-forward menu options<br>- Partnership with the Humane World for Animals (formerly the Humane Society) to increase plant-based menu options over the next three years	AASHE (n.d.) (94); Block (2024) (23); Kent State University (n.d.) (95); ForwardFood (2020) (4); "Humane Society: Kent State University Doubles Its Plant-Based Pledge Ahead of National Nutrition Month" (2024) (96); "Nestlé Professional, Wholesome Crave and UMass Dining Launch Purpose-Driven Plant-Based Incubator™ to Accelerate Shift in College and University Menus" (2023) (97); Wholesome Crave (2023) (66)

Lee University	- **Forward Food Pledge:** Commitment to transition at least 33% of meat-based entrees to plant-based entrees annually through the end of 2027	ForwardFood (2020) (4)
Lehigh University	- **Forward Food Pledge:** Commitment to transition at least 33% of meat-based entrees to plant-based entrees annually through the end of 2027<br>- **Meatless Monday:** Participation in the "Meatless Monday" program at all student restaurants<br>- Commitment to increasing the percentage of plant-based foods through the **Sustainable and Healthful Food Purchasing Policy**<br>- Expansion of vegan and vegetarian menu options resulting in 62% of menu options being meat-free	AASHE (n.d.) (98); ForwardFood (2020) (4); Lehigh University (2021) (99); Lehigh University Dining (2024) (100)
Liberty University	- Introduction of a plant-forward retail concept called **The Hungry Herbivore**	Buzalka (2023) (101); Estrada (2022) (102)
Loyola Marymount University	- Procurement of vegan menu options and focus on tasty and nutritious options (i.e., vegan enchiladas)	Gingerella (2019) (103)
Madonna University	- **Forward Food Pledge:** Commitment to transition at least 33% of meat-based entrees to plant-based entrees annually through the end of 2027	ForwardFood (2020) (4)
Maharishi International University (formerly Maharishi University of Management)	- Description of dining program that exclusively serves vegan or vegetarian menu options	US News and World Report (2011) (11)
Marist University (formerly Marist College)	- **Forward Food Pledge:** Commitment to transition at least 33% of meat-based entrees to plant-based entrees annually through the end of 2027	ForwardFood (2020) (4)
Michigan State University	- Introduction of plant-based protein recipes through recipe development that utilizes regional ingredients (i.e., lentils, beans, soy or grains) and reduces animal proteins	Michigan State University Division of Residential and Hospitality Services (2021) (104)
Minnesota State University, Mankato	- Description of a variety of plant-rich menu options	Gingerella (2019) (103)

Montclair State University	- Procurement of black bean and tofu "crab" cake menu options<br>- Expansion of plant-forward menu options	Cobe (2019) (105); Montclair State University Dining Services (n.d.) (106)
Moravian University	- **Forward Food Pledge:** Commitment to transition at least 33% of meat-based entrees to plant-based entrees annually through the end of 2027	ForwardFood (2020) (4)
Nazareth University (formerly Nazareth College)	- **Forward Food Pledge:** Commitment to transition at least 33% of meat-based entrees to plant-based entrees annually through the end of 2027	ForwardFood (2020) (4)
New York University	- **Coolfood Pledge:** Commitment to a target of reducing food related greenhouse gas emissions by 25% by 2030 relative to a 2015 baseline through an expansion of plant-based menu options<br>- **Plant-Powered Carbon Challenge:** Commitment to New York City Mayor's Office of Food Policy Challenge to make a 25% reduction in food-related carbon emissions by 2030 through the procurement of plant-powered food<br>- A variety of food retail concepts/stations with a focus on plant-based menu items	AASHE (n.d.) (107); Coolfood (n.d.) (34); New York University (n.d.) (108); New York University (2020) (109)
North Carolina State University	- **Meatless Monday:** Participation in the "Meatless Monday" program by offering primarily plant-forward menu options at Fountain Dining Hall on Mondays<br>- Description of menu option that aligns with a plant-forward dietary pattern, efforts to expand plant-forward menu options, and introduction of new plant-based recipes or products	AASHE (n.d.) (110); Cobe (2017) (111); Humane World for Animals (2025) (37)
Northeastern University	- **100% Plant-Forward Dining Initiative (through partnership with Chartwells Higher Education):** Introduction of a variety of plant-rich menu options through temporary pop-up events to full dedicated plant-rich dining halls<br>- Description of cooking techniques for appealing vegetarian menu options that align with a plant-rich dietary pattern<br>- Introduction of a planetary plate in some dining halls consisting of half vegetables and meat as a garnish	AASHE (n.d.) (112); Buzalka (2021) (50); Fitzpatrick (2013) (113); McBride (2019) (67); "Chartwells Higher Education Launches 100% Plant-Based Dining Hall

		Option in Colleges Nationwide - First-ever meatless residential dining hall puts sustainable, plant-based options at the center of the plate" (2021) (51)
Northern Arizona University	- **Meatless Monday:** Participation in the "Meatless Monday" program<br>- Expanding plant-rich menu options by embracing a plant-forward strategy in crafting menus for resident dining halls and prioritizing plant-based offerings	AASHE (n.d.) (114)
Northern Michigan University	- **Forward Food Pledge:** Commitment to transition at least 33% of meat-based entrees to plant-based entrees annually through the end of 2027	AASHE (n.d.) (115); ForwardFood (2020) (4)
Northwestern University	- **Meatless Monday:** Participation in the "Meatless Monday" program by increasing the amount of vegetarian and vegan menu options in dining halls across campus on Mondays<br>- Description of a variety and range of menu options that align with a plant-rich dietary pattern and description of substituting plant-rich ingredients for meat, dairy, and egg products	Cobe (2017) (111); Fitzpatrick (2012) (116); Northwestern Dining (2013) (117); "Sodexo Earns 'Most Vegan-Friendly College in America' at Northwestern University - Northwestern University is named top pick by PETA2 for the second time in the

		category of small colleges" (2010) (118); Northwestern tops list of most vegan-friendly colleges (2011) (119); Warmouth (2014) (120)
Ohio University	- Introduction of more plant-forward menu items and transforming familiar dishes to be plant-forward	The Menus of Change University Research Collaborative and Stanford Food Institute Residential & Dining Enterprises (2024) (121)
Oklahoma City University	- Introduction of a variety of plant-rich menu options through new vegan raw bar in 2010	Allen (2013) (122)
Oregon State University	- **Forward Food Pledge:** Commitment to transition at least 33% of meat-based entrees to plant-based entrees annually through the end of 2027<br>- Commitment to have 50% of menu options be plant-based by 2027<br>- Introduction of new plant-based recipes or products<br>- Development of plant-based dishes that are craveable, innovative, and familiar	ForwardFood (2020) (4); Humane World for Animals (2025) (37); Oregon State University (n.d.) (123); The Menus of Change University Research Collaborative (n.d.) (73)

Providence College	- Expansion of plant-rich menu options through partnership with Sodexo **Rustic Roots** plant-based concept	Buzalka (2021) (124); Providence College (2021) (125)
Quinnipiac University	- **Meatless Monday:** Introduction of the “Meatless Monday” program at a food station to reduce meat options on campus once a week	Buzalka (2019) (126)
Rice University	- Procurement of a variety of plant-based meat alternatives (i.e., plant-based brisket)	Vaughn (2018) (127)
Rider University	- **Meatless Monday:** Participation in the “Meatless Monday” program through the provision of new vegetarian and vegan menu options<br>- **Wellness Wednesday:** Participation in the “Wellness Wednesday” through the provision of new vegetarian and vegan menu options<br>- Expansion of vegan and vegetarian options	Abdur-Rahman (2018) (128); Rider University Dining Services (n.d.) (129)
Roanoke College	- **Forward Food Pledge:** Commitment to transition at least 33% of meat-based entrees to plant-based entrees annually through the end of 2027	ForwardFood (2020) (4)
Rochester Institute of Technology	- Commitment to having 50% plant-based offerings by 2025 in collaborations with the Humane World for Animals (formerly the Humane Society of the United States)	Humane World for Animals (2023) (130)
Rollins College	- **Forward Food Pledge:** Commitment to transition at least 33% of meat-based entrees to plant-based entrees annually through the end of 2027	ForwardFood (2020) (4)
Rutgers, The State University of New Jersey (Rutgers University)	- Introduction of plant-rich menu options that incorporate vegetables, whole grains, nuts, and beans as the center of the plate while serving red meat less<br>- Commitment to have 50% of menu options be plant-based or low-carbon by 2024 and commitment to offer sustainable menus with at least 50% plant-based or low carbon footprint entrees for all meal periods by 2026<br>- Introduction of new plant-based recipes or products<br>- Expansion of plant-based menu options by utilizing the 1:1 concept and offering one plant-based menu options for every animal-based menu option	AASHE (n.d.) (131); Dwyer (2020) (132); Humane World for Animals (2025) (37); Rutgers Dining Services (n.d.) (133); Taylor (2017) (134)
Salisbury University	- **Meatless Monday:** Participation in the “Meatless Monday” program and expansion of plant-rich menu options	"Salisbury university dining services earns a on

		peta2 vegan report card" (2016) (135); "Salisbury university dining services earns a on peta2 vegan report card" (2017) (136)
San Diego State University	- **Meatless Monday:** Recipe development of a salsa to top "Meatless Monday" offerings and description of weekly Meatless Monday program for the Faculty Staff Club<br>- Description of menu options that align with a plant-rich Mediterranean dietary pattern	AASHE (n.d.) (137); Kiernan (2010) (41); Fitzpatrick (2012) (138); Parseghian, (2012) (139); San Diego Union-Tribune (2016) (140)
Seattle Pacific University	- Expansion of plant-based menu options and description of one plant-rich breakfast option	Cobe (2020) (141)
Seattle University	- Introduction of a 100% plant-based concepts, such as the new **Convergence Zone**	AASHE (n.d.) (142); Buzalka (2023) (33), Buzalka (2023) (101), Redhawk Dining (n.d.) (143)
Skidmore College	- Expansion of plant-rich options through a new vegetarian and plant-forward station called **Emily's Garden**<br>- Expansion of plant-rich options through all-vegan bar with rotating menu and purchase of fresh vegetables from the Skidmore Community Garden<br>- **Low Impact Wednesday:** Participation in Low Impact Wednesday through the provision of a plant forward menu for breakfast, lunch, and dinner and reduction of meat proteins on the menu by 60%	Cobe (2017) (111); Cobe (2018) (144); Johns Hopkins Center for A Livable Future and Meatless Monday (n.d.) (145); Skidmore College (n.d.) (146);

		Skidmore College (n.d.) (147)
Smith College	- **Forward Food Pledge:** Commitment to transition at least 33% of meat-based entrees to plant-based entrees annually through the end of 2027<br>- Allocation of 47% of food purchases to plant-based purchases<br>- Commitment to make 55% of meals plant-based by 2025	Block (2024) (23); ForwardFood (2020) (4); Smith College (n.d.) (148)
Southern University and Agricultural & Mechanical College	- Introduction of new plant-based recipes and menu options through a plant forward initiative	"Campus Dining: Experience or New Dining Food Options" (2021) (149)
St. John Fisher University (formerly St. John Fisher College)	- **100% Plant-Forward Dining Initiative (through partnership with Chartwells Higher Education):** Introduction of a variety of plant-rich menu options through temporary pop-up events to full dedicated plant-rich dining halls<br>- Procurement of a variety of meatless and vegan menu options through partnership with **Chartwells Higher Education Root for You** initiative	Buzalka (2021) (50); Buzalka (2021) (150); "Chartwells Higher Education Launches 100% Plant-Based Dining Hall Option in Colleges Nationwide - First-ever meatless residential dining hall puts sustainable, plant-based options at the center of the plate" (2021) (51)
Stanford University	- **Eat Well @ Stanford:** Allocation of plant-rich menu options across menus representing 50% vegan and 80% vegetarian menu options and support of vegan and vegetarian dietary patterns	"Viewpoint: How Campus Dining Supports Stanford's New

	- **One Plate, One Planet:** Sustainable food program with six pillars: 1) Climate-smart dining, especially food waste reduction and advancing plant-forward diets; 2) racial equity and supporting Black businesses; 3) Curbing deforestation through supply chain pressures; 4) Thriving oceans; 5) Catalyzing a circular economy of food; and 6) embracing systems thinking<br>- Introduction of roasted vegetables in dining rotisseries in which are traditionally used for meats<br>- Commitment to reduce food-related greenhouse gas emission by 25% by 2030 through plant-forward shifts across dining programs	Neighborhood Model" (2021) (151); "5 take aways from menu directions" (2016) (1); Residential & Dining Enterprises Stanford Dining, Hospitality & Auxiliaries (n.d.) (152); Residential & Dining Enterprises Stanford Dining's Sustainable Food Program (n.d.) (153); Stanford Dining, Hospitality & Auxiliaries Residential & Dining Enterprises (n.d.) (154); Stanford Dining, Hospitality & Auxiliaries Residential & Dining Enterprises (n.d.) (155); Tanyeri (2017) (2)
Stony Brook University	- **Forward Food Pledge:** Commitment to transition at least 33% of meat-based entrees to plant-based entrees annually through the end of 2027	ForwardFood (2020) (4)
Syracuse University	- **Meatless Monday:** Participation in the "Meatless Monday" program through expansion and variety of vegan and vegetarian options	AASHE (n.d.) (156); David

	- Increase of plant-based proteins and menu options	George (2010) (157); King (2013) (158); Syracuse University (n.d.) (159); US News and World Report (2011) (11)
The Ohio State University	- Introduction of a variety of plant-rich menu options through new on campus food truck called **Thyme & Change 2.0** with a vegan menu that features plant-rich counterparts to animal-sourced options<br>- Expansion of vegan and vegetarian options and meat substitutions and introduction of new plant-based recipes or products<br>- Allocation to 30% of the university's food offerings as vegan menu options	AASHE (n.d.) (160); Buzalka (2019) (161); Cagle (2019) (162); Humane World for Animals (2025) (37); Lerner (2018) (163); Parismcgee (2017) (164); Schwartz (2019) (61); "Dinner is different: The newest in campus dining" (2019) (165)
The Pennsylvania State University (Penn State)	- Commitment to increase plant-based options to 35% of entrees by 2025 that was rescinded soon after	AASHE (n.d.) (166); Buzalka (2023) (101); Healey (2023) (167); Humane World for Animals (2025) (37)
The University of Arizona	- Commitment to have 40% of menu options be plant-based by 2025<br>- Introduction of new plant-based recipes or products with an emphasis on plant-centered plates using whole foods that are minimally processed	AASHE (n.d.) (168); Humane World for Animals

	- Collaboration with Partnership for a Healthier America's Healthier Campus Initiative to prioritize plant-based options<br>- Expansion of plant-rich menu options through recently opened plant-based food retail concept called **Radiccio**	(2025) (37); The University of Arizona (n.d.) (169)
The University of Iowa	- **Meatless Monday:** Participation in the "Meatless Monday" program through the provision of meat-free menu options for students every first Monday of the month	Meatless Monday (n.d.) (170); Miller (2018) (171); "University of Iowa holds first Meatless Monday at dining halls" (2018) (172)
The University of Oklahoma	- Introduction of new plant-based recipes or products	AASHE (n.d.) (173); Humane World for Animals (2025) (37)
The University of Texas at Austin	- **Coolfood Pledge:** Participation in the Coolfood Pledge to decrease food-related greenhouse gas emissions by 25% by 2030<br>- **Forward Food Pledge:** Commitment to transition at least 33% of meat-based entrees to plant-based entrees annually through the end of 2027<br>- Commitment to have 50% of menu options be plant-based by 2024<br>- Introduction of new plant-based recipes or products (i.e., plant-based desserts and soups)	AASHE (n.d.) (174); Block (2024) (23); Coolfood (n.d.) (34); ForwardFood (2020) (4); Humane World for Animals (2024) (175); Humane World for Animals (2025) (37); "World Resources Institute: PepsiCo, University of Texas at Austin, ISS, Healthcare Systems Pledge to

		Serve Planet-Saving 'Cool Food'" (2022) (176)
The University of Utah	- Expansion of plant-rich menu options through a new food retailer with a fully plant-based menu called **Rooted**	AASHE (n.d.) (177); "Rooted Offers New Plant-Based Dining Options on Campus" (2018) (178)
Towson University	- Provision of soy-based beef and chicken alternative menu options through the **T-Veggie** vegetarian program since 2009<br>- **T-Vegan:** Introduction of meat free and vegan menu options (i.e., coconut curry tofu and lentil sloppy joes) and plant-based meat alternative (i.e., vegan chicken cheese steaks)<br>- Commitment to reduce greenhouse gas emissions through increased plant-based options in dining halls and through catering	Obineme (2013) (179); "Towson University in the Running for Most Vegan-Friendly College in the U.S" (2012) (180); Townson University (2021) (181); Towson University (n.d.) (182)
Tulane University	- **Forward Food Pledge:** Commitment to transition at least 33% of meat-based entrees to plant-based entrees annually through the end of 2027<br>- **Meatless Monday:** Participation in the "Meatless Monday" program and encouragement to students to reduce their consumption of animal products at least one day per week	ForwardFood (2020) (4); Schwartz (2019) (61)
University at Albany	- Expansion of vegan menu items	AASHE (n.d.) (183); Buzalka (2018) (184)

University at Buffalo	- **Plant-Powered Mondays:** Efforts to ensure plant-forward menu options are available across campus<br>- **Strictly Vegetarian:** Efforts to ensure plant-forward menu options are available across campus	Humane World for Animals (2025) (37)
University of California, Berkeley	- **The Purpose-Driven Plant-Based Incubator:** Participation in the Purpose-Driven Plant-Based Incubator program that offers college and university foodservices support to expand plant-forward menu options<br>- **Meatless Monday:** Participation in the "Meatless Monday" program<br>- Commitment to transition 50% of menu options offered to be plant-based by 2027 through partnership with the Humane World for Animals (formerly the Humane Society of US)<br>- **Plant-Forward Recipe Challenge:** Bi-annual competition among campus chefs to create popular plant-based recipes with the winning menu option being permanently added to the menu<br>- **Forward Food Pledge:** Commitment to transition at least 33% of meat-based entrees to plant-based entrees annually through the end of 2027<br>- Introduction of new plant-based recipes or products	"Langara College Becomes First Campus in Western Canada to Join Global Meatless Monday Initiative" (2015) (58); ForwardFood (2020) (4); Humane World for Animals (2025) (37); "UC Berkeley and The Humane Society Collaborate to Make Campus Dining More Plant-Based" (2023) (185); Sanchez (2022) (186); The Menus of Change University Research Collaborative (n.d.) (88); The Menus of Change University Research

		Collaborative (n.d.) (73); Wholesome Crave (2023) (66)
University of California, Davis	- Commitment to procure 25% sustainable and plant-forward food and beverages purchased by 2030<br>- Introduction of new plant-based recipes or products<br>- Allocation of 33% of food and beverage purchases to plant-based products	AASHE (n.d.) (187); Humane World for Animals (2025) (37); UC Davis (2021) (188)
University of California, Irvine	- Implementation and consistent offerings of plant-forward menu options at on campus food retailers (i.e., **The Anteatery** and **Brandywine** dining hall)	AASHE (n.d.) (189)
University of California, Los Angeles	- Introduction of plant-based menu options through new food retailer on campus called **Veggie Grill**<br>- **Meatless Monday:** Participation in the "Meatless Monday" program through provision of a variety of vegetarian dishes expansion of non-meat options during lunch and dinner on Mondays to encourage sustainable eating<br>- **Green Mondays:** Participation in the "Green Monday" program in which one platform in every dining hall serves vegan menu options<br>- **Beefless Thursday:** Participation in the "Beefless Thursday" program through the removal of beef on Thursdays in the main dining halls<br>- Expansion of vegan and vegetarian menu options through food retailer on campus opened in 2013 called **Bruin Plate**<br>- **Forward Food Pledge:** Commitment to transition at least 33% of meat-based entrees to plant-based entrees annually through the end of 2027<br>- Commitment to have 50% of menu options be plant-based by 2027<br>- Introduction of new plant-based recipes or products<br>- Expansion of plant-rich menu options through innovation of menus to be more inclusive of vegan and vegetarian menu options	AASHE (n.d.) (190); Alamdari (2018) (191); UCLA Annual Foodservice Sustainability Policy Report (2018–2019) (192); Annual UCLA Foodservices Sustainability Report (2010-2011) (193); Block (2024) (23); Cleveland and Jay (2020) (194); "UCLA's New All-Healthy Dining Hall" (2014) (195); ForwardFood

		(2020) (4); Humane World for Animals (2025) (37); Smith (n.d.) (196); "UCLA, a soon to be vegan campus? (2012) (197); UCLA Dining Services (n.d.) (198); UCLA Sustainability (n.d.) (199)
University of California, Riverside	- **Beefless Fridays:** Participation in Beefless Fridays program by not featuring beef on the menu at the Residential Restaurant one day per week<br>- Reduction of emission through plant-based menu options through the allocation of 20% of the menu to vegan options and 30% of the menu to vegetarian options<br>- Allocation of approximately 27% of menu options at the Residential Restaurant as vegan or vegetarian menu options<br>- **Forward Food Pledge:** Commitment to transition at least 33% of meat-based entrees to plant-based entrees annually through the end of 2027	ForwardFood (2020) (4); Plascencia (2013) (200); UC Riverside Dining Services (n.d.) (201)
University of California, San Diego	- **Meatless/Beefless Monday:** Participation in a the "Meatless Monday" program/"Beefless Monday" program by removing beef from certain dining hall concepts<br>- **Forward Food Pledge:** Commitment to transition at least 33% of meat-based entrees to plant-based entrees annually through the end of 2027<br>- **Rooted in Flavor:** Challenge for chefs to create delicious, plant-forward menu options<br>- Introduction of a variety of plant-rich menu options through a vegan eatery and lounge and new plant-based recipes or products<br>- Expansion of plant-based menu options through new food retailer on campus with a focus on plant-based versions of classic menu options (i.e., burgers, chicken tenders, and milkshakes) called **Plant Power Fast Food**<br>- Goal to make 65% of dining purchases plant-based during 2023-2026	AASHE (n.d.) (202); Allen (2013) (122); ForwardFood (2020) (4); Humane World for Animals (2025) (37); Mitchell (2021) (203); McCord (2010) (204); "Vegan Fast-food Chain

		Opens Second College Campus Location - Plant Power Fast Food hits significant expansion milestone with the opening of its newest location on the campus of the University of California at San Diego" (2021) (205); San Diego Union-Tribune (2016) (140); Schwartz (2019) (61); The Menus of Change University Research Collaborative and Stanford Food Institute Residential & Dining Enterprises (2024) (121); UC San Diego (n.d.) (206)
University of California, Santa Barbara	- **The Purpose-Driven Plant-Based Incubator:** Participation in the Purpose-Driven Plant-Based Incubator program that offers college and university foodservices recipes, workshops, and collaboration to expand plant-forward menu options<br>- Introduction of plant-based menu options including vegan desserts	AASHE (n.d.) (207); "Nestlé Professional, Wholesome Crave

	- **Climate Friendly Monday:** Reducing meat and dairy menu options and increasing local, organic, seasonal, and whole food menu options on Mondays twice a quarter	and UMass Dining Launch Purpose-Driven Plant-Based Incubator™ to Accelerate Shift in College and University Menus," (2023) (97); The Menus of Change University Research Collaborative (n.d.) (73)
University of California, Santa Cruz	- Description of comfort foods menu options that are plant-based<br>- **Meat-Free Monday:** Participation in a "Meat-free Monday" program by making one of five dining halls on campus going completely meatless every Monday on a rotating basis	Cobe (2015) (208); McCord (2010) (204)
University of Central Florida	- **Meat-Free Monday:** Participation in a "Meat-Free Monday" program	McCord (2010) (204)
University of Colorado Boulder	- Commitment to have 75% of menu options be plant-based by 2025<br>- Introduction of new plant-based recipes, products, and menu items	Humane World for Animals (2025) (37); The Menus of Change University Research Collaborative (n.d.) (73)
University of Colorado Colorado Springs	- **Bring Food Forward:** Introduction of plant-based dishes as a results of training workshop among kitchen staff and student employees on how to make 40 different plant-based menu options in collaboration with the Humane World for Animals (formerly Human Society of the United States)	"UCCS Dining and Hospitality to add more plant-based dishes to campus eateries" (2017)

	- Expansion of plant-rich menu options through an established goal to allocation 70% of menu options as plant-based in 2025 and a “plant forward first” focus on plant-based items and fresh vegetables during food purchasing	(209); University of Colorado Colorado Springs (n.d.) (210)
University of Connecticut	- Introduction of a new plant-based café on campus<br>- Expansion of plant-rich menu options through introduction of meatless café, **CrossRoads**, and food truck, **Food for Thought**, with an emphasis on plant-based meals	AASHE (n.d.) (211); Buzalka (2021) (124); Dunne (2022) (212); Humane World for Animals (2025) (37)
University of Dayton	- **Forward Food Pledge:** Commitment to increase plant-based menu options to 30% by 2025 and commitment to transition at least 33% of meat-based entrees to plant-based entrees annually through the end of 2027<br>- Commitment to increase spending on plant-based foods by 45% within 5 years from a 2022 baseline<br>- Commitment to increase the number of plant-based menu options by 30% from 2022 to 2027	Estrada (2023) (213); ForwardFood (2020) (4); Healey (2023) (167); "Humane Society: University of Dayton and Forward Food Collaborative Announce Joint Effort to Increase Plant-Based Options" (2021) (214); "Humane Society: University of Dayton Leads the Way in Swift Plant-Based Menu Changes" (2023) (215); University

		of Dayton (n.d.) (216)
University of Florida	- Introduction of a variety of plant-rich menu options, snacks (i.e., meatless jerky, hot cereal cops, vegetable chips, and energy bars), and vegan comfort foods and menu options (i.e., vegan mac and cheese)	Gingerella (2019) (103); "Dozens of New Vegan Options Spring Up at University of Florida" (2014) (217)
University of Georgia	- Introduction of new plant-based recipes or products<br>- Commitment to transition 36-40% of menus to be plant-based	Humane World for Animals (2025) (37)
University of Hawaii at Mānoa	- **Meatless Monday:** Participation in the "Meatless Monday" program<br>- Description of offering over 120 vegan and vegetarian menu options as a commitment to better serve vegan, vegetarian, and flexitarian customers	"Peta2 Recognizes Sodexo at the University of Hawaii at Manoa for Vegan Cuisine" (2018) (218)
University of Illinois Urbana-Champaign	- Expansion of plant-rich menu options through new and inventive ways to change flavors and textures of plant-based proteins and multiple vegan entrees and sides at every meal	AASHE (n.d.) (219); Cobe (2016) (220); Humane World for Animals (2025) (37)
University of Maine at Presque Isle	- Introduction of more vegan and vegetarian entrees (i.e., vegan chicken fajitas)	Yale Kamila (2015) (31)
University of Maryland, College Park	- **Meat-Free Monday:** Participation in a meat-free Monday program<br>- **Coolfood Pledge:** Participation in the Coolfood Pledge to decrease food-related greenhouse gas emissions by 25% by 2030 through the development of creative ways to use less animal products and more plant-based foods<br>- Expansion of vegan and gluten-free baked goods and substitution of animal products (i.e., eggs) for plant-sourced ingredients (i.e., pear or apple) and introduction of new plant-based recipes or products	Cavanaugh (2011) (8); Humane World for Animals (2025) (37); Curtis (2019) (221); McCord (2010) (204); "World

		Resources Institute: Maryland Becomes the First 'Cool Food' University" (2019) (222); The Menus of Change University Research Collaborative (n.d.) (88)
University of Massachusetts Amherst	- **The Purpose-Driven Plant-Based Incubator (through partnership with Nestlé Professional and Wholesome Crave):** Introduction of a variety of plant-rich menu options through recipe development efforts<br>- **Making Local, Healthy, Sustainable Delicious: The How-To Guide for Foodservice Operators:** Development of dining menu program that emphasizes a reduction in sodium, increase in fruits and vegetables, portion control, whole grains, plant-based proteins, and sustainable seafood menu options<br>- **Sanitas Per Escam ("Health Through Food"):** Development of healthy and sustainable menu options that promote nutrient density and the consumption of fruits and vegetables, high quality fats, and the reduction of saturated fats<br>- **Hampshire Dining Commons:** Expansion of plant-based seasonal items through the opening of new dining hall<br>- Collaboration with Nasoya to implement new healthy and plant-forward menu options for students	Buzalka (2018) (223); Buzalka (2023) (224); Buzalka (2023) (101); “UMass Goes All In on Health with Dining Renovation” (2013) (225); "As Demand for Plant-Based Products Soars, Nasoya Expands to Food Service with Key University and Fast Casual Restaurant Accounts with Popular Plantspired™ Line - America's #1 Tofu Brand

		Anticipates Plant-Based Meal Solutions Line Sales to Double in 2022" (2021) (226); "Nestlé Professional, Wholesome Crave and UMass Dining Launch Purpose-Driven Plant-Based Incubator™ to Accelerate Shift in College and University Menus" (2023) (97); “Third-party Certification Improves Healthy Option Consumption at UMass” (2013) (227); Tierney (2024) (228); Wholesome Crave (2023) (66)
University of Miami	- Introduction of a variety of plant-rich meatless menu options (i.e., flexitarian and vegan)	Bolivar (2010) (229); "Vegan-friendly options increasing in campus dining halls" (2018) (230); University

		of Miami Dining (n.d.) (231)
University of Michigan-Ann Arbor	- **Forward Food Pledge:** Commitment to transition at least 33% of meat-based entrees to plant-based entrees annually through the end of 2027<br>- **Sustainable Mondays:** Reduction of the provision of red meat and processed red meat (i.e., beef, lamb, goat, pork, and pepperoni) once a week; initiated in 2017 and expanded to all dining halls in 2019 with the aim to offer a more sustainable menu that focuses on plant-based proteins, poultry, and fish while decreasing red meat options<br>- Commitment to increase plant-based options to 55% of entrees by 2025 in collaboration with the Humane World for Animals (formerly the Humane Society of the United States)<br>- Introduction of a variety of plant-rich menu options through Proteins Foundations recipes and introduction of new plant-based recipes or products<br>- Expansion of plant-rich menu options through partnership with Michigan Bean Commission<br>- Policy to provide plant-based options at each meal	Buzalka (2022) (232); Buzalka (2023) (101); ForwardFood (2020) (4); Healey (2023) (167); Humane World for Animals (2025) (37); Lambrecht et al. (2023) (233); McCarty (2021) (234); Michigan Dining (n.d.) (235); Tanyeri (2017) (2)
University of Minnesota Duluth	- **The Purpose-Driven Plant-Based Incubator:** Participation in the Purpose-Driven Plant-Based Incubator program that offers college and university foodservices recipes, workshops, and collaboration to expand plant-forward menu options	"Nestlé Professional, Wholesome Crave and UMass Dining Launch Purpose-Driven Plant-Based Incubator™ to Accelerate Shift in College and University Menus" (2023) (97); Wholesome Crave (2023) (66)
University of Nebraska-Lincoln	- Introduction of new plant-based recipes or products	AASHE (n.d.) (236); Humane

		World for Animals (2025) (37)
University of New Hampshire	- Expansion of plant-based proteins and reduction of the use of red meat by 20%	DeChellis (2017) (237)
University of North Carolina at Chapel Hill	- **Meatless Monday:** Participation in the "Meatless Monday" program	Meyer (2019) (238)
University of North Texas	- **Forward Food Pledge:** Commitment to transition at least 33% of meat-based entrees to plant-based entrees annually through the end of 2027<br>- Commitment to have 50% of menu options be plant-based by 2025<br>- Introduction of new plant-based recipes, products, and a variety of plant-rich menu options (i.e., plant-based burgers) through the first all-vegan cafeteria on a Higher Education Institutions campus, **Mean Greens**, (opened in 2011), and plant-forward concept (i.e., Leaf) to utilize plant-sourced ingredients and greens grown on campus	Allen (2013) (122); Buzalka (2021) (239); ForwardFood (2020) (4); Gingerella (2019) (103); Humane World for Animals (2025) (37); Schwartz (2019) (61); Middleton and Littler (2019) (5); The Menus of Change University Research Collaborative (n.d.) (88); The Menus of Change University Research Collaborative and Stanford Food Institute Residential & Dining Enterprises (2024) (121); University of

		North Texas Dining Services (n.d.) (240); "Local colleges win 'Most Vegan-Friendly' award" (2011) (241); White (2019) (242)
University of Notre Dame	- Allocation of 23% vegetarian entrees and 18% vegan entrees at two traditional dining halls	McBride (2019) (67)
University of Oregon	- Introduction of more vegan and vegetarian options due to the lower carbon footprint of plant-based foods compared to animal-sourced options	AASHE (n.d.) (243); Halnon (2024) (244)
University of Pennsylvania	- Commitment to provide diverse vegan and plant-based menu options on campus	AASHE (n.d.) (245)
University of Pittsburgh	- Expansion of plant-forward menu options<br>- **Coolfood Pledge:** Commitment to a target of reducing food related greenhouse gas emissions by 25% by 2030 relative to a 2015 baseline	AASHE (n.d.) (246); AASHE (n.d.) (247); Pitt Sustainability (n.d.) (248); Schackner (2021) (249)
University of Portland	- Expansion of plant-rich menu options through the development of new seasonal, vegetarian, and local menu options	Barlett (2011) (250)
University of Rochester	- **Meatless Monday:** Description of Meatless Monday as a campus event	Dowd (2015) (251)
University of San Diego	- **Meatless Monday:** Participation in the "Meatless Monday" program<br>- Expansion of plant-rich menu options through the incorporation of more plant-based ingredients into menus	AASHE (n.d.) (252); San Diego Union-Tribune (2016) (140); University of San Diego (n.d.) (253)

University of South Carolina	- **Meatless Monday:** Participation in the "Meatless Monday" program	Johns Hopkins Center for a Livable Future & Meatless Monday (n.d.) (12)
University of South Florida	- Expansion of vegan menu options (i.e., black bean burgers, tofu pho, etc.)	"University of South Florida in the Running for Most Vegan Friendly College in the U.S." (2012) (254)
University of Southern California	- **EcoMonday:** Participation in the "EcoMonday" across three main dining halls in which red meat is not served<br>- Introduction of new plant-based recipes or products	AASHE (n.d.) (255); Humane World for Animals (2025) (37)
University of Vermont	- Allocation of 78.8% of menu items to vegan/vegetarian<br>- Commitment for 50% of the entrees in the dining halls to be plant-based by 2025	Baxley (2021) (256); University of Vermont (n.d.) (257)
University of Virginia	- **Meatless Monday:** Participation in the "Meatless Monday" program through expansion of vegan and vegetarian options<br>- **Sustainable Food Action Plan:** Includes the commitment to increase plant-based menu options in the next 10 years<br>- **Veg Out:** Participation in "Veg Out" program at least once a semester in which one dining hall will feature and all-Vegan meal period	AASHE (n.d.) (258); US News and World Report (2011) (11); "LETTER: The University strives for more sustainable, plant-based dining options" (2020) (259); UVA Dine (n.d.) (260); UVA Sustainable Food

		Collaborative, A Task Force of the UVA Environmental Stewardship Subcommittee on Sustainability (2021-2030) (261)
University of Washington	- **Food Literacy Program:** Introduction of new recipes that align with a plant-rich dietary pattern<br>- Commitment to have 48% of menu options be plant-based by 2025<br>- Introduction of new plant-based recipes or products	Fitzpatrick (2018) (262); Humane World for Animals (2025) (37)
University of Wisconsin-Madison	- **The Purpose-Driven Plant-Based Incubator:** Participation in the Purpose-Driven Plant-Based Incubator program that offers college and university foodservices recipes, workshops, and collaboration to expand plant-forward menu options<br>- **Forward Food Pledge:** Commitment to transition at least 33% of meat-based entrees to plant-based entrees annually through the end of 2027<br>- Introduction of new plant-based recipes or products<br>- Commitment to increase plant-based options to 30% of entrees by 2025	Buzalka (2023) (101); ForwardFood (2020) (4); Healey (2023) (167); Humane World for Animals (2025) (37); "Nestlé Professional, Wholesome Crave and UMass Dining Launch Purpose-Driven Plant-Based Incubator™ to Accelerate Shift in College and University Menus" (2023) (97); Wholesome Crave (2023) (66)

University of Wisconsin-Oshkosh	- **Meatless Monday:** Participation in the "Meatless Monday" program through the expansion of vegetarian menu options	"Meatless Mondays Modification" (2014) (263)
University of Wyoming	- Expansion of plant-based menu options at Washakie Dining Center and express counters	Victor (2018) (264)
Vanderbilt University	- **The Purpose-Driven Plant-Based Incubator:** Participation in the Purpose-Driven Plant-Based Incubator program that offers college and university foodservices recipes, workshops, and collaboration to expand plant-forward menu options<br>- Introduction of a kosher and vegetarian dining hall called **Grins Vegetarian Cafe**<br>- Expansion of plant-rich menu options (i.e., grain bowls) and reduction in beef purchases among dining services<br>- Reduction of beef menu options and increase of seafood and vegetarian menu options	AASHE (n.d.) (265); Cobe (2016) (220); Gruber (2015) (55); "Nestlé Professional, Wholesome Crave and UMass Dining Launch Purpose-Driven Plant-Based Incubator™ to Accelerate Shift in College and University Menus" (2023) (97); Vanderbilt University (n.d.) (266); Wholesome Crave (2023) (66)
Villanova University	- **Meatless Monday:** Participation in the "Meatless Monday" program through special offerings of meatless menu options on Monday in every dining hall<br>- Support of dining program's four pillars: 1) Make Fruit and Vegetables the Center of Your Plate; 2) Increase Water & Reduce Sugary Beverage Intake; 3) Increase Your Whole Grain Intake; and 4) Decrease Your Animal Protein Intake through the expansion of plant-based menu options<br>- Expansion of plant-based menu options	Lehmkuhl (2016) (267); Stoessel (2014) (268); Villanova University Sustainability (n.d.) (269)

Virginia Polytechnic Institute and State University (Virginia Tech)	- Expansion of plant-based, vegan, and vegetarian menu options	AASHE (n.d.) (270); Buzalka (2018) (271); "Growing the edge of plant-forward campus dining" (2022) (272)
Wake Forest University	- Expansion of plant-rich menu options through shift to a plant-forward dining focus and prioritizing the development of creative and diverse plant-forward recipes	McMullen (2022) (273); Wake Forest University (n.d.) (274); Wake Forest University (n.d.) (275)
Washington State University	- **Forward Food Pledge:** Commitment to transition at least 33% of meat-based entrees to plant-based entrees annually through the end of 2027<br>- Commitment to have 40% of menu options be plant-based by 2025<br>- Introduction of new plant-based recipes or products	ForwardFood (2020) (4); Humane World for Animals (2025) (37)
Wellesley College	- Introduction of new recipes that align with a plant-rich dietary pattern	Fitzpatrick (2012) (116)
Western Oregon University	- **Forward Food Pledge:** Commitment to increase plant-based menu options to 50% by 2027 and commitment to transition at least 33% of meat-based entrees to plant-based entrees annually through the end of 2027	ForwardFood (2020) (4); "Humane Society: Western Oregon University Pledges to Serve 50% Plant-Based Meals on Campus by 2027" (2024) (276)
Williams College	- **Coolfood Pledge:** Commitment to a target of reducing food related greenhouse gas emissions by 25% by 2030 relative to a 2015 baseline<br>- **Plant-Rich Mondays:** Initiative to expand plant-rich menu options<br>- **Meatless Mondays:** Participation in the “Meatless Monday” program	AASHE (n.d.) (277); Coolfood (n.d.) (34); Evans (2024) (278);

		Williams College (2024) (279); Williams College (n.d.) (280)
Yale University	- Commitment to increase plant-based options by 20% in the next three years<br>- Expansion of plant-rich menu options through vegan desserts and meatless menu options<br>- Expansion of plant-rich menu options through offering of plant-based meat alternatives through partnerships with Beyond Meat and Nestlé<br>- Description of timeline and strategic initiatives including the commitment to decrease animal protein by 15% (2009-2013) decrease animal protein by 5% (2013-2016), and increase plant-forward menu option to 80% of offerings (2016)	AASHE (n.d.) (281); Cobe (2016) (220); King (2013); "Nestle Professional and Sweet Earth® Partner with University of Massachusetts, Yale, Notre Dame and Other U.S. Colleges to Expand Innovative Plant-Based Dining Options - Universities are meeting increased student demand for plant-based food with Bac'n Cheezeburger and other culinary offerings for World Vegan Month" (2020) (282); Rebecchi (2017) (283); Yale Hospitality (n.d.) (284)

Supplemental Table 3. Portion Strategies Used by Higher Education Institutions to Encourage Customers to Select Plant-Rich Menu Options

**Portion Strategies Used by Higher Education Institutions to Encourage Customers to Select Plant-Rich Menu Options (*n*=26)**		
**Name of Higher Education Institution**	**Summary of MMCA Strategy** (Name of Program, Commitment, or Policy bolded if applicable)	**Evidence Source(s)**
Central Washington University	- Reduction of the portion of animal proteins within menu options	Buzalka (2021) (285)
Columbia University in the City of New York	- Increasing the portion of plant-based menu items through a target for 50% of items in every meal to be plant-based	Columbia University (n.d.) (60)
Cornell University	- Description of using mushrooms as a meat altercative on campus and smash burgers composed of a blend of 40% mushrooms that come in a variety of flavors (i.e., gyro and Southwest jalapeno) on a bed of arugula	Gingerella (2017) (286); The Menus of Change University Research Collaborative (n.d.) (287)
Johnson & Wales University (North Miami campus)	- Description of menu options starting out as vegan while meat and dairy are added in moderation	Sentenac (2015) (93)
Michigan State University	- Description of making protein an accent on the plate and allowing guests control over portions of protein (discussed in the context of plant proteins)<br>- Reduction of animal protein portions when creating recipes and menus	Cobe (2016) (220); Michigan State University Division of Residential and Hospitality Services (2021) (104)
Northeastern University	- Introduction of a planetary plate in some dining halls consisting of half vegetables and meat as a garnish	McBride (2019) (67)

Oregon State University	- **The Protein Flip:** Participation in The Protein Flip through the Menus of Change University Research Collaborative<br>- Reduction of the portion size of animal proteins served	Humane World for Animals (2025) (37); The Menus of Change University Research Collaborative (n.d.) (287)
Seattle University	- Featuring vegetables and starches as the center of the plate with a smaller emphasis and size of the traditional animal proteins at the **"Thrive without 9"** allergen-friendly station	AASHE (n.d.) (142)
Stanford University	- **The Protein Flip:** Participation in The Protein Flip through the Menus of Change University Research Collaborative<br>- Focusing on dishes using ground meat that could be blended with vegetables through a ratio of 60% vegetables and 40% meat<br>- Development of mixed dishes with meat and vegetable blend with at least 30% plant-based ingredients by default<br>- Development of blended burgers that consist of animal sourced foods (i.e., beef) and plant sourced foods (i.e., mushrooms)<br>- Use of 30% mushroom blend in all beef burgers<br>- Us of no more than 3 ounces of beef, pork, or poultry per seving<br>- Use of humanely raised meat and poultry in smaller portions and use of bone-in cuts that promote mindful consumption	Residential & Dining Enterprises Stanford Dining, Hospitality & Auxiliaries (n.d.) (152); The Menus of Change University Research Collaborative (n.d.) (88); The Menus of Change University Research Collaborative (n.d.) (287)
The Pennsylvania State University	- Reduction of burger patties offered through Residential Dining grill areas by 1 ounce to encourage a healthy meat portion	AASHE (n.d.) (166)
The University of Arizona	- Reduction of the portion size of animal proteins served<br>- Development of blended burgers and meatballs that consist of animal sourced foods and plant sourced foods (i.e., mushrooms)	Humane World for Animals (2025) (37)

The University of Texas at Austin	- Reduction of the portion size of animal proteins served	Humane World for Animals (2025) (37)
University of California, Berkeley	- Reduction of the portion size of animal proteins served with the aim to increase the ratio of plant-based foods on every plate	Humane World for Animals (2025) (37); UC Berkeley Dining (n.d.) (288)
University of California, Davis	- Reduction of the portion size of animal proteins served such as the 50/50 meat and mushroom blend burger	AASHE (n.d.) (187); Humane World for Animals (2025) (37)
University of California, Los Angeles	- Reduction of the portion size of animal proteins served<br>- Development of a blended burger consisting of 70% pasture-raised beef, 10% mushrooms, 10% onions, 5% quinoa, and 5% beets with the goal of dining services is to eventually reduce the beef content to 60%	Humane World for Animals (2025) (37); Cleveland and Jay (2020) (194)
University of California, Riverside	- **The Protein Flip:** Participation in The Protein Flip through the Menus of Change University Research Collaborative<br>- Utilizing meat as a condiment while using grains and vegetables as the main component of menu options	The Menus of Change University Research Collaborative (n.d.) (287)
University of California, San Diego	- Reduction of the portion size of animal proteins served	Humane World for Animals (2025) (37)
University of Colorado Boulder	- **The Protein Flip:** Participation in The Protein Flip through the Menus of Change University Research Collaborative<br>- Reduction of the portion size of animal proteins served	Humane World for Animals (2025) (37); The Menus of Change University Research

		Collaborative (n.d.) (287)
University of Georgia	- Reduction of the portion size of animal proteins served	Humane World for Animals (2025) (37)
University of Maryland, College Park	- Reduction of the portion size of animal proteins served	Humane World for Animals (2025) (37)
University of New Hampshire	- **The Protein Flip:** Participation in The Protein Flip through the Menus of Change University Research Collaborative	The Menus of Change University Research Collaborative (n.d.) (287)
University of North Texas	- Reduction of the portion size of animal proteins served	Humane World for Animals (2025) (37)
University of Southern California	- **The Protein Flip:** Participation in The Protein Flip through the Menus of Change University Research Collaborative<br>- Reduction of the portion size of animal proteins served<br>- Reduction of animal proteins to no more than 4 ounces per serving across some food retail stations<br>- Reduction of animal proteins through decreased portion sizes of animal-sourced foods served by staff to students	AASHE (n.d.) (255); Humane World for Animals (2025) (37); The Menus of Change University Research Collaborative (n.d.) (287)
Vanderbilt University	- Reduction of the portion size of animal-sourced menu options through all burgers consisting of the beef and mushroom blend (i.e., 25% mushrooms and 75% beef) across campus	AASHE (n.d.) (266); Vanderbilt University (n.d.) (266)
Villanova University	- Reduction of the portion size of animal-sourced ingredients due to a switch to lower-fat hamburger patties made with a ground beef and mushroom blend	Steele (2017) (289)

Wake Forest University	- **The Mushroom Burger:** Reduction of beef used for hamburgers and cheeseburger through a 30% mushroom and 70% beef blend<br>- Use of plant-based defaults through the implementation of a "plant first" structure as the default in dining halls and catering services in which plant-based foods are placed at the beginning of the line and animal-based foods are served in smaller portions at the end of the line	Wake Forest University (n.d.) (274); Wake Forest University (n.d.) (275)

Supplemental Table 4. Pricing Strategies Used by Higher Education Institutions to Encourage Customers to Select Plant-Rich Menu Options

**Pricing Strategies Used by Higher Education Institutions to Encourage Customers to Select Plant-Rich Menu Options (*n*=9)**		
**Name of Higher Education Institution**	**Summary of MMCA Strategy** (Name of Program, Commitment, or Policy bolded if applicable)	**Evidence Source(s)**
Canisius University (formerly Canisius College)	- Implementation of price incentives at plant-based retail station, **Pitchforks**, in which plant-based meals were priced fifty cents less than meat-based meals	Middleton and Littler (2019) (5)
Florida State University	- **Meatless Monday:** Participation in the "Meatless Monday" program by offering discounts offered on meatless dishes on Mondays at some location on campus by providing 1$ off meals for students who choose to participate in Meatless Monday	Florida State University (2018) (290); "How to eat vegetarian on campus" (2018) (291); Meatless Monday (n.d.) (170)
The Ohio State University	- Introduction of a variety of plant-rich menu options through a new on campus food truck, **Thyme & Change 2.0**, with a vegan menu that features plant-rich counterparts to animal-sourced options at a comparable price	Buzalka (2019) (161)
The University of Arizona	- **$5 Fridays:** Implementation of $5 Fridays at the plant-forward dining hall to promote plant-based menu options	Humane World for Animals (2025) (37)
University of California, Irvine	- **Meatless Monday:** Participation in the "Meatless Monday" program by offering discounts on meatless items at retail locations on Mondays	Middleton and Littler (2019) (5)
University of California, San Diego	- Removal of surcharge for plant-based milks at coffee bars	Humane World for Animals (2025) (37)
University of South Florida	- Discount coupons for meat-free options on Mondays	"University of South Florida in the Running for Most Vegan

		Friendly College in the U.S." (2012) (254)
University of Southern California	- Distribution of free vegan meals at **Jubilee Vegan Café** once a week to students, faculty, and staff that volunteer to cook the meals once a semester	Buzalka (2022) (292); Mendoza (2022) (293); Yeung (n.d.) (294)
Washington State University	- Use of plant-based defaults with the option to add meat and dairy for a surcharge within one dining hall or station	Humane World for Animals (2025) (37)

Supplemental Table 5. Promotion Strategies Used by Higher Education Institutions to Encourage Customers to Select Plant-Rich Menu Options

**Promotion Strategies Used by Higher Education Institutions to Encourage Customers to Select Plant-Rich Menu Options (*n*=66)**		
**Name of Higher Education Institution**	**Summary of MMCA Strategy** (Name of Program, Commitment, or Policy bolded if applicable)	**Evidence Source(s)**
American University	- **Meatless Monday:** Promotion of the "Meatless Monday" program by featuring vegan and vegetarian specials at each station on Mondays and using marketing materials	Middleton and Littler (2019) (5)
Binghamton University	- Promotion of plant-rich menu options using a vegan/vegetarian dining guide available online and in paper format to guide students toward plant-based options across campus	AASHE (n.d.) (20); Binghamton University Dining Services (2024) (295)
Boston University	- Promotion of plant-rich menu options (i.e., vegan and vegetarian) using marketing materials such as digital signs, large format banners, and posters	AASHE (n.d.) (25)
Brigham Young University	- Use of marketing campaigns focused on plant-based initiatives<br>- **Eat, Act, Think:** Marketing campaign that consists of signage, digital billboards and tabletop advertisements to educate students on low-impact food choices	Humane World for Animals (2025) (37)
Bucknell University	- Promotion of plant-rich Mediterranean menu options through flags from various countries and examples of the dishes available	Kiernan (2010) (41)
Central Washington University	- Promotion of plant-based menu options through a series of Instagram posts on CWUEats	AASHE (n.d.) (47)
Clark University	- Promotion and showcase of vegan and vegetarian options through social media and promotion of vegan station in a promotional video to help students navigate the dining hall	Chisholm (2019) (49)
Colgate University	- Promotion of a plant-rich dietary pattern through the "**Be A Flexitarian**" initiative that encourages customers to eat no meat at least once a week using posters and signs on campus	AASHE (n.d.) (296); Colgate University Dining Services (n.d.) (52)

College of Charleston	- Advertisement of vegan, vegetarian, and plant-based menus and events online and in person	AASHE (n.d.) (54)
College of the Holy Cross	- Promotion and showcase of vegan and vegetarian options through social media	Chisholm (2019) (49)
Colorado State University	- Use of marketing campaigns focused on plant-based initiatives and promotion of plant-based menu options through social media and handouts through the **Environmental Eats** program	AASHE (n.d.) (57); Humane World for Animals (2025) (37)
Duke University	- **Meatless Monday:** Promotion of the "Meatless Monday" program and vegan menu options through videos posted online	Palmisano (2015) (72)
Florida State University	- Promotion of vegan menu options through educational materials with the objective of informing students about how and where to eat vegan menu options at retail locations	AASHE (n.d.) (297)
Georgia State University	- Use of marketing campaigns focused on plant-based initiatives and promotion of plant-based menu items (i.e., social media campaigns and signage)	Humane World for Animals (2025) (37)
Indiana University Bloomington	- Use of marketing and educational campaigns focused on plant-based initiatives (i.e., encouragement of meat-free and low-meat entrees)	AASHE (n.d.) (298); Humane World for Animals (2025) (37)
Ithaca College	- Use of signage to educate customers about the benefits of plant-based dietary patterns, the environmental implication of animal products, food waste, and composting	AASHE (n.d.) (89)
Johns Hopkins University	- **Meatless Monday:** Promotion of food options that are healthy for people and the environment through the "Meatless Monday" campaign to encourage students to examine the environmental impact of their health choices	"ARAMARK Joins Client Partners to Present at Advancement of Sustainability in Higher Education (AASHE) Conference -

		Colleges and Universities to share campus sustainability best practices" (2011) (299)
Montclair State University	- Educating students about plant proteins through signage	Cobe (2019) (105)
Muhlenberg College	- **Meatless Monday:** Features of plant-forward options through the "Meatless Monday" program and **Plant-Based Take-over Days**	AASHE (n.d.) (300)
New York University	- Promotion of vegan and vegetarian menu options through **Weekly Vegetarian Nights** and **Monthly All Vegan Meals**<br>- Use of Instagram to highlight plant-forward menu options	AASHE (n.d.) (107); "U of F rated in top ten of best colleges in country for vegetarians" (2011) (301)
North Carolina State University	- Use of marketing campaigns focused on plant-based initiatives and promotion of plant-based menu options through social media and dining hall display screens<br>- Promotion of plant-forward menu options through "**Plant-Powered Days**" each semester	Humane World for Animals (2025) (37)
Northern Arizona University	- Promotion and showcase of plant-based menu options though "plant-based takeovers"<br>- Promotion of plant-based menu options on campus through "Plant Based Dining on Campus" guide and map of "most popular plant-based options" on campus	AASHE (n.d.) (114); Northern Arizona University (n.d.) (302)
Northwestern University	- **Meatless Monday:** Participation in the "Meatless Monday" program as a promotional effort to encourage customers to choose a non-meat entrees one day per week	"Northwestern tops list of most vegan-friendly colleges" (2011) (119)
Ohio University	- Promotion of plant-forward meals through partnership with football team, including social media videos in which chefs and players cook together	The Menus of Change University

		Research Collaborative and Stanford Food Institute Residential & Dining Enterprises (2024) (121)
Oregon State University	- Use of marketing campaigns focused on plant-based initiatives<br>- Promotion of vegetarian and vegan menu options through menu guides	Humane World for Animals (2025) (37); Oregon State University (n.d.) (123)
Quinnipiac University	- **Meatless Monday:** Participation in the "Meatless Monday" program as a promotional effort to encourage customers to choose a non-meat entrees one day per week through promotional and informational messages on social media such as "Cut Broccoli, not rainforests. Protect the rainforests from becoming land for grazing cattle. Eat more plant-based meals. Good for you. Good for the planet." and "Save water. Producing ¼ pound of hamburger requires an astounding 449 gallons of water. Preserve our water sources."	Buzalka (2019) (126)
Rice University	- Promotion of a variety plant-based menu options through marketing tools, such as the Instagram hashtag #ourplantbasedjourney	Vaughn (2018) (127)
Rutgers, The State University of New Jersey (Rutgers University)	- Use of marketing campaigns focused on low carbon footprint and plant-based initiatives	Humane World for Animals (2025) (37); Rutgers Dining Services (n.d.) (133)
Salisbury University	- **Meatless Monday:** Promotion of vegan menu options through the "Meatless Monday" campaign<br>- Advertisements of vegan menu options	AASHE (n.d.) (303); "Salisbury university dining services earns a on peta2 vegan

		report card" (2016) (135); "Salisbury university dining services earns a on peta2 vegan report card" (2017) (136)
San Diego State University	- Publication of a campus vegan guide that outlines plant-based options on campus	Humane World for Animals (2025) (37)
Stanford University	- Use of taste-focused language in all menus to reframe healthy and plant-forward menu options with flavorful, fun, and delicious language<br>- Use of "dynamic norms" to highlight the popularity of plant-based foods	Residential & Dining Enterprises Stanford Dining, Hospitality & Auxiliaries (n.d.) (152)
Stony Brook University	- Promotion of **Plant-based Eating Road Map** through email, social media, and website's wellness section	Buzalka (2023) (101); Buzalka (2022) (304)
Syracuse University	- **Meatless Monday:** Participation in the "Meatless Monday" program as a promotional effort by highlighting meatless options as a suggestion for students to try meatless menu options to increase awareness that increasing plant-rich foods and eating less meat may prevent chronic disease and encourage customers to try something meatless<br>- Participation in "**Healthy Monday**" and "**Try Me**" promotion of a new grain, fruit, or vegetable each month	David George (2010) (157); King (2013) (158)
The Ohio State University	- Use of marketing campaigns focused on plant-based initiatives	Humane World for Animals (2025) (37)
The Pennsylvania State University	- Promotion of plant-based options through Instagram	AASHE (n.d.) (166)

The University of Arizona	- Use of marketing campaigns focused on plant-based initiatives<br>- Identification of plant-based options on campus through a dining guide	Humane World for Animals (2025) (37)
The University of Iowa	- **Meatless Monday:** Promotion of meatless options available in dining halls through flyers and promotion of the "Meatless Monday" program to encourage students to look at the environmental cost of meat	Miller (2018) (171); "University of Iowa holds first Meatless Monday at dining halls" (2018) (172)
The University of Oklahoma	- Encouraging students to select plant-based menu options by utilizing branded posters, flyers, and other materials that compare the health and environmental impacts of plant and animal proteins	Humane World for Animals (2025) (37)
The University of Texas at Austin	- Use of marketing campaigns focused on plant-based initiatives and use of informational displays through dining locations to highlight plant-based offerings<br>- Education of low impact menu options (i.e., plant-rich) through **Coolfood Meals** signage	AASHE (n.d.) (174); Humane World for Animals (2025) (37)
Towson University	- Promotion of new vegetarian menu options through posters on campus with a woman wearing a dress made of vegetables	Obineme (2013) (179)
University at Albany	- **Meatless Monday:** Participation and promotion of "Meatless Monday" as a promotional effort to encourage plant-based options	AASHE (n.d.) (183)
University at Buffalo	- **Plant-Powered Mondays:** Marketing campaigns focused on ensuring plant-forward menu options are available across campus<br>- **Strictly Vegetarian:** Marketing campaigns focused on ensuring plant-forward menu options are available across campus	Humane World for Animals (2025) (37)
University of California, Berkeley	- Use of marketing campaigns focused on plant-based initiatives	Humane World for Animals (2025) (37)
University of California, Davis	- Use of marketing campaigns focused on plant-based initiatives	Humane World for Animals (2025) (37)

University of California, Los Angeles	- Use of marketing campaigns focused on plant-based initiatives (i.e., Meatless Monday and Beefless Thursdays)<br>- Use of communication campaigns to encourage healthy and sustainable dietary patterns by encouraging plant-forward eating<br>- Encouragement of plant-based menu options through table tents, digital posters, and signage to educate students about the benefits of reducing animal proteins	Humane World for Animals (2025) (37); UCLA Annual Foodservice Sustainability Policy Report (2018–2019) (192)
University of California, Riverside	- **Meatless Monday:** Participation in the "Meatless Monday" program as an educational campaign to encourage customers to eliminate meat from their diets once a week	Plascencia (2013) (200)
University of California, San Diego	- Use of marketing campaigns focused on plant-based initiatives<br>- Promotion of vegan, vegetarian, and sustainable menu items through social media content	AASHE (n.d.) (202); Humane World for Animals (2025) (37)
University of California, Santa Barbara	- Promotion of plant-rich menu options through the **Climate Friendly Monday** campaign that focuses on reducing dining services climate footprint and the reduction of meat and dairy menu options and increasing local, organic, seasonal, and whole food menu options on Mondays twice a quarter	AASHE (n.d.) (207)
University of California, Santa Cruz	- **Meat-Free Monday:** Participation in the "Meat-Free Monday" program as an educational opportunity for students and a promotion of a "different way of eating"	McCord (2010) (204)
University of Colorado Boulder	- Use of marketing campaigns focused on plant-based initiatives	Humane World for Animals (2025) (37)
University of Georgia	- Use of marketing campaigns focused on plant-based initiatives and offerings through posters, social media, and informational displays	Humane World for Animals (2025) (37)
University of Maryland, College Park	- Use of social media and smartphone Apps to share information about the nutrition and sourcing of menu options<br>- Use of dining hall table tents to provide information about sustainable eating and the effects of the foods they are choosing<br>- Use of marketing campaigns focused on plant-based initiatives	Curtis (2019) (221); Humane World for Animals (2025) (37); "World

		Resources Institute: Maryland Becomes the First 'Cool Food' University" (2019) (222)
University of Massachusetts Amherst	- **Sanitas Per Escam ("Health Through Food"):** Promotion of SPE daily menu options through social media and banners along with posters with information about healthy eating	"Third-party Certification Improves Healthy Option Consumption at Umass" (2013) (227)
University of Michigan-Ann Arbor	- Use of marketing campaigns focused on plant-based initiatives	Humane World for Animals (2025) (37)
University of Nebraska-Lincoln	- Use of marketing campaigns focused on plant-based initiatives	Humane World for Animals (2025) (37)
University of New Hampshire	- Promotion of healthy plant proteins through the **Wildcat Plate** and signage of plate model with information on portion control and the guidance to "reduce red meat and choose fish, poultry, beans, nuts and seeds" throughout the campus dining halls	AASHE (n.d.) (305); Gosling (2023) (306); University of New Hampshire (n.d.) (307)
University of North Texas	- Use of marketing campaigns focused on plant-based initiatives and promotion of plant-based options through social media and digital screens	Humane World for Animals (2025) (37)
University of Rochester	- **Meatless Monday:** Description of the "Meatless Monday" program as a campus event	Dowd (2015) (251)

University of Southern California	- **EcoMonday:** Participation in the "EcoMonday" program across three main dining halls where red meat is not served and plant-forward menu options are promoted on social media<br>- Use of marketing campaigns focused on plant-based initiatives<br>- Encouragement of plant-based menu options through educational posters and table tents in dining halls	Humane World for Animals (2025) (37)
University of Virginia	- Promotion of plant-rich menu items using signage throughout the dining halls about a variety of our sustainability initiatives, such as plant-forward menu options on campus, information on Coolfood meals, and an overview of the sustainability program	AASHE (n.d.) (258)
University of Washington	- **Food Literacy Program:** Promotion of new recipes that align with a plant-rich dietary pattern through information cards describing appealing and nutritious facts<br>- Use of marketing campaigns focused on plant-based initiatives	Fitzpatrick (2018) (262); Humane World for Animals (2025) (37)
University of Wisconsin-Madison	- Use of marketing campaigns focused on plant-based initiatives	Humane World for Animals (2025) (37)
University of Wisconsin-Oshkosh	- **Meatless Monday:** Participation in the "**Meatless Monday**" program through the encouragement of meatless options through presentation of the benefits of reducing meat (i.e., statistics and data) on televisions and kiosks	"Meatless Mondays Modification" (2014) (263)
Virginia Polytechnic Institute and State University (Virginia Tech)	- **Plant-Forward Friday:** Use of marketing campaigns focused on plant-based initiatives and the promotion of plant-based menu options through the "**Plant-Forward Friday**" series and signage related to plant-forward meals	AASHE (n.d.) (270); "Growing the edge of plant-forward campus dining" (2022) (272); Humane World for Animals (2025) (37)
Washington State University	- Use of marketing campaigns focused on plant-based initiatives<br>- Promotion of the health and environmental benefits of plant-based foods through posters and signage	Humane World for Animals (2025) (37)

Yale University	- Promotion of meatless menu options through educational materials	AASHE (n.d.) (281)

Supplemental Table 6. Picks Strategies Used by Higher Education Institutions to Encourage Customers to Select Plant-Rich Menu Options

**Picks Strategies Used by Higher Education Institutions to Encourage Customers to Select Plant-Rich Menu Options (*n*=17)**		
**Name of Higher Education Institution**	**Summary of MMCA Strategy** (Name of Program, Commitment, or Policy bolded if applicable)	**Evidence Source(s)**
Brigham Young University	- Use of plant-based defaults with the option to add meat and dairy within one dining hall or station	Humane World for Animals (2025) (37)
Colorado State University	- Use of plant-based defaults with the option to add meat and dairy within one dining hall or station	Humane World for Animals (2025) (37)
Columbia University in the City of New York	- Integration of plant-based foods as the default choice while giving diners the option for meat or dairy	Columbia University in the City of New York (n.d.) (60); de Sousa (2024) (308)
Harvard University	- Use of plant-based meals by default and giving diners the choice to opt-in for meals with animal products during events and meetings	Harvard Office for Sustainability (n.d.) (86)
North Carolina State University	- Use of plant-based defaults with the option to add meat and dairy within one dining hall or station	Humane World for Animals (2025) (37)
Oregon State University	- Use of plant-based defaults with the option to add meat and dairy within one dining hall or station	Humane World for Animals (2025) (37)
Stanford University	- **The Protein Flip:** Use of plant-based defaults that align with Protein Flip recipes or menu options consisting of a blend of ground meat and vegetables through a ratio of 60% vegetables and 40% meat<br>- Use of plant-based defaults (salads, grain bowls, and pastas) with meat, poultry, egg, or cheese offered on the side of by request as optional additions	Residential & Dining Enterprises Stanford Dining,

		Hospitality & Auxiliaries (n.d.) (152); The Menus of Change University Research Collaborative (n.d.) (88)
The Ohio State University	- Use of plant-based defaults with the option to add meat and dairy within one dining hall or station	Humane World for Animals (2025) (37)
The University of Arizona	- Use of plant-based defaults with the option to add meat and dairy within one dining hall or station	Humane World for Animals (2025) (37)
University of California, Berkeley	- Use of plant-based defaults with the option to add meat and dairy within one dining hall or station	Humane World for Animals (2025) (37)
University of California, Davis	- Use of plant-based defaults with the option to add meat and dairy within one dining hall or station	Humane World for Animals (2025) (37)
University of California, San Diego	- Use of plant-based defaults with the option to add meat and dairy within one dining hall or station	Humane World for Animals (2025) (37)
University of Colorado Boulder	- Use of plant-based defaults with the option to add meat and dairy within one dining hall or station	Humane World for Animals (2025) (37)
University of Georgia	- Use of plant-based defaults with the option to add meat and dairy within one dining hall or station	Humane World for Animals (2025) (37)
University of Michigan-Ann Arbor	- Use of plant-based defaults with the option to add meat and dairy within one dining hall or station	Humane World for Animals (2025) (37);

	- Introduction of oat milk as the default choice for milk-based beverages within café location in Fall 2024	Michigan Dining (n.d.) (235)
University of Southern California	- Use of plant-based defaults with the option to add meat and dairy within one dining hall or station	Humane World for Animals (2025) (37)
Washington State University	- Use of plant-based defaults with the option to add meat and dairy for a surcharge within one dining hall or station	Humane World for Animals (2025) (37)

Supplemental Table 7. Priming/Prompting Strategies Used by Higher Education Institutions to Encourage Customers to Select Plant-Rich Menu Options

**Priming/Prompting Strategies Used by Higher Education Institutions to Encourage Customers to Select Plant-Rich Menu Options (*n*=75)**		
**Name of Higher Education Institution**	**Summary of MMCA Strategy** (Name of Program, Commitment, or Policy bolded if applicable)	**Evidence Source(s)**
Arizona State University	- Identification of low carbon footprint and low impact menu options (i.e., plant-rich) through **Coolfood Meals** icons on menu boards to inform customers of low impact foods	AASHE (n.d.) (10)
Bentley University	- Labeling of vegan and vegetarian menu options using symbols on menus	AASHE (n.d.) (16)
Binghamton University	- Labeling of vegan, vegetarian, and plant-based options through an icon system available at resident dining halls each day	Binghamton University Dining Services (2024) (295); Gottlieb and Roazzi (2013) (309)
Boston University	- Labeling of vegan and vegetarian menu items through an icon system on menus<br>- Identification of low carbon footprint menu options (i.e., plant-rich) through **Coolfood Meals** program and **Coolfood** badge on the menus	AASHE (n.d.) (25); Boston University Dining Services (n.d.) (26); Boston University Dining Services (2023-2024) (27)
Bowdoin College	- Labeling of vegan and vegetarian menu options in the dining hall	AASHE (n.d.) (29)
Brandeis University	- Labeling of vegan and vegetarian menu options through an icon system on digital menu boards and dining website	Brandeis Hospitality (n.d.) (310);

	- Identification of low carbon footprint menu options (i.e., plant-rich) on digital menu boards and dining website through **Coolfood Meals** program and badges on the menus	Brandeis Hospitality (n.d.) (35)
Brigham Young University	- **Eat, Act, Think:** Marketing campaign that consists of labeling to educate students on low-impact food choices	Humane World for Animals (2025) (37)
Bryn Mawr College	- Labeling of vegan and vegetarian menu options through indicators on menus and labels	Bryn Mawr College (n.d.) (311)
Carnegie Mellon University	- Identification of vegan and vegetarian menu options at select dining locations	AASHE (n.d.) (45)
Central Washington University	- Labeling of vegan and vegetarian menu options through an icon system on menus available throughout Dining Services	Buzalka (2019) (48); Central Washington University (n.d.) (312)
College of Charleston	- Labeling of vegan, vegetarian, and plant-forward menu items through an icon system that make better-for-you choices easy to find	College of Charleston (n.d.) (313)
Colorado State University	- Labeling of vegan and vegetarian menu options through an icon system added to menu options at dining centers and online	AASHE (n.d) (57); Colorado State University (n.d.) (314)
Columbia University in the City of New York	- Labeling of vegan and vegetarian menu options through an icon system at all dining locations	Columbia University (n.d.) (315)
Drexel University	- Identification of plant-based menu options through "Eat Well" leaf symbols	Taylor (2019) (71)
Florida State University	- Identification of low impact menu options (i.e., plant-rich) through **Coolfood Meals** program and menu icon throughout Seminole Dining	AASHE (n.d.) (297); Florida State University (n.d.) (78)

Framingham State University	- Identification of vegetarian menu options with icon	Framingham State University Dining Services (n.d.) (316)
Georgia Institute of Technology (Georgia Tech)	- Labeling of vegan menu options at every dining location	AASHE (n.d.) (80)
Indiana State University	- Labeling of vegan and vegetarian menu options at one food retailer called Generations Restaurant	Rojas (2014) (317)
Indiana University Bloomington	- Labeling of vegan and vegetarian menu options through menu icon system at dining locations and online	AASHE (n.d.) (318); Indiana University (n.d.) (319); "U of F rated in top ten of best colleges in country for vegetarians" (2011) (301)
Johns Hopkins University	- Labeling of vegan and vegetarian menu options through an icon system	Johns Hopkins University (n.d.) (320)
Loyola University New Orleans	- Identification of vegan and vegetarian menu options at the Orleans Room	Killett (2021) (321)
Marquette University	- Labeling of vegan menu options	“From Tikka Masala to Congee, Campus Dining Trends Go Beyond Chicken Tenders and Pizza - Sodexo,

		Which Runs Dining Services at More Than 600 U.S. Colleges and Universities, Names Top Dining Trends" (2018) (322)
Michigan State University	- Labeling of vegan and vegetarian menu options through an icon system at the point of services and online	Michigan State University (n.d.) (323); Michigan State University Division of Residential and Hospitality Services (2021) (104)
Muhlenberg College	- Labeling of vegan and vegetarian menu options through an icon system at new campus restaurant and on menu screens	AASHE (n.d.) (300); "Everything's Kosher at New Muhlenberg Facility" (2010) (324)
New York University	- Labeling of vegan and vegetarian menu options through an icon system above the menu items and on digital screens across all NYU Eats locations	AASHE (n.d.) (107)
North Carolina State University	- Labeling of vegan and vegetarian menu items through an icon system in dining locations and online	AASHE (n.d.) (110); NC State Dining (n.d.) (325)

Northeastern University	- Labeling of vegan and vegetarian menu items through an icon system on menus through Balanced U program that provides clear signage and labeling	AASHE (n.d.) (112); Northeastern University Dining (n.d.) (326)
Northern Arizona University	- Labeling of vegan and vegetarian menu items through an icon system on menus	AASHE (n.d.) (114); Northern Arizona University (n.d.) (302)
Northern Kentucky University	- Labeling vegan and vegetarian menu options through the **Healthy U** policy	Enzweiler (2015) (327)
Northern Michigan University	- Labeling of vegan and vegetarian menu options through an icon system at Northen Lights Dining	AASHE (n.d.) (115)
Oregon State University	- Labeling of vegetarian and vegan menu options through an icon system on menus and use of menu guides describing vegetarian and vegan options	Oregon State University (n.d.) (328); Oregon State University (n.d.) (123)
Salisbury University	- Labeling of vegan menu options in dining halls	AASHE (n.d.) (303); "Salisbury university dining services earns a on peta2 vegan report card" (2016) (135); "Salisbury university dining services

		earns a on peta2 vegan report card" (2017) (136)
San Diego State University	- Labeling of vegan menu options at every dining location and publication of a campus vegan guide that outlines plant-based options on campus	AASHE (n.d.) (137); Humane World for Animals (2025) (37)
Seattle University	- Labeling vegan and vegetarian menu options through an icon system	AASHE (n.d.) (142)
Skidmore College	- Listing menu items that are vegan and vegetarian and labeling vegan and vegetarian menu options	Allergen Friendly Skidmore (n.d.) (329); “Skidmore Eat Healthy Strives to Improve Wellness” (2012) (330)
Stanford University	- Labeling of vegan and vegetarian menu options	Residential & Dining Enterprises Stanford Dining, Hospitality & Auxiliaries (n.d.) (152); Stanford Dining, Hospitality & Auxiliaries Residential &

		Dining Enterprises (n.d.a) (154)
Stony Brook University	- Identification of plant-rich menu options through signage indicating "I'm Plant-Based"	Buzalka (2023) (101); Buzalka (2022) (304)
Syracuse University	- **Meatless Monday:** Identification of Meatless Monday meal options in dining halls and cafes by adding Meatless Monday sticker and logo at the serving stations throughout campus<br>- Labeling of vegan and vegetarian menu options	AASHE (n.d.) (156); King (2013) (158); Syracuse University Maxwell School of Citizenship & Public Affairs (n.d.) (331)
The Ohio State University	- Labeling of vegan and plant-based menu options next to each of the menu items at **Thyme & Change 2.0** and campus dining location across campus	Cagle (2019) (162)
The Pennsylvania State University	- Labeling of vegan and meatless menu options through an icon system	AASHE (n.d.) (166)
The University of Iowa	- Labeling of vegan menu options on 4"x 6" sign/menu cards	AASHE (n.d.) (332)
The University of Utah	- Labeling of vegan menu options through the **Balanced U** icon system	AASHE (n.d.) (177)
University at Albany	- Labeling of vegan and vegetarian menu options	AASHE (n.d.) (183)
University at Buffalo	- Labeling of vegan, vegetarian, low-impact, medium-impact, and high-impact menu options through an icon system	University at Buffalo (n.d.) (333); University at Buffalo (n.d.) (334); University at

		Buffalo (n.d.) (335)
University of California, Berkeley	- Identification of vegan menu options through extensive signage<br>- Identification of low carbon (i.e., plant-rich) menu options on online menus using a stoplight logo system with items marked in red being a high-impact food compared to items marked in green being a low-impact food (i.e., plant forward)	Fitzpatrick (2010) (336); Humane World for Animals (2025) (37); UC Berkeley Dining (n.d.) (337); UC Berkeley Dining (n.d.) (288)
University of California, Davis	- Labeling of vegan menu options on menus	AASHE (n.d.) (187)
University of California, Irvine	- Labeling of vegan menu options using green logos and identification of low impact menu options (i.e., plant-rich) through **Coolfood Meals** labeling on digital boards	AASHE (n.d.) (189)
University of California, Los Angeles	- Identification of plant-rich menu options through information about the carbon footprint of food in the dining halls, and high- and low-carbon-footprint icons added to online and in person menus<br>- Green low carbon footprint icons and Earth symbols added to items with 0-25% of the % Daily Value Dietary Carbon Footprint (i.e., vegan menu items, some vegetarian menu items, and certified sustainable fish) and orange-red high carbon footprint icons and Earth symbols are added to items with more than 50% of the % Daily Value Dietary Carbon Footprint (i.e., beef, lamb, bison, and menu items with more than 3.5 ounces of cheese) aligned with the EAT *Lancet* Commission<br>- Labeling of vegetarian section on some menus and labeling of vegan and vegetarian menu options	AASHE (n.d.) (190); Humane World for Animals (2025) (37); Franchini et al. (2023) (338); The Menus of Change University Research Collaborative (n.d.) (73); "The Veg Heads: On-campus vegan

		food" (2013) (339); UCLA Dining (n.d.) (340)
University of California, San Diego	- Identification of plant-rich menu options at OceanView food retailer through the designation of vegetarian entrees on print and online menus<br>- Identification of vegetarian, vegan, and sustainable options with specific icons on dining menus	AASHE (n.d.) (202); Boss (2017) (341)
University of Central Florida	- Labeling of vegan and vegetarian menu options using brightly colored leaf-shaped messages on dining hall food options to signify selections	AASHE (n.d.) (342)
University of Colorado Boulder	- Labeling of vegan and lacto-ovo vegetarian menu options in all dining operations	University of Colorado Boulder (n.d.) (343)
University of Colorado Colorado Springs	- Labeling of vegan menu options using a vegan symbol on food cards	AASHE (n.d.) (344)
University of Hawaii at Mānoa	- Labeling of vegan menu options	"Peta2 Recognizes Sodexo at the University of Hawaii at Manoa for Vegan Cuisine" (2018) (218)
University of Illinois at Urbana-Champaign	- Labeling of vegan and vegetarian menu options at the point-of-service	AASHE (n.d.) (219)
University of Maine at Presque Isle	- Labeling of vegan and vegetarian menu options through an icon system	University of Maine at Presque Isle (n.d.) (345)
University of Maryland, College Park	- Use of dining hall menus to provide information about sustainable eating and the effects of the foods they are choosing	Curtis (2019) (221)

University of Massachusetts Amherst	- Labeling of the carbon footprint of menu options through UMass Dining Carbon Rating System that categorizes menu items from A-E through partnership with My Emissions and the Dining for a **Cooler Planet** campaign<br>- Labeling of vegetarian and plant-based menu icons included in the menu identifiers	AASHE (n.d.) (346); UMass Amherst (n.d.) (347)
University of Miami	- Labeling of vegan and vegetarian menu options on the menu and dining website through an icon system	AASHE (n.d.) (348); "Vegan-friendly options increasing in campus dining halls" (2018) (230); University of Miami Dining (n.d.) (231)
University of Michigan-Ann Arbor	- Labeling of vegan, vegetarian, and low, high, and medium carbon footprint menu items at the point-of-selection through an icon system	AASHE (n.d.) (349); Michigan Dining (n.d.) (235); Michigan Dining (n.d.) (350); Michigan Dining (n.d.) (351)
University of Minnesota Duluth	- Labeling of vegan and vegetarian menu options through an icon system and digital labels	AASHE (n.d.) (352)
University of New Hampshire	- Indication of vegan and vegetarian menu options through prominent menu signage	AASHE (n.d.) (305)
University of Notre Dame	- Labeling of vegan and vegetarian menu options through an icon system on electronic menu boards and online	University of Notre Dame (n.d.) (353); University of

		Notre Dame (n.d.) (354)
University of Oregon	- **iNutrition:** Identification of vegan/vegetarian options through iNutrition application<br>- Encouragement of food choices that are good for the planet by marking menu options that meet low carbon footprint criteria (i.e., plant-rich) across campus dining venues	AASHE (n.d.) (243); "App-etizing Dining Programs" (2012) (355); University of Oregon (n.d.) (356)
University of Pennsylvania	- Labeling of vegan and vegetarian menu options through icons prominently displayed on menus	AASHE (n.d.) (245); Nguyen (2010) (357)
University of Pittsburgh	- Labeling of vegan and vegetarian menu options in dining halls	AASHE (n.d.) (247); "From Tikka Masala to Congee, Campus Dining Trends Go Beyond Chicken Tenders and Pizza - Sodexo, Which Runs Dining Services at More Than 600 U.S. Colleges and Universities, Names Top Dining Trends" (2018) (322)

University of San Diego	- Labeling of vegan and vegetarian menu options through an icon system and digital screens	AASHE (n.d.) (252); University of San Diego (n.d.) (358)
University of Southern California	- Labeling of vegetarian and vegan menu options to inform students about low impact food choices	AASHE (n.d.) (255)
University of Vermont	- Identification of vegan, vegetarian, and plant-based menu options (i.e., leaf logo) through online, menus, printed signage, and labeling	AASHE (n.d.) (359); Baxley (2021) (256); University of Vermont (n.d.) (360); University of Vermont (n.d.) (257)
University of Virginia	- Labeling of vegan, vegetarian, and plant-forward menu options and use of **Coolfood** logos to label low carbon (i.e., plant-rich) menu options	AASHE (n.d.) (258); UVA Dine (n.d.) (260); UVA Sustainable Food Collaborative, A Task Force of the UVA Environmental Stewardship Subcommittee on Sustainability (2021-2030) (261)

University of Washington	- **Balance Plate Initiative:** Encouragement of plant-based menu options through information on menu boards	Humane World for Animals (2025) (37)
Vanderbilt University	- Labeling of vegan and vegetarian menu options through an icon system at the point-of-service	AASHE (n.d.) (265)
Villanova University	- Identification of vegan menu options on menus and online without using the word "vegan" and listing ingredients and vegan attributes under the menu options title	Steele (2017) (289); Stoessel (2014) (268)
Wake Forest University	- Identification of low carbon footprint menu options (i.e., plant-rich) through **Coolfood Meals** program and **Coolfood** badge/icon on the menus	Wake Forest University (n.d.) (275)
Washington State University	- Labeling of vegan and vegetarian menu options through an icon system	Washington State University (n.d.) (361)
Yale University	- Labeling of plant-based menu items in every dining hall	AASHE (n.d.) (281)

Supplemental Table 8. Proximity Strategies Used by Higher Education Institutions to Encourage Customers to Select Plant-Rich Menu Options

**Proximity Strategies Used by Higher Education Institutions to Encourage Customers to Select Plant-Rich Menu Options (*n*=10)**		
**Name of Higher Education Institution**	**Summary of MMCA Strategy** (Name of Program, Commitment, or Policy bolded if applicable)	**Evidence Source(s)**
Alma College	- Easy to locate vegan and vegetarian options at each dining station in Hamilton	Alma College (2022) (362)
Ithaca College	- Utilization of strategies to increase customers consumption of plant-based items such as displaying plant-based menu options at more prominent positions than before	AASHE (n.d.) (89); "Ithaca College Dining Services Recognized for Outstanding Plant-based Menu Options" (2018) (90)
New York University	- Normalizing vegan and vegetarian alternative options by making them easy to find by putting them right next to their meat and dairy counterparts	New York University (2020) (109)
Northern Arizona University	- Easy to location vegan and vegetarian options at the **Plant Forward** station with the intention of customers seeing whole foods first	Buzalka (2018) (363)
Seattle University	- Leading with "Veg-forward" vegan options that come above animal protein to encourage vegan options in all menus in **C-street** cafeteria and catering menu	AASHE (n.d.) (142)
Stanford University	- Utilization of choice architecture, behavioral economics, and "healthy placement" to encourage students to select plant-based menu items from the 28 foot long "performance bar" near the entrance of the dining hall<br>- Reinforcement of healthy dietary messages by the inclusion of greenhouses and gardens at the entrance of the dining hall<br>- Ensuring plant-based options are placed first in line to promote consumption<br>- Placement of plant food first to encourage diners to choose plant-rich menu options	"5 take aways from menu directions" (2016) (1); Residential & Dining Enterprises Stanford

		Dining, Hospitality & Auxiliaries (n.d.) (152); Tanyeri (2017) (2)
University of California, Davis	- Alteration of the menu by listing Beyond Burger and 50/50 burger blends as the first two items on the menu at one retail location	AASHE (n.d.) (187)
University of Wyoming	- Easy to locate vegan options were moved from “the corner” to be made into classic entree station	Victor (2018) (264)
Wake Forest University	- Use of plant-based defaults through the implementation of a “plant first” structure as the default in dining halls and catering services in which plant-based foods are placed at the beginning of the line and animal-based foods are served in smaller portions at the end of the line	Wake Forest University (n.d.) (274)
Washington State University	- Featuring plant-based proteins first in service lines	Humane World for Animals (2025) (37)

References

1. 5 Take Aways from Menu Directions. FoodService Director [Internet]. 2016 Apr 1 [cited 2024 Nov 19];29(4):24. https://research.ebsco.com/linkprocessor/plink?id=219efaff-4c9a-3d50-b4dd-d25611a3ff30
2. Tanyeri, D. Feeding a better college experience: FE&S. Foodservice Equipment & Supplies [Internet]. 2017 July [cited 2024 Nov 19]; 70(7): 26-28,30. https://www.proquest.com/trade-journals/feeding-better-college-experience/docview/1923669217/se-2?accountid=14826
3. Crain, J. College dining goes ultra local with greens. Food Management [Internet]. 2019 May 24 [cited 2024 Nov 19]. https://www.proquest.com/trade-journals/college-dining-goes-ultra-local-with-greens/docview/2229670592/se-2
4. ForwardFood. Forward Food Pledge Participants. (2020). https://www.forwardfood.org/pledge/ [accessed May 8, 2025]
5. Middleton K, Littler KM. Plant Proteins Move to Center-Plate at Colleges and Universities. Thottathil, S.E. & Goger, A.M. (Eds.), Institutions as Conscious Food Consumers: Leveraging Purchasing Power to Drive Systems Change, Elsevier. (2019). 307-324.
6. Association for the Advancement of Sustainability in Higher Education. Appalachian State University OP-8: Sustainable Dining. (n.d.). https://reports.aashe.org/institutions/appalachian-state-university-nc/report/2023-12-05/OP/food-dining/OP-8/ [accessed August 10, 2025]
7. AppState. Sustainability. (n.d.). https://dining.appstate.edu/sustainability [accessed June 26, 2025]
8. Cavanaugh BB. Full-Time Commitment. Foodservice Director [Internet]. 2011 Jan 15. 40.
9. Hempen L. Terra Verde expands plant-forward dining options at App State. 2019 October 1, https://today.appstate.edu/2019/10/01/terra-verde [accessed July 8, 2025]
10. Association for the Advancement of Sustainability in Higher Education. (n.d.). Arizona State University OP-8: Sustainable Dining. https://reports.aashe.org/institutions/arizona-state-university-az/report/2023-02-28/OP/food-dining/OP-8/ [accessed August 6, 2025]
11. US News and World Report. Vegetarian delights \ More colleges are offering courses, choices for meatless meals. The Buffalo News (NY) [Internet]. 2011 Aug 9 [cited 2024 Nov 19]. C5. https://infoweb.newsbank.com/apps/news/document-view?p=WORLDNEWS&docref=news/139075235775D048
12. Johns Hopkins Center for a Livable Future and Meatless Monday. From the Ground Up: Columbia, South Carolina Adopts Meatless Monday. (n.d.). https://meatlessmonday.publichealth.jhu.edu/resources/ground-columbia-south-carolina-adopts-meatless-monday [accessed June 16, 2025]
13. Benedict College, Let's Meat Less, and Forward Food Join Forces to Expand Plant-Based Menu Offerings on the Midlands Campus. (2021). Targeted News Service: Private Colleges and Universities (USA) [Internet]. 2021 August 5 [cited 2024 Nov 19]. https://infoweb.newsbank.com/apps/news/document-view?p=WORLDNEWS&docref=news/1842FB922AEE1108

14. Benedict College and the Forward Food Collaborative join forces to expand plant-based menu offerings throughout campus. The Tiger News: Benedict College (Columbia, SC) [Internet]. 2021 August 17 [cited 2024 Nov 19]. https://infoweb.newsbank.com/apps/news/document-view?p=WORLDNEWS&docref=news/184706DFA5CF0BF0
15. Vt.'s Bennington College said vegan friendly, VT. 2010 October 15.
16. Association for the Advancement of Sustainability in Higher Education. Bentley University OP-8: Sustainable Dining. (n.d.). https://reports.aashe.org/institutions/bentley-university-ma/report/2023-10-19/OP/food-dining/OP-8/ [accessed August 6, 2025]
17. Bentley University Dining Services. Sustainability. (n.d.). https://bentley.acceptance-sodexomyway.net/en-us/explore/sustainability [accessed June 16, 2025]
18. Walsh K, Gowett C. Bentley Dining: Where Sustainability is on the Menu. (2023). https://www.bentley.edu/news/bentley-dining-where-sustainability-menu [accessed May 15, 2025]
19. Berry College Dining Services provides food to students with all dietary restrictions including vegan, vegetarian and gluten free restrictions. In the past six months, Dining Services has intentionally made changes in the food options to accommodate more. Campus Carrier: Berry College (Mount Berry, GA) [Internet]. 2023 February 2 [cited 2024 Nov 19]. https://infoweb.newsbank.com/apps/news/document-view?p=WORLDNEWS&docref=news/18F71521084CE890
20. Association for the Advancement of Sustainability in Higher Education. Binghamton University OP-8: Sustainable Dining. (n.d.). https://reports.aashe.org/institutions/binghamton-university-ny/report/2023-03-02/OP/food-dining/OP-8/ [accessed August 10, 2025]
21. Bernstein J. Binghamton University dining halls implement Meatless Monday. Pipe Dream (Binghamton University) (NY) [Internet]. 2013 December 6 [cited 2024 Nov 19]. https://infoweb.newsbank.com/apps/news/document-view?p=WORLDNEWS&docref=news/14A8AFDDAB214280
22. Van Caeseele C. New dining options aim to diversify food on campus. Pipe Dream (Binghamton University) (NY) [Internet]. 2021 February 11 [cited 2024 Nov 19]. https://infoweb.newsbank.com/apps/news/document-view?p=WORLDNEWS&docref=news/18093F898DA6F308
23. Block K. Major win: 100% of McDonald’s eggs in the US now come from cage-free hens. 2024 February 13. https://www.humaneworld.org/en/blog/Mcdonalds-cage-free-eggs-us [accessed August 11, 2025]
24. Buzalka M. Best Concepts: Seaweed-ish Meatballs help drive sustainability at Boston College. Food Management [Internet]. 2023 July 27 [cited 2024 Nov 19]. https://www.proquest.com/trade-journals/best-concepts-seaweed-ish-meatballs-help-drive/docview/3122682316/se-2
25. Association for the Advancement of Sustainability in Higher Education. Boston University OP-8: Sustainable Dining. (n.d.). https://reports.aashe.org/institutions/boston-university-ma/report/2024-05-31/OP/food-dining/OP-8/ [accessed August 10, 2025]
26. Boston University Dining Services. Plant-Based Dining. (n.d.). https://www.bu.edu/dining/nutrition/vegetarian-and-vegan/ [accessed June 16, 2025]

27. Boston University Dining Services. Sustainability Report – 2023-2024. (2023-2024). https://www.bu.edu/dining/files/2025/01/FY24-BU-Dining-Sustainability-Report.pdf [accessed June 16, 2025]
28. Buzalka M. Boston University streamlines production, adds vegan kosher option. Food Management [Internet]. 2018 May 1 [cited 2024 Nov 19]. https://www.proquest.com/trade-journals/boston-university-streamlines-production-adds/docview/2076880841/se-2
29. Association for the Advancement of Sustainability in Higher Education. Bowdoin College OP-8: Sustainable Dining. (n.d.). https://reports.aashe.org/institutions/bowdoin-college-me/report/2024-05-08/OP/food-dining/OP-8/ [accessed August 10, 2025]
30. Bowdoin. Sustainability Policy. (n.d.). https://www.bowdoin.edu/dining/sustainability/sustainability-policy.html [accessed July 8, 2025]
31. Yale Kamila A. Mystery meat yields to greener meals in Maine college dining halls. Portland Press Herald (ME) [Internet]. 2015 October 21 [cited 2024 Nov 19]. https://infoweb.newsbank.com/apps/news/document-view?p=WORLDNEWS&docref=news/1589DF84AF0640F8
32. Association for the Advancement of Sustainability in Higher Education. Bowling Green State University OP-8: Sustainable Dining. (n.d.). https://reports.aashe.org/institutions/bowling-green-state-university-oh/report/2025-03-07/OP/food-dining/OP-8/ [accessed August 10, 2025]
33. Buzalka M. Five plant-based trends to follow in college dining. Food Management [Internet]. 2023 January 18 [cited 2024 Nov 19]. https://www.proquest.com/trade-journals/five-plant-based-trends-follow-college-dining/docview/3122667956/se-2
34. CoolFood. Our Members. (n.d.). https://coolfood.org/pledge/ [accessed May 8, 2025]
35. Brandeis Hospitality. Sustainability Initiatives. (n.d.). https://www.brandeishospitality.com/sustainability/sustainability-initiatives/ [accessed July 8, 2025]
36. Association for the Advancement of Sustainability in Higher Education. Brigham Young University OP-8: Sustainable Dining. (n.d.). https://reports.aashe.org/institutions/brigham-young-university-ut/report/2024-07-10/OP/food-dining/OP-8/ [accessed August 10, 2025]
37. Humane World for Animals. College and University Protein Sustainability Scorecard. 2025 January. https://www.humaneworld.org/sites/default/files/docs/HWFA_CU-scorecard.pdf [accessed May 8, 2025]
38. Association for the Advancement of Sustainability in Higher Education. Bryn Mawr College OP-8: Sustainable Dining. (n.d.). https://reports.aashe.org/institutions/bryn-mawr-college-pa/report/2023-09-22/OP/food-dining/OP-8/ [accessed August 10, 2025]
39. Local colleges are well-schooled in vegan dining options. Philadelphia Daily News (PA). 2016 November 3, 22.
40. Association for the Advancement of Sustainability in Higher Education. Bucknell University OP-8: Sustainable Dining. (n.d.). https://reports.aashe.org/institutions/bucknell-university-pa/report/2022-11-18/OP/food-dining/OP-8/ [accessed August 10, 2025]

41. Kiernan W. Sailing the Mediterranean. FoodService Director [Internet]. 2010 January 15 [cited 2024 Nov 19]. 23(1):32-34. https://search.ebscohost.com/login.aspx?direct=true&db=bth&AN=47692204&scope=site
42. Association for the Advancement of Sustainability in Higher Education. California Polytechnic State University OP-8: Sustainable Dining. (n.d.). https://reports.aashe.org/institutions/california-polytechnic-state-university-ca/report/2023-07-10/OP/food-dining/OP-8/ [accessed August 10, 2025]
43. Nazar A. Campus Dining adds new vegan protein option. The Poly Post: California State Polytechnic University - Pomona (CA). 2022 March 4.
44. Mitchell L. Meatless Monday' slogan causes flap at Chico State University. Oroville Mercury-Register (CA) [Internet]. 2012 October 11 [cited 2024 Nov 19]. https://infoweb.newsbank.com/apps/news/document-view?p=WORLDNEWS&docref=news/141E4CE3DD1C6098
45. Association for the Advancement of Sustainability in Higher Education. Carnegie Mellon University OP-8: Sustainable Dining. (n.d.). https://reports.aashe.org/institutions/carnegie-mellon-university-pa/report/2024-12-17/OP/food-dining/OP-8/ [accessed August 6, 2025]
46. Galvin C. University offers healthier food options. Tartan, The: Carnegie Mellon University (Pittsburgh, PA). 2016 March 1.
47. Association for the Advancement of Sustainability in Higher Education. Central Washington University OP-8: Sustainable Dining. (n.d.). https://reports.aashe.org/institutions/central-washington-university-wa/report/2022-07-16/OP/food-dining/OP-8/ [accessed August 10, 2025]
48. Buzalka M. Central Washington makes dining upgrades, focuses on sustainability. Food Management [Internet]. 2019 October 31 [cited 2024 Nov 19]. https://www.proquest.com/trade-journals/central-washington-makes-dining-upgrades-focuses/docview/2312503650/se-2
49. Chisholm K. Local colleges offer more plant-based meal options. Worcester Telegram & Gazette: Web Edition Articles (MA) [Internet]. 2019 October 19 [cited 2024 Nov 19]. https://infoweb.newsbank.com/apps/news/document-view?p=WORLDNEWS&docref=news/176B54AB5DAAC5B8
50. Buzalka M. 5 things: Compass North America revenues drop nearly a third in first half of fiscal 2021. Food Management [Internet]. 2021 May 14 [cited 2024 Nov 19]. https://www.proquest.com/trade-journals/5-things-compass-north-america-revenues-drop/docview/2526852661/se-2
51. Chartwells Higher Education Launches 100% Plant-Based Dining Hall Option in Colleges Nationwide - First-ever meatless residential dining hall puts sustainable, plant-based options at the center of the plate. PR Newswire (USA) [Internet]. 2021 May 11 [cited 2024 Nov 19]. https://infoweb.newsbank.com/apps/news/document-view?p=WORLDNEWS&docref=news/1826B95AD4D44A68
52. Colgate University. Sustainable Food and Dining. (n.d.). https://www.colgate.edu/about/sustainability/sustainable-food-dining [accessed on June 17, 2025]

53. Colgate University Dining Services. Sustainability. (n.d.). https://dineoncampus.com/colgate/sustainability [accessed on June 17, 2025]
54. Association for the Advancement of Sustainability in Higher Education. College of Charleston OP-8: Sustainable Dining. (n.d.). https://reports.aashe.org/institutions/college-of-charleston-sc/report/2023-03-03/OP/food-dining/OP-8/ [accessed August 11, 2025]
55. Gruber RE. Kosher Dining Hall Comes to College of Charleston. The Forward: Web Edition Articles (New York, NY), 2015 May 17.
56. Association for the Advancement of Sustainability in Higher Education. Colorado Mountain College OP-8: Sustainable Dining. (n.d.). https://reports.aashe.org/institutions/colorado-mountain-college-co/report/2024-08-20/OP/food-dining/OP-8/ [accessed August 11, 2025]
57. Association for the Advancement of Sustainability in Higher Education. Colorado State University OP-8: Sustainable Dining. (n.d.). https://reports.aashe.org/institutions/colorado-state-university-co/report/2022-11-07/OP/food-dining/OP-8/ [accessed August 8, 2025]
58. Langara College Becomes First Campus in Western Canada to Join Global Meatless Monday Initiative. (2015). Canada NewsWire [Internet]. 2015 March 26 [cited 2024 Nov 19]. https://www.proquest.com/wire-feeds/langara-college-becomes-first-campus-western/docview/1666788240/se-2?accountid=14826
59. Columbia University. Columbia University Commits to New York City Plant-Powered Carbon Challenge. (2023). https://sustainable.columbia.edu/news/columbia-university-commits-new-york-city-plant-powered-carbon-challenge#:~:text=Columbia%20University%20Commits%20to%20New%20York%20City%20Plant-Powered%20Carbon%20Challenge,-December%2005%2C%202023&text=Columbia%20Dining%20is%20the%20first,-based%20emissions%20by%202030.%E2%80%9D&text=Columbia%20Dining%20will%20develop%20and,at%20the%20Columbia%20Climate%20School [accessed on June 17, 2025]
60. Columbia University. Plant-Powered Carbon Challenge. (n.d.). https://dining.columbia.edu/content/plant-powered-carbon-challenge [accessed on June 17, 2025]
61. Schwartz N. Gen Z Takeover: Colleges embrace vegan foods to meet student demand. 2019 Dec 18 [cited 2024 Nov 19]. https://www.highereddive.com/news/gen-z-takeover-colleges-embrace-vegan-foods-to-meet-student-demand/569368/
62. Association for the Advancement of Sustainability in Higher Education. Cornell University OP-8: Sustainable Dining. (n.d.). https://reports.aashe.org/institutions/cornell-university-ny/report/2025-03-06/OP/food-dining/OP-8/ [accessed August 11, 2025]
63. Copman L. At Cornell, sustainable dining tastes delicious. (2024). https://sustainablecampus.cornell.edu/news/cornell-sustainable-dining-tastes-delicious [accessed June 17, 2025]
64. Cornell Dining. Sustainability. (n.d.). https://scl.cornell.edu/residential-life/dining/about-dining/sustainability [accessed June 17, 2025]

65. Cornell University College of Human Ecology and Cornell University the Hotel School Cornell SC Johnson College of Business. (2018). Dining and Service Innovations Across the Health Care Continuum: Insights from 2018 CIHF Roundtable. Healthy Futures. 3(2): 1-17.
66. Wholesome Crave. Wholesome Crave Teams Up With Nestlé Professional and UMass Dining to Spearhead Sustainable Plant-Based Movement. (2023). https://wholesomecrave.com/wholesome-crave-teams-up-with-nestle-professional-and-umass-dining-to-spearhead-sustainable-plant-based-movement/ [accessed June 5, 2025]
67. McBride S. College students expect lots of healthy options when eating on campus. Chicago Sun-Times (IL) [Internet]. 2019 August 8 [cited 2024 Nov 19]. https://infoweb.newsbank.com/apps/news/document-view?p=WORLDNEWS&docref=news/17534A04C9A96F38
68. Association for the Advancement of Sustainability in Higher Education. Drexel University OP-8: Sustainable Dining. (n.d.). https://reports.aashe.org/institutions/drexel-university-pa/report/2023-03-03/OP/food-dining/OP-8/ [accessed August 11, 2025]
69. Drexel University. Campus Dining. (n.d.). https://drexel.edu/studentlife/campus-living/dining [accessed on June 17, 2025]
70. Tanyeri D. College & university innovators: FE&S. Foodservice Equipment & Supplies [Internet]. 2014 July [cited 2024 Nov 19]. 67(7): 20-34,36. https://www.proquest.com/trade-journals/college-amp-university-innovators/docview/1652955463/se-2?accountid=14826
71. Taylor M. Drexel's dining goes green. Food Management [Internet]. 2019 January 3 [cited 2024 Nov 19]. https://www.proquest.com/trade-journals/drexel-s-dining-goes-green/docview/2162703865/se-2?accountid=14826
72. Palmisano R. On-campus vegetarian options under scrutiny amid dining changes. The Chronicle (Duke University) (Durham, NC) [Internet]. 2015 September 23 [cited 2024 Nov 19]. https://infoweb.newsbank.com/apps/news/document-view?p=WORLDNEWS&docref=news/15850092C7AB3608
73. The Menus of Change University Research Collaborative. The Almost Perfect Plant-Forward University. (n.d.). https://static1.squarespace.com/static/5e73dab948933135a78e16ae/t/66c4c87207b0ae30f6777e10/1724172417024/MCURC_AlmostPerfectPlantForwardUniversity.pdf
74. Association for the Advancement of Sustainability in Higher Education. Endicott College OP-8: Sustainable Dining. (n.d.). https://reports.aashe.org/institutions/endicott-college-ma/report/2023-08-21/OP/food-dining/OP-8/ [accessed August 11, 2025]
75. Florida Agricultural and Mechanical University Announces Commitment to Promote Healthier Options On Campus, Puts University at Forefront of Wellness Cultural Shift. US Fed News (USA) [Internet]. 2018 June 12 [cited 2024 Nov 19]. https://infoweb.newsbank.com/apps/news/document-view?p=WORLDNEWS&docref=news/16C85804CC9659E0
76. Association for the Advancement of Sustainability in Higher Education. Florida Institute of Technology OP-8: Sustainable Dining. (n.d.). https://reports.aashe.org/institutions/florida-institute-of-technology-fl/report/2024-02-12/OP/food-dining/OP-8/ [accessed August 11, 2025]
77. New vegan food menus arise on campus. Crimson: Florida Technical College (Orlando, FL). 2017 January 20.
78. Florida State University. Dining. (n.d.). https://sustainablecampus.fsu.edu/campus-initiatives/food [accessed June 17, 2025]

79. Franklin & Marshall College. Sustainability. (n.d.). https://fandmdining.sodexomyway.com/en-us/explore/sustainability [accessed on June 18, 2025]
80. Association for the Advancement of Sustainability in Higher Education. Georgia Institute of Technology OP-8: Sustainable Dining. (n.d.). https://reports.aashe.org/institutions/georgia-institute-of-technology-ga/report/2024-02-29/OP/food-dining/OP-8/ [accessed August 8, 2025]
81. Georgia Tech. Sustainability in Dining. (n.d.). https://dining.gatech.edu/sustainability [accessed June 18, 2025]
82. Zusel Y. Slutty Vegan to open a location on a college campus next month. Atlanta Journal-Constitution, The: Web Edition Articles (GA). 2023 February 2.
83. Kopp E, Meade G. Head to Head: McEwen's Meatless Monday. 2019 November 21. https://spec.hamilton.edu/head-to-head-mcewens-meatless-monday-ee62b17a293f [accessed July 3, 2025]
84. Hamilton College: JHS Program Provides Access and Opportunity. News Bites - Private Companies [Internet]. 2022 February 23 [cited 2024 Nov 19]. https://www.proquest.com/wire-feeds/hamilton-college-jhs-program-provides-access/docview/2631622854/se-2
85. Buzalka M. Harvard Dining meets today's challenges with menu, sustainability and diversity initiatives. Food Management [Internet]. 2023 March 1 [cited 2024 Nov 19]. https://www.proquest.com/trade-journals/harvard-dining-meets-today-s-challenges-with-menu/docview/3122667742/se-2
86. Harvard Office for Sustainability. Healthful and Sustainable Food. (n.d). https://sustainable.harvard.edu/our-plan/how-we-operate/food/ [accessed on June 18, 2025]
87. Harvard University. Sustainability. (n.d.). https://www.dining.harvard.edu/about-huds/sustainability [accessed June 18, 2025]
88. The Menus of Change University Research Collaborative. 2020 MCURC Collective Impact Early Learnings Report. (n.d.). https://static1.squarespace.com/static/5e73dab948933135a78e16ae/t/66c4c838bc70813913e36bb2/1724172357387/2020+MCURCCollective+Impact+Early+Learnings+Report.pdf
89. Association for the Advancement of Sustainability in Higher Education. Ithaca College OP-8: Sustainable Dining. (n.d.). https://reports.aashe.org/institutions/ithaca-college-ny/report/2022-10-25/OP/food-dining/OP-8/ [accessed August 11, 2025]
90. Ithaca College Dining Services Recognized for Outstanding Plant-based Menu Options. Targeted News Service (USA) [Internet]. 2018 February 9 [cited 2024 Nov 19]. https://infoweb.newsbank.com/apps/news/document-view?p=WORLDNEWS&docref=news/169F9D24DCC96668
91. Johns Hopkins Center for a Livable Future and Meatless Monday. Humane Society Holds Plant-Based Culinary Training at John Jay College. (n.d.). https://meatlessmonday.publichealth.jhu.edu/resources/humane-society-holds-plant-based-culinary-training-john-jay-college [accessed June 18, 2025]
92. Johns Hopkins University. Meatless Monday. (n.d.). https://studentaffairs.jhu.edu/dining/events/meatless-monday/ [accessed July 8, 2025]

93. Sentenac H. Johnson & Wales university leading the way with plant-based cuisine. Miami New Times (FL) [Internet]. 2015 April 13 [cited 2024 Nov 19]. https://infoweb.newsbank.com/apps/news/document-view?p=WORLDNEWS&docref=news/155737395548CAA0
94. Association for the Advancement of Sustainability in Higher Education. Kent State University OP-8: Sustainable Dining. (n.d.). https://reports.aashe.org/institutions/kent-state-university-oh/report/2025-03-07/OP/food-dining/OP-8/ [accessed August 11, 2025]
95. Kent State University. Food. (n.d.). https://www.kent.edu/sustainability/food [accessed June 18, 2025]
96. Humane Society: Kent State University Doubles Its Plant-Based Pledge Ahead of National Nutrition Month. Targeted News Service (USA) [Internet]. 2024 February 24 [cited 2024 Nov 19]. https://infoweb.newsbank.com/apps/news/document-view?p=WORLDNEWS&docref=news/19767BC7610268B0
97. Nestlé Professional, Wholesome Crave and UMass Dining launch Purpose-Driven Plant-Based Incubator™ to accelerate shift in college and university menus. PR Newswire (USA) [Internet]. 2023 February 6 [cited 2024 Nov 19]. https://infoweb.newsbank.com/apps/news/document-view?p=WORLDNEWS&docref=news/18F8426614B5BE18
98. Association for the Advancement of Sustainability in Higher Education. Lehigh University OP-8: Sustainable Dining. (n.d.). https://reports.aashe.org/institutions/lehigh-university-pa/report/2023-03-20/OP/food-dining/OP-8/ [accessed August 11, 2025]
99. Lehigh University "Sustainable and Healthful Food Purchasing Policy." (2021).
100. Lehigh University Dining. Lehigh Dining displays sustainable prowess. (2024). https://lehigh.sodexomyway.com/en-us/articles/lehigh-dining [accessed June 18, 2025]
101. Buzalka, M. Health and wellness focus driving plant-based trend. Food Management [Internet]. 2023 May 22 [cited 2024 Nov 19]. https://www.proquest.com/trade-journals/health-wellness-focus-driving-plant-based-trend/docview/3122676953/se-2
102. Estrada, R. Liberty Dining launches plant-forward, gluten-free food truck. Foodservice Director [Internet]. 2022 Sept 6 [cited 2025 June 18]. https://www.foodservicedirector.com/menu-trends/liberty-dining-launches-plant-forward-gluten-free-food-truck
103. Gingerella B. Tips from the best vegan programs. FoodService Director [Internet]. 2019 Feb 22 [cited 2024 Nov 19]. 32(2): 24. https://www.proquest.com/trade-journals/tips-best-vegan-programs/docview/2198412716/se-2?accountid=14826
104. Michigan State University Division of Residential and Hospitality Services. Sustainable Food Procurement Guide. (2021). https://sustainability.msu.edu/get-involved/campus-tips-programs/Sustainable_Food_Procurement_Guide_2021.pdf
105. Cobe, P. Favoring function over form. Restaurant Business [Internet]. 2019 Jan [cited 2024 Nov 19]. 118(1): 38. https://www.proquest.com/trade-journals/favoring-function-over-form/docview/2176250295/se-2?accountid=14826
106. Montclair State University Dining Services. Sustainability. (n.d.). https://www.montclair.edu/dining-services/sustainability/ [accessed June 18, 2025]
107. Association for the Advancement of Sustainability in Higher Education. New York University OP-8: Sustainable Dining. (n.d.). https://reports.aashe.org/institutions/new-york-university-ny/report/2025-02-14/OP/food-dining/OP-8/ [accessed August 8, 2025]

108. New York University. NYU Food Commitments. (n.d.). https://www.nyu.edu/life/sustainability/operations/food0.html [accessed June 19, 2025]
109. New York University. NYU Pledges to Cut Food-Related Greenhouse Gas Emissions 25% by 2030. (2020). https://www.nyu.edu/about/news-publications/news/2020/october/Food_Pledge_2020.html [accessed June 19, 2025]
110. Association for the Advancement of Sustainability in Higher Education. North Carolina State University OP-8: Sustainable Dining. (n.d.). https://reports.aashe.org/institutions/north-carolina-state-university-nc/report/2022-08-09/OP/food-dining/OP-8/ [accessed August 8, 2025]
111. Cobe P. Plant Power. FoodService Director [Internet]. 2017 Oct [cited 2024 Nov 19]. 30(10): 46-56. https://www.proquest.com/trade-journals/plant-power/docview/1952357763/se-2
112. Association for the Advancement of Sustainability in Higher Education. Northeastern University OP-8: Sustainable Dining. (n.d.). https://reports.aashe.org/institutions/northeastern-university-ma/report/2023-02-28/OP/food-dining/OP-8/ [accessed August 8, 2025]
113. Fitzpatrick T. Pump Up the Veggie Volume. Food Management [Internet]. 2013 Feb [cited 2024 Nov 19]. 48(2): 30-34. https://www.proquest.com/trade-journals/pump-up-veggie-volume/docview/1284264397/se-2?accountid=14826
114. Association for the Advancement of Sustainability in Higher Education. Northern Arizona University OP-8: Sustainable Dining. (n.d.). https://reports.aashe.org/institutions/northern-arizona-university-az/report/2023-11-06/OP/food-dining/OP-8/ [accessed August 8, 2025]
115. Association for the Advancement of Sustainability in Higher Education. Northern Michigan University OP-8: Sustainable Dining. (n.d.). https://reports.aashe.org/institutions/northern-michigan-university-mi/report/2024-06-20/OP/food-dining/OP-8/ [accessed August 11, 2025]
116. Fitzpatrick T. No Meat Required. Food Management [Internet]. 2012 Jan [cited 2024 Nov 19]. 47(1): 26-28. https://www.proquest.com/trade-journals/no-meat-required/docview/913374792/se-2?accountid=14826
117. Northwestern Dining. One Day a Week, Cut out Meat: NUCuisine & the Meatless Monday Initiative. 2013 May 20. https://northwesterndining.wordpress.com/tag/meatless-monday/ [accessed June 5, 2025]
118. Sodexo Earns 'Most Vegan-Friendly College in America' at Northwestern University - Northwestern University is named top pick by PETA2 for the second time in the category of small colleges. 2010 Dec 15 [cited 2024 Nov 19]. https://infoweb.newsbank.com/apps/news/document-view?p=WORLDNEWS&docref=news/13423B65970981B8
119. Northwestern tops list of most vegan-friendly colleges. SouthtownStar: Web Edition Articles (Chicago, IL). 2011 November 28.
120. Warmouth M. Steve Mangan: Sharpening the Culinary Focus. Foodservice Director. 2014. 27(3): 32-33.
121. The Menus of Change University Research Collaborative and Stanford Food Institute Residential & Dining Enterprises. MCURC Collective Impact Initiative Progress Report 2024. (2024). https://www.ciaprochef.com/wp-content/uploads/2024/10/MCURC-Collective-Impact-Initiative-Progress-Report-2024.pdf

122. Allen S. Not your dad's cafeteria food colleges rethink dining menus to offer vegan, other options. The Oklahoman (Oklahoma City, OK) [Internet]. 2013 July 18 [cited 2024 Nov 19]. 1A. https://infoweb.newsbank.com/apps/news/document-view?p=WORLDNEWS&docref=news/147A18190E5CF4C8
123. Oregon State University. Sustainability. (n.d.). https://uhds.oregonstate.edu/dining/sustainability#:~:text=%22Planting%20It%20Forward%22,forward%20campaign%2C%20read%20our%20story [accessed June 19, 2025]
124. Buzalka M. Sustainability remains a priority despite the pandemic. Food Management [Internet]. 2021 Nov 9 [cited 2024 Nov 19]. https://www.proquest.com/trade-journals/sustainability-remains-priority-despite-pandemic/docview/2595143323/se-2
125. Providence College. Plant-Based Options at Raymond Dining Hall. (2021). https://dining.providence.edu/plant-based-options-at-raymond-dining-hall/ [accessed June 19, 2025]
126. Buzalka M. Bowl concept big hit at Quinnipiac University. Food Management [Internet]. 2019 July 19 [cited 2024 Nov 19]. https://www.proquest.com/trade-journals/bowl-concept-big-hit-at-quinnipiac-university/docview/2265806763/se-2
127. Vaughn D. We tried the vegan brisket at Rice University. Texas Monthly (TX). 2018 October 10.
128. Abdur-Rahman S. Rider University ditches Aramark food service, gains expanded vegan menu from new provider. The Trentonian, (Trenton, NJ) [Internet]. 2018 June 9 [cited 2024 Nov 19]. https://infoweb.newsbank.com/apps/news/document-view?p=WORLDNEWS&docref=news/16D68C12061C77E8
129. Rider University Dining Services. Sustainability. (n.d.). https://dineoncampus.com/riderdining/sustainability [accessed June 20, 2025]
130. Humane World for Animals. Rochester Institute of Technology commits to 50% plant-based entrees by 2025 and hosts plant-based culinary training. 2023 May 30. https://www.humaneworld.org/en/news/rochester-institute-technology-commits-50-plant-based-entrees-2025-and-hosts-plant-based [accessed August 11, 2025]
131. Association for the Advancement of Sustainability in Higher Education. Rutgers, the State University of New Jersey, New Brunswick Campus OP-7: Food and Beverage Purchasing. (n.d.). https://reports.aashe.org/institutions/rutgers-the-state-university-of-new-jersey-new-brunswick-campus-nj/report/2023-02-27/OP/food-dining/OP-7/ [accessed August 11, 2025]
132. Dwyer S. TOP 100. Foodservice Director [Internet]. 2020 [cited 2024 Nov 19]. 33(3): 28-30,32,34-36,38,40-44. https://www.proquest.com/trade-journals/top-100/docview/2380034334/se-2
133. Rutgers Dining Services. Sustainable Menus. (n.d.). https://food.rutgers.edu/sites/default/files/rutgers-dining-services-sustainability-one-pager.png [accessed June 20, 2025]
134. Taylor M. Out with the beef, in with the beets. Food Management [Internet]. 2017 May 3 [cited 2024 Nov 19]. https://www.proquest.com/trade-journals/out-with-beef-beets/docview/1894446430/se-2
135. Salisbury university dining services earns on a Peta2 vegan report card. US Fed News (USA) [Internet]. 2016 August 4 [cited 2024 Nov 19]. https://infoweb.newsbank.com/apps/news/document-view?p=WORLDNEWS&docref=news/15E91017683AD018

136. Salisbury university dining services earns on a Peta2 vegan report card. US Fed News (USA) [Internet]. 2017 November 13 [cited 2024 Nov 19]. https://infoweb.newsbank.com/apps/news/document-view?p=WORLDNEWS&docref=news/1682A92EB0B76B98
137. Association for the Advancement of Sustainability in Higher Education. San Diego State University OP-8: Sustainable Dining. (n.d.). https://reports.aashe.org/institutions/san-diego-state-university-ca/report/2023-10-23/OP/food-dining/OP-8/ [accessed August 8, 2025]
138. Fitzpatrick T. Roasted Chiles and Fresh Corn Salsa. Food Management [Internet]. 2012 Oct [cited 2024 Nov 19]. 47(10): 44. https://www.proquest.com/trade-journals/roasted-chiles-fresh-corn-salsa/docview/1111739147/se-2?accountid=14826
139. Parseghian P. Fruit Salads 2.0. FoodService Director [Internet]. 2012 Aug 15 [cited 2024 Nov 19]. 25(8): 66. https://search.ebscohost.com/login.aspx?direct=true&db=bth&AN=78557410&scope=site
140. San Diego Union-Tribune. Going meatless on Mondays. 2016 Sept 5. https://www.sandiegouniontribune.com/2014/01/21/going-meatless-on-mondays/ [accessed June 5, 2025]
141. Cobe P. Menus move into the future. FoodService Director [Internet]. 2020 Sept/Oct [cited 2024 Nov 19]. (33)6: 22-23,26. https://www.proquest.com/trade-journals/menus-move-into-future/docview/2448446732/se-2?accountid=14826
142. Association for the Advancement of Sustainability in Higher Education. Seattle University OP-8: Sustainable Dining. (n.d.). https://reports.aashe.org/institutions/seattle-university-wa/report/2024-03-01/OP/food-dining/OP-8/ [accessed August 11, 2025]
143. Redhawk Dining. Sustainability. (n.d). https://dineoncampus.com/seattleu/sustainability [accessed June 19, 2025]
144. Cobe P. Grate expectations. FoodService Director [Internet]. 2018 October [cited 2024 Nov 19]. 31(10): 32-33,36,38,40,42,44. https://www.proquest.com/trade-journals/grate-expectations/docview/2154504970/se-2?accountid=14826
145. Johns Hopkins Center for a Livable Future and Meatless Monday. Innovating Campus Dining at Skidmore College. (n.d.). https://meatlessmonday.publichealth.jhu.edu/resources/innovating-campus-dining-skidmore-college [accessed June 20, 2025]
146. Skidmore College. Skidmore Dining Services. (n.d.). https://www.skidmore.edu/diningservice/sustainability/index.php [accessed June 20, 2025]
147. Skidmore College. Sustainability. (n.d.). https://www.skidmore.edu/sustainability/initiatives/food.php [accessed June 20, 2025]
148. Smith College. Food & Dining. (n.d.). https://www.smith.edu/academics/applied-learning-research/sustainable-smith/action-impact/food-dining [accessed June 20, 2025]
149. Campus Dining: Experience or New Dining Food Options. The Southern Digest: Southern University and A&M College (Baton Rouge, LA) [Internet]. 2021 Nov 2 [cited 2024 Nov 19]. https://infoweb.newsbank.com/apps/news/document-view?p=WORLDNEWS&docref=news/1860679D160A1ED0
150. Buzalka M. St. John Fisher College Dining keeps up the award-winning work. Food Management [Internet]. 2021 May 13 [cited 2024 Nov 19]. https://www.proquest.com/trade-journals/st-john-fisher-college-dining-keeps-up-award/docview/2526329674/se-2

151. Viewpoint: How campus dining supports Stanford's new neighborhood model. Food Management [Internet]. 2021 Oct 22 [cited 2024 Nov 19]. https://www.proquest.com/trade-journals/viewpoint-how-campus-dining-supports-stanford-s/docview/2586941331/se-2
152. Residential & Dining Enterprises Stanford Dining, Hospitality & Auxiliaries. Food Choice Architecture Playbook. (n.d.). https://rde.stanford.edu/sites/default/files/2025-01/FoodChoice%20ArchitecturePlaybook2025.pdf
153. Residential & Dining Enterprises Stanford Dining's Sustainable Food Program. One Plate One Planet. (n.d.). https://rde.stanford.edu/sites/default/files/Dining/PDF/One%20Plate%20One%20Planet%20Overview%20-%20Document%20%282%29.pdf [accessed June 20, 2025]
154. Stanford Dining, Hospitality & Auxiliaries Residential & Dining Enterprises. Eat Well @ Stanford. (n.d.). https://rde.stanford.edu/dining-hospitality/eat-well-stanford#mcurc [accessed June 20, 2025]
155. Stanford Dining, Hospitality & Auxiliaries Residential & Dining Enterprises. One Plate One Planet. (n.d.). https://rde.stanford.edu/dining-hospitality/one-plate-one-planet [accessed June 20, 2025]
156. Association for the Advancement of Sustainability in Higher Education. Syracuse University OP-8: Sustainable Dining. (n.d.). https://reports.aashe.org/institutions/syracuse-university-ny/report/2025-01-23/OP/food-dining/OP-8/ [accessed August 8, 2025]
157. David George. Foodservice Director. 2010 June 15, 23, 18.
158. King P. Meatless Monday Muddle. FoodService Director [Internet]. 2013 Oct 15 [cited 2024 Nov 19]. (26):10, 52-52,56,58. https://www.proquest.com/trade-journals/meatless-monday-muddle/docview/1449841721/se-2?accountid=14826
159. Syracuse University. Sustainability. (n.d.). https://campusdining.syr.edu/aboutus/sustainability/ [accessed June 20, 2025]
160. Association for the Advancement of Sustainability in Higher Education. The Ohio State University OP-8: Sustainable Dining. (n.d.). https://reports.aashe.org/institutions/the-ohio-state-university-oh/report/2023-06-30/OP/food-dining/OP-8/ [accessed August 8, 2025]
161. Buzalka M. 5 things: School removes child's lunch for meal debt policy. Food Management [Internet]. 2019 Sept 12 [cited 2024 Nov 19]. https://www.proquest.com/trade-journals/5-things-school-removes-child-s-lunch-meal-debt/docview/2289624687/se-2
162. Cagle B. Thyme for a Change: New Vegan Food Truck Opening on Campus. 2019 Sept 9 [cited 2025 June 20]. https://www.thelantern.com/2019/09/thyme-for-a-change-new-vegan-food-truck-opening-on-campus/
163. Lerner, S. Dining Services brings new updates to campus this spring. The Lantern: Ohio State University (Columbus, OH) [Internet]. 2018 January 18 [cited 2024 Nov 19]. https://infoweb.newsbank.com/apps/news/document-view?p=WORLDNEWS&docref=news/169885C3A1AA35B8
164. Parismcgee. Dining services works to bring vegan and vegetarian options to campus. The Lantern: Ohio State University (Columbus, OH) [Internet]. 2017 Sept 27 [cited 2024 Nov 19]. https://infoweb.newsbank.com/apps/news/document-view?p=WORLDNEWS&docref=news/1672F05BC1ADEA98

165. Dinner is different: The newest in campus dining. The Lantern: Ohio State University (Columbus, OH) [Internet]. 2019 August 19 [cited 2024 Nov 19]. https://infoweb.newsbank.com/apps/news/document-view?p=WORLDNEWS&docref=news/175E008F15BE8740
166. Association for the Advancement of Sustainability in Higher Education. Pennsylvania State University OP-8: Sustainable Dining. (n.d.). https://reports.aashe.org/institutions/pennsylvania-state-university-pa/report/2023-12-12/OP/food-dining/OP-8/ [accessed August 8, 2025]
167. Healey P. University of Dayton Makes Over 40 Percent of Entrees Plant-Based in Sustainability Push. (2023). https://www.speciesunite.com/news-stories/university-of-dayton-makes-over-40-percent-of-entrees-plant-based-in-sustainability-push [accessed June 5, 2025]
168. Association for the Advancement of Sustainability in Higher Education. (n.d.). University of Arizona OP-8: Sustainable Dining. (n.d.). https://reports.aashe.org/institutions/university-of-arizona-az/report/2025-01-06/OP/food-dining/OP-8/ [accessed August 11, 2025]
169. The University of Arizona. Nutrition Programs. (n.d.). https://union.arizona.edu/nutrition/programs [accessed June 21, 2025]
170. Meatless Monday. Universities Get Inspired Introducing Meatless Monday. (n.d.). https://www.mondaycampaigns.org/meatless-monday/news/universities-get-inspired-introducing-meatless-monday#:~:text=Preparing%20more%20vegetarian%20and%20vegan,the%20environmental%20cost%20of%20meat.%E2%80%9D [accessed June 23, 2025]
171. Miller V. Meat producers cry foul on University of Iowa Student Government 'Meatless Monday' initiative - Meat still available in dining halls every day - but student group promoting meat-free options. The Gazette (Cedar RapidsIowa City, IA) [Internet]. 2018 Feb 22 [cited 2024 Nov 19]. https://infoweb.newsbank.com/apps/news/document-view?p=WORLDNEWS&docref=news/16A3FEEDA638D210
172. University of Iowa holds first Meatless Monday at dining halls. Daily Iowan, The: University of Iowa (Iowa City, IA) [Internet]. 2018 Feb 6 [cited 2024 Nov 19]. https://infoweb.newsbank.com/apps/news/document-view?p=WORLDNEWS&docref=news/169E687B526B06E0
173. Association for the Advancement of Sustainability in Higher Education. University of Oklahoma OP-8: Sustainable Dining. (n.d.). https://reports.aashe.org/institutions/university-of-oklahoma-ok/report/2025-03-07/OP/food-dining/OP-8/ [accessed August 11, 2025]
174. Association for the Advancement of Sustainability in Higher Education. University of Texas at Austin OP-8: Sustainable Dining. (n.d.). https://reports.aashe.org/institutions/university-of-texas-at-austin-tx/report/2024-11-04/OP/food-dining/OP-8/ [accessed August 11, 2025]
175. Humane World for Animals. The University of Texas reaches major milestone with half of all meals served being plant based. 2024 March 1. https://www.humaneworld.org/en/news/university-texas-reaches-major-milestone-half-all-meals-served-being-plant-based [accessed August 11, 2025]

176. World Resources Institute: PepsiCo, University of Texas at Austin, ISS, Healthcare Systems Pledge to Serve Planet-Saving 'Cool Food'. Targeted News Service (USA) [Internet]. 2022 April 24 [cited 2024 Nov 19]. https://infoweb.newsbank.com/apps/news/document-view?p=WORLDNEWS&docref=news/18995160618CE9B8
177. Association for the Advancement of Sustainability in Higher Education. University of Utah OP-8: Sustainable Dining. (n.d.). https://reports.aashe.org/institutions/university-of-utah-ut/report/2023-09-12/OP/food-dining/OP-8/ [accessed August 8, 2025]
178. Rooted offers new plant-based dining options on campus. Daily Utah Chronicle, The: University of Utah (Salt Lake City, UT) [Internet]. 2018 Nov 10 [cited 2024 Nov 19]. https://infoweb.newsbank.com/apps/news/document-view?p=WORLDNEWS&docref=news/16F9EB9C63EFC0B8
179. Obineme O. New vegetarian, vegan options now on-campus. Towerlight, The: Towson University (MD) [Internet]. 2013 March 1 [cited 2024 Nov 19]. https://infoweb.newsbank.com/apps/news/document-view?p=WORLDNEWS&docref=news/1482E478CD057F00 (accessed November 19, 2024)
180. Towson University in the running for most vegan-friendly college in the U.S. Targeted News Service (USA) [Internet]. 2012 Oct 13 [cited 2024 Nov 19]. https://infoweb.newsbank.com/apps/news/document-view?p=WORLDNEWS&docref=news/141E5280361ECC20
181. Towson University. TU's Food System. (2021). https://storymaps.arcgis.com/stories/1c297f2e17b44aa083d2eea98b47a81f [accessed June 21, 2025]
182. Towson University. Sustainability. (n.d.). https://www.towson.edu/about/sustainability/#:~:text=Cool%20Foods,halls%20and%20at%20catered%20events [accessed June 21, 2025]
183. Association for the Advancement of Sustainability in Higher Education. University at Albany OP-8: Sustainable Dining. (n.d.). https://reports.aashe.org/institutions/university-at-albany-ny/report/2023-02-27/OP/food-dining/OP-8/ [accessed August 8, 2025]
184. Buzalka M. SUNY Albany chefs expand veg cooking skills. Food Management [Internet]. 2018 Oct 15 [cited 2024 Nov 19]. https://www.proquest.com/trade-journals/suny-albany-chefs-expand-veg-cooking-skills/docview/2120118077/se-2
185. UC Berkeley and The Humane Society Collaborate to Make Campus Dining More Plant-Based. One Green Planet (USA). 2023 Dec 14.
186. Sanchez V. Berkeley Dining: Farm to Belly. (2022). https://life.berkeley.edu/cal-dining-farm-to-belly/ [accessed June 5, 2025]
187. Association for the Advancement of Sustainability in Higher Education. University of California, Davis OP-8: Sustainable Dining. (n.d.). https://reports.aashe.org/institutions/university-of-california-davis-ca/report/2023-06-20/OP/food-dining/OP-8/ [accessed August 8, 2025]
188. UC Davis. Food & Dining. (2021). https://sustainability.ucdavis.edu/goals/food [accessed July 8, 2025]

189. Association for the Advancement of Sustainability in Higher Education. University of California, Irvine OP-8: Sustainable Dining. (n.d.). https://reports.aashe.org/institutions/university-of-california-irvine-ca/report/2025-03-07/OP/food-dining/OP-8/ [accessed August 8, 2025]
190. Association for the Advancement of Sustainability in Higher Education. University of California, Los Angeles OP-8: Sustainable Dining. (n.d.). https://reports.aashe.org/institutions/university-of-california-los-angeles-ca/report/2023-09-08/OP/food-dining/OP-8/ [accessed August 8, 2025]
191. Alamdari I. Veggie Grill to sprout on campus, offers exclusively plant-based menu. Daily Bruin: University of California-Los Angeles (CA) [Internet]. 2018 May 13 [cited 2024 Nov 19]. https://infoweb.newsbank.com/apps/news/document-view?p=WORLDNEWS&docref=news/16BE4F53E139FF28
192. UCLA Annual Foodservice Sustainability Policy Report. (2018–2019). https://www.sustain.ucla.edu/wp-content/uploads/2021/07/18-19-Foodservice-Report_UCLADining.pdf
193. Annual UCLA Foodservices Sustainability Report. (2010-2011). https://www.sustain.ucla.edu/wp-content/uploads/2013/05/Annual-UCLA-Foodservices-Report-2011.pdf
194. Cleveland DA, Jay JA. Integrating climate and food policies in higher education: a case study of the University of California. Climate Policy (2020). 21:1, 16–32. doi: 10.1080/14693062.2020.1787939
195. UCLA's New All-Healthy Dining Hall. Food Management [Internet]. 2014 Feb [cited 2024 Nov 19]. 49(2): 8. https://www.proquest.com/trade-journals/uclas-new-all-healthy-dining-hall/docview/1508544561/se-2
196. Smith, K. Vegan Restaurant Chain Veggie Grill Replaces Pizza Joint at UCLA. Live Kindly. (n.d.) [cited 2025 June 5]. https://www.livekindly.com/vegan-restaurant-chain-veggie-grill-replaces-pizza-joint-at-ucla/
197. UCLA, a soon to be vegan campus? Los Angeles Examiner (CA). 2012 July 5.
198. UCLA Dining Services. Bruin Plate. (n.d.). https://bruinplate.hh.ucla.edu/index.php [accessed June 23, 2025]
199. UCLA Sustainability. Residential Dining. https://sustain.ucla.edu/housing/dining-green/ (n.d.). [accessed June 23, 2025]
200. Plascencia G. Sustainable Food Service Practices Annual Report to UCOP. (2013). https://www.ucop.edu/sustainability/_files/foodservice/ucr-foodservice-report-12-13.pdf
201. UC Riverside Dining Services. UCR Dining Services Sustainability. (n.d.). https://dining.ucr.edu/ucr-dining-services-sustainability [accessed June 23, 2025]
202. Association for the Advancement of Sustainability in Higher Education. University of California, San Diego OP-8: Sustainable Dining. (n.d.). https://reports.aashe.org/institutions/university-of-california-san-diego-ca/report/2024-11-05/OP/food-dining/OP-8/ [accessed August 8, 2025]
203. Mitchell A. Plant power fast food just opened at university of California San Diego. VegOut [Internet]. 2021 [cited 2025 June 23]. https://vegoutmag.com/news/plant-power-fast-food-just-opened-at-university-of-california-san-diego/ (accessed June 23, 2025)

204. McCord S. UC Santa Cruz students push campus to adopt meat-free Mondays. Santa Cruz Sentinel (CA) [Internet]. 2010 Sept 21 [cited 2024 Nov 19]. https://infoweb.newsbank.com/apps/news/document-view?p=WORLDNEWS&docref=news/13266CFBB605D198
205. Vegan fast-food chain opens second college campus location - Plant Power Fast Food hits significant expansion milestone with the opening of its newest location on the campus of the University of California at San Diego. PR Newswire (USA) [Internet]. 2021 Aug 16 [cited 2024 Nov 19]. https://infoweb.newsbank.com/apps/news/document-view?p=WORLDNEWS&docref=news/1846976B0B30F998
206. UC San Diego. Rooted in Flavor. (n.d.). https://hdhdining.ucsd.edu/nutrition-services/rooted/index.html [accessed July 3, 2025]
207. Association for the Advancement of Sustainability in Higher Education. University of California, Santa Barbara OP-8: Sustainable Dining. (n.d.). https://reports.aashe.org/institutions/university-of-california-santa-barbara-ca/report/2024-02-29/OP/food-dining/OP-8/ [accessed August 11, 2025]
208. Cobe P. Comfort with a dash of health. FoodService Director [Internet]. 2015 Nov 15 [cited 2024 Nov 19]. 28(11): 14. https://search.ebscohost.com/login.aspx?direct=true&db=bth&AN=110655494&scope=site
209. UCCS Dining and Hospitality to add more plant-based dishes to campus eateries. Scribe, The: University of Colorado - Colorado Springs (CO) [Internet]. 2017 Jan 31 [cited 2024 Nov 19]. https://infoweb.newsbank.com/apps/news/document-view?p=WORLDNEWS&docref=news/162412C6853E8780
210. University of Colorado Colorado Springs. Sustainability. (n.d.). https://diningservices.uccs.edu/about/sustainability [accessed June 23, 2025]
211. Association for the Advancement of Sustainability in Higher Education. University of Connecticut OP-8: Sustainable Dining. (n.d.). https://reports.aashe.org/institutions/university-of-connecticut-ct/report/2024-12-30/OP/food-dining/OP-8/ (accessed August 11, 2025)
212. Dunne S. UConn Dining dish wins best vegan recipe at national colleges contest. Hartford Courant, The: Web Edition Articles [TCA] (CT). 2022 May 18.
213. Estrada R. University of Dayton surpasses its plant-based goal. Foodservice Director [Internet]. 2023 Oct 17 [cited 2025 June 23]. https://www.foodservicedirector.com/sustainability/university-of-dayton-surpasses-its-plant-based-goal
214. Humane Society: University of Dayton and Forward Food Collaborative Announce Joint Effort to Increase Plant-Based Options. Targeted News Service (USA) [Internet]. 2021 July 29 [cited 2024 Nov 19]. https://infoweb.newsbank.com/apps/news/document-view?p=WORLDNEWS&docref=news/1840ACBAD4D98258
215. Humane Society: University of Dayton Leads the Way in Swift Plant-Based Menu Changes. Targeted News Service (USA) [Internet]. 2023 Oct 19 [cited 2024 Nov 19]. https://infoweb.newsbank.com/apps/news/document-view?p=WORLDNEWS&docref=news/194C49E85CCAFCA8
216. University of Dayton. Sustainability Goals. (n.d.). https://udayton.edu/sustainability/goals.php#:~:text=By%202025%2C%20identify%20opportunities%20for,supplier%20traceab

ility%20and%20sourcing%20flexibility).&text=Annually%20increase%20UD's%20spend%20on%20locally%20produced%20food.&text=By%202025%2C%20in%20partnership%20with,spend%20on%20local%20food%20purchases.&text=By%202025%2C%20complete%20spend%20analysis%20of%20locally%20purchased%20foods.&text=By%202026%2C%20with%20campus%20partners,purchasing%20and%20receiving%20local%20products.&text=Within%205%20years%2C%20increase%20UD's,45%25%20from%20a%202022%20baseline.&text=DIN4a-,By%202025%2C%20in%20partnership%20with%20vendors%20and%20distributors%2C%20improve%20tracking,of%20plant-based%20food%20purchases.&text=By%202027%2C%20increase%20the%20number,30%25%20from%20a%202022%20baseline.&text=Annually%20maintain%20Green%20Restaurant%20Association,or%20higher%20or%20similar%20certification.&text=By%202025%2C%20annually%20inform%20all,dining%20facilities%20and%20catering%20menus [accessed June 23, 2025]

217. Dozens of New Vegan Options Spring Up at University of Florida. Targeted News Service (USA) [Internet]. 2014 Oct 1 [cited 2024 Nov 19]. https://infoweb.newsbank.com/apps/news/document-view?p=WORLDNEWS&docref=news/150AF228D50D7B08
218. Peta2 Recognizes Sodexo at the University of Hawaii at Manoa for Vegan Cuisine. 3BL Media (USA) [Internet]. 2018 Dec 12 [cited 2024 Nov 19]. https://infoweb.newsbank.com/apps/news/document-view?p=WORLDNEWS&docref=news/17044386CF5746D0
219. Association for the Advancement of Sustainability in Higher Education. University of Illinois, Urbana-Champaign OP-8: Sustainable Dining. (n.d.). https://reports.aashe.org/institutions/university-of-illinois-urbana-champaign-il/report/2025-04-29/OP/food-dining/OP-8/ [accessed August 8, 2025]
220. Cobe P. Word from the street. FoodService Director [Internet]. 2016 Oct [cited 2024 Nov 19]. 29(10): 52-68. https://www.proquest.com/trade-journals/word-street/docview/1831824087/se-2?accountid=14826
221. Curtis T. University of Maryland joins global food sustainability pledge. The Daily Record (Baltimore, MD). 2019 Aug 26.
222. World Resources Institute: Maryland Becomes the First 'Cool Food' University. Targeted News Service (USA) [Internet]. 2019 Aug 26 [cited 2024 Nov 19]. https://infoweb.newsbank.com/apps/news/document-view?p=WORLDNEWS&docref=news/17594A7F6A592020
223. Buzalka M. UMass Dining does more than serve meals. Food Management [Internet]. 2018 April 20 [cited 2024 Nov 19]. https://www.proquest.com/trade-journals/umass-dining-does-more-than-serve-meals/docview/2028107146/se-2
224. Buzalka M. 5 things: Compass North America sees 23% organic revenue increase in Q1. Food Management [Internet]. 2023 Feb 10 [cited 2024 Nov 19]. https://www.proquest.com/trade-journals/5-things-compass-north-america-sees-23-organic/docview/3122670098/se-2
225. UMass goes all in on health with dining renovation. Foodservice Director. 2013 Nov 15. 26(11): 10.

226. As Demand for Plant-Based Products Soars, Nasoya Expands to Food Service with Key University and Fast Casual Restaurant Accounts with Popular Plantspired™ Line - America's #1 Tofu Brand Anticipates Plant-Based Meal Solutions Line Sales to Double in 2022. PR Newswire (USA) [Internet]. 2021 Nov 9 [cited 2024 Nov 19]. https://infoweb.newsbank.com/apps/news/document-view?p=WORLDNEWS&docref=news/18629CEE83378838
227. Third-party certification improves healthy option consumption at UMass. Foodservice Director [Internet]. 2013 Sept 15 [cited 2024 Nov 19]. 26(9): 12. https://www.proquest.com/trade-journals/third-party-certification-improves-healthy-option/docview/1441093220/se-2
228. Tierney K. Hampshire Dining Commons renovation (2013). (2024). https://www.umass.edu/planning-design-construction/book/export/html/283 [accessed June 11, 2025]
229. Bolivar L. College campus dining undergoes a revolution. The Miami Herald (FL) [Internet]. 2010 March 21 [cited 2024 Nov 19]. https://infoweb.newsbank.com/apps/news/document-view?p=WORLDNEWS&docref=news/12F61C0BC61294A8
230. Vegan-friendly options increasing in campus dining halls. Miami Hurricane, The: University of Miami (Coral Gables, FL) [Internet]. 2018 Sept 25 [cited 2024 Nov 19]. https://infoweb.newsbank.com/apps/news/document-view?p=WORLDNEWS&docref=news/16EA87DB42E84EF8
231. University of Miami Dining. A Healthier U: A Complete Guide to Eating on Campus with Allergies, Intolerances, and Special Dietary Needs. (n.d.). https://dineoncampus.com/files/section_documents/2fec4928-3c01-4545-9b28-a7e117de601d.pdf [accessed June 23, 2025]
232. Buzalka M. 5 things: University of Michigan commits to 55% plant-based by 2025. Food Management [Internet]. 2022 Dec 1 [cited 2024 Nov 19]. https://www.proquest.com/trade-journals/5-things-university-michigan-commits-55-plant/docview/3122666887/se-2
233. Lambrecht NJ, Hoey L, Bryan A, Heller M, Jones AD. Limiting red meat availability in a university food service setting reduces food-related greenhouse gas emissions by one-third. Climatic Change (2023) 176:6. doi: 10.1007/s10584-023-03543-y
234. McCarty T. The University should change the status quo in our food system. The Michigan Daily: University of Michigan (Ann Arbor, MI) [Internet]. 2021 April 14 [cited 2024 Nov 19]. https://infoweb.newsbank.com/apps/news/document-view?p=WORLDNEWS&docref=news/181DE162D890BA10
235. Michigan Dining. Carbon Emissions. (n.d.). https://dining.umich.edu/sustainability/environmental-sustainability/carbon-emissions/ [accessed August 8, 2025]
236. Association for the Advancement of Sustainability in Higher Education. University of Nebraska – Lincoln OP-8: Sustainable Dining. (n.d.). https://reports.aashe.org/institutions/university-of-nebraska-lincoln-ne/report/2022-12-12/OP/food-dining/OP-8/ [accessed August 11, 2025]
237. DeChellis J. University of New Hampshire on track to be healthiest campus by 2020. Food Management [Internet]. 2017 April 18 [cited 2024 Nov 19]. https://www.proquest.com/trade-journals/university-new-hampshire-on-track-be-healthiest/docview/1889311760/se-2

238. Meyer M. Vegan students navigate dining halls and stick to a strict diet on campus. Daily Tar Heel: University of North Carolina at Chapel Hill (NC) [Internet]. 2019 Jan 9 [cited 2024 Nov 19]. https://infoweb.newsbank.com/apps/news/document-view?p=WORLDNEWS&docref=news/170DC78221E5DB40
239. Buzalka M. Residential Dining at University of North Texas augments program with new Eagle Landing venue. Food Management [Internet]. 2021 Aug 3 [cited 2024 Nov 19]. https://www.proquest.com/trade-journals/residential-dining-at-university-north-texas/docview/2557510475/se-2
240. University of North Texas Dining Services. Mean Greens Café. (n.d.). https://dining.unt.edu/mean-greens-cafe/ [accessed June 24, 2025]
241. Local colleges win 'Most Vegan-Friendly' award. Washington Examiner (DC). 2011 Dec 9.
242. White L. Rooting In: FE&S. Foodservice Equipment & Supplies [Internet]. 2019 [cited 2024 Nov 19]. (72)7: 82-84,86. https://www.proquest.com/trade-journals/rooting/docview/2348326386/se-2
243. Association for the Advancement of Sustainability in Higher Education. University of Oregon OP-8: Sustainable Dining. (n.d.). https://reports.aashe.org/institutions/university-of-oregon-or/report/2023-08-01/OP/food-dining/OP-8/ [accessed August 11, 2025]
244. Halnon E. UO's campus dining serves up good food, and sustainability. (2024). https://news.uoregon.edu/dining-services-sustainability [accessed 24 June 2025]
245. Association for the Advancement of Sustainability in Higher Education. University of Pennsylvania OP-8: Sustainable Dining. (n.d.). https://reports.aashe.org/institutions/university-of-pennsylvania-pa/report/2025-02-18/OP/food-dining/OP-8/ [accessed August 8, 2025]
246. Association for the Advancement of Sustainability in Higher Education. University of Pittsburgh OP-7: Food and Beverage Purchasing. (n.d.). https://reports.aashe.org/institutions/university-of-pittsburgh-pa/report/2024-02-13/OP/food-dining/OP-7/ [accessed August 11, 2025]
247. Association for the Advancement of Sustainability in Higher Education. University of Pittsburgh OP-8: Sustainable Dining. (n.d.). https://reports.aashe.org/institutions/university-of-pittsburgh-pa/report/2024-02-13/OP/food-dining/OP-8/ [accessed August 8, 2025]
248. Pitt Sustainability. CoolFood Pledge. (n.d.). https://www.sustainable.pitt.edu/cool-food-pledge/ [accessed June 24, 2025]
249. Schackner B. Fear not: college life after covid-19 will include mac and cheese, lattes local schools confident their food venues will be ready for fall reopening. Pittsburgh Post-Gazette (PA) [Internet]. 2021 July 6 [cited 2024 Nov 19]. B-5. https://infoweb.newsbank.com/apps/news/document-view?p=WORLDNEWS&docref=news/18390211CC789C98
250. Barlett PF. Campus sustainable food projects: Critique and engagement. Am Anthropol (2011) 113(1): 101–115. doi: 10.1111/j.1548-1433.2010.01309.x
251. Dowd WM. Cornell tops Upstate entries on list of best college food. Albany Examiner (NY). 2015 Aug 26.

252. Association for the Advancement of Sustainability in Higher Education. University of San Diego OP-8: Sustainable Dining. (n.d.). https://reports.aashe.org/institutions/university-of-san-diego-ca/report/2024-10-22/OP/food-dining/OP-8/ [accessed August 8, 2025]
253. University of San Diego. Sustainability. (n.d.). https://www.sandiego.edu/dining/resources/sustainabilty.php [accessed June 24, 2025]
254. University of South Florida in the running for most vegan friendly college in the U.S. Targeted News Service (USA) [Internet]. 2012 Oct 13 [cited 2024 Nov 19]. https://infoweb.newsbank.com/apps/news/document-view?p=WORLDNEWS&docref=news/141E5280488B6C78
255. Association for the Advancement of Sustainability in Higher Education. University of Southern California OP-8: Sustainable Dining. (n.d.). https://reports.aashe.org/institutions/university-of-southern-california-ca/report/2023-12-20/OP/food-dining/OP-8/ [accessed August 8, 2025]
256. Baxley S. Dining on campus: Plant-based food offerings at UVM. Food Systems Master's Project Reports. (2021). 18. https://scholarworks.uvm.edu/fsmpr/18
257. University of Vermont. Sustainability. (n.d.). https://uvmdining.sodexomyway.com/en-us/explore/sustainability [accessed June 24, 2025]
258. Association for the Advancement of Sustainability in Higher Education. University of Virginia OP-8: Sustainable Dining. (n.d.). https://reports.aashe.org/institutions/university-of-virginia-va/report/2024-02-08/OP/food-dining/OP-8/ [accessed August 8, 2025]
259. LETTER: The University strives for more sustainable, plant-based dining options. The Cavalier Daily: University of Virginia (Charlottesville, VA) [Internet]. 2020 May 17 [cited 2024 Nov 19]. https://infoweb.newsbank.com/apps/news/document-view?p=WORLDNEWS&docref=news/17B04C6253F67940
260. UVA Dine. What We're Doing on Grounds. (n.d.). https://virginia.campusdish.com/en/sustainability/whatarewedoing/ [accessed June 25, 2025]
261. UVA Sustainable Food Collaborative, A Task Force of the UVA Environmental Stewardship Subcommittee on Sustainability. (2021-2030). University of Virginia Sustainable Food Action Plan.
262. Fitzpatrick T. Food as Medicine. Food Management [Internet]. 2018 Nov 7 [cited 2024 Nov 19]. https://www.proquest.com/trade-journals/food-as-medicine/docview/2130427151/se-2?accountid=14826
263. Meatless Mondays Modification. FoodService Director [Internet]. 2014 May 15 [cited 2024 Nov 19]. (27): 5. https://search.ebscohost.com/login.aspx?direct=true&db=bth&AN=95886411&scope=site
264. Victor J. Plant-based plates. Laramie Boomerang (WY) [Internet]. 2018 April 26 [cited 2024 Nov 19]. A1. https://infoweb.newsbank.com/apps/news/document-view?p=WORLDNEWS&docref=news/16B87C4F91521CD0\

265. Association for the Advancement of Sustainability in Higher Education. Vanderbilt University OP-8: Sustainable Dining. (n.d.). https://reports.aashe.org/institutions/vanderbilt-university-tn/report/2024-02-29/OP/food-dining/OP-8/ [accessed August 8, 2025]
266. Vanderbilt University. Campus Dining. (n.d.). https://campusdining.vanderbilt.edu/menus-of-change-research-collaborative-at-vu/ [accessed June 25, 2025]
267. Lehmkuhl V. V for Villanova - and vegan food in campus dining halls. Philadelphia Daily News (PA) [Internet]. 2016 Nov 3 [cited 2024 Nov 19]. 21. https://infoweb.newsbank.com/apps/news/document-view?p=WORLDNEWS&docref=news/1606C1061481E0C0
268. Stoessel E. FM Live! 2014. Food Management [Internet]. 2014 [cited 2024 Nov 19]. 49(9); 24. https://www.proquest.com/trade-journals/fm-live-2014/docview/1560995369/se-2
269. Villanova University Sustainability. Dining. (n.d.). https://www1.villanova.edu/villanova/sustainability/CampusSustainabilityBuildingsGroundsStormwaterDiningRecycling/DiningServicesSustainability.html [accessed June 25, 2025]
270. Association for the Advancement of Sustainability in Higher Education. Virginia Tech OP-8: Sustainable Dining. (n.d.). https://reports.aashe.org/institutions/virginia-tech-va/report/2024-05-16/OP/food-dining/OP-8/ [accessed August 11, 2025]
271. Buzalka M. Virginia Tech builds on culinary, sustainability successes. Food Management [Internet]. 2018 May 1 [cited 2024 Nov 19]. https://www.proquest.com/trade-journals/virginia-tech-builds-on-culinary-sustainability/docview/2076878410/se-2
272. Growing the edge of plant-forward campus dining. US Fed News (USA) [Internet]. 2022 Jan 12 [cited 2024 Nov 19]. https://infoweb.newsbank.com/apps/news/document-view?p=WORLDNEWS&docref=news/1877F94AB263BFF0
273. McMullen A. Sustainable Eating Options Improving on Campus. Old Gold and Black: Wake Forest University (Winston-Salem, NC) [Internet]. 2022 Jan 28 [cited 2024 Nov 19]. https://infoweb.newsbank.com/apps/news/document-view?p=WORLDNEWS&docref=news/187CED4B28B1DCA8
274. Wake Forest University. Dining & Food Systems. (n.d.). https://sustainability.wfu.edu/operations/dining-food-systems/ [accessed June 25, 2025]
275. Wake Forest University. Plant-Forward Menus. (n.d.). https://dining.wfu.edu/sustainability/plant-forward-menus/ [accessed June 25, 2025]
276. Humane Society: Western Oregon University Pledges to Serve 50% Plant-Based Meals on Campus by 2027. Targeted News Service (USA) [Internet]. 2024 June 8 [cited 2024 Nov 19]. https://infoweb.newsbank.com/apps/news/document-view?p=WORLDNEWS&docref=news/19994081CA722220
277. Association for the Advancement of Sustainability in Higher Education. Williams College OP-8: Sustainable Dining. (n.d.). https://reports.aashe.org/institutions/williams-college-ma/report/2025-04-10/OP/food-dining/OP-8/ [accessed August 11, 2025]

278. Evans M. Dining Stir Fries up more Plant-Rich Options with Help from the CIA. (2024). https://sustainability.williams.edu/news-events/dining-stir-fries-up-more-plant-rich-options-and-seeks-help-from-the-cia/ [accessed 7 July 2025]
279. Williams College. Coolfood at Williams: Engaging Students in Sustainable Choices. (2024). https://sustainability.williams.edu/news-events/coolfood-at-williams-engaging-students-in-sustainable-choices/ [accessed June 25, 2025]
280. Williams College. Sustainable Food. (n.d.). https://sustainability.williams.edu/sustainability-strategic-planning/food/ [accessed May 19, 2025]
281. Association for the Advancement of Sustainability in Higher Education. Yale University OP-8: Sustainable Dining. (n.d.). https://reports.aashe.org/institutions/yale-university-ct/report/2022-06-29/OP/food-dining/OP-8/ [accessed August 8, 2025]
282. Nestlé Professional & Sweet Earth® Partner with University of Massachusetts, Yale, Notre Dame & Other U.S. Colleges to expand innovative plant-based dining options - Universities are meeting increased student demand for plant-based food with Bac'n Cheezeburger and other culinary offerings for World Vegan Month. PR Newswire (USA) [Internet]. 2020 Nov 10 [cited 2024 Nov 19]. https://infoweb.newsbank.com/apps/news/document-view?p=WORLDNEWS&docref=news/164040DF33640568
283. Rebecchi C. Yale to become first university to offer the Beyond Burger at its on-campus dining halls. Business Wire [Internet]. 2017 April 26 [cited 2024 Nov 19]. https://infoweb.newsbank.com/apps/news/document-view?p=WORLDNEWS&docref=news/164040DF33640568
284. Yale Hospitality. Sustainability. (n.d.). https://hospitality.yale.edu/eat-well/sourcing-sustainability/sustainability [accessed June 25, 2025]
285. Buzalka M. Innovator of the Month: Central Washington University Dining steps up to offer safe but appealing meal choices for its coronavirus-restricted customers. Food Management [Internet]. 2021 Jan 5 [cited 2024 Nov 19]. https://www.proquest.com/trade-journals/innovator-month-central-washington-university/docview/2475109652/se-2
286. Gingerella B. Setting the trend. FoodService Director [Internet]. 2017 July [cited 2024 Nov 19]. (30)7: 82-83. https://www.proquest.com/trade-journals/setting-trend/docview/1930104360/se-2?accountid=14826
287. The Menus of Change University Research Collaborative. The Protein Flip. (n.d.). https://static1.squarespace.com/static/5e73dab948933135a78e16ae/t/67b75a15d3387f23e42e4619/1740069398736/MCURC-Protein-Flip-Strategy.pdf
288. UC Berkeley Dining. Sustainable Food. (n.d.). https://dining.berkeley.edu/sustainability/sustainable-food/ [accessed June 26, 2025]
289. Steele A. Rustic cooking from Japan Campus dining involves DIY cooking stations DIY campus dining cooking stations and mix-and-match ingredients make for healthful variety. The Philadelphia Inquirer (PA) [Internet]. 2017 Oct 5 [cited 2024 Nov 19]. https://infoweb.newsbank.com/apps/news/document-view?p=WORLDNEWS&docref=news/1675BDE1D3269680

290. Florida State University. World Water Day at FSU Poured Down the Facts. (2018). https://sustainablecampus.fsu.edu/about/news/article/world-water-day-2018 [accessed June 26, 2025]
291. How to eat vegetarian on campus. FSView & Florida Flambeau: Florida State University (Tallahassee, FL) [Internet]. 2018 April 16 [cited 2024 Nov 19]. https://infoweb.newsbank.com/apps/news/document-view?p=WORLDNEWS&docref=news/16B54172F1836A40
292. Buzalka M. 5 things: Excess produce goes to feed exotic animals at Texas A&M. Food Management [Internet]. 2022 March 1 [cited 2024 Nov 19]. https://www.proquest.com/trade-journals/5-things-excess-produce-goes-feed-exotic-animals/docview/2634589107/se-2
293. Mendoza A. Jubilee Co-op Cafe serves free vegan meals. Daily Trojan. 2022 March 1. 205(29): 1.
294. Yeung N. Your Guide to Freebies at USC — Food Edition. (n.d.). https://www.trojans360.com/trojans360posts/your-guide-to-freebies-at-usc-food-edition?rq=Jubilee%20Vegan%20Caf%C3%A9 [accessed November 21, 2024]
295. Binghamton University Dining Services. BUDS Residential Guide to Plant-Forward Dining. (2024). https://media-prd.sodexomyway.net/web/en-us/media/Binghamton_2024_Plant-ForwardResidentDiningGuide_Trifold_MDC_tcm17-46690.pdf [accessed July 1, 2025]
296. Association for the Advancement of Sustainability in Higher Education. Colgate University OP-8: Sustainable Dining. (n.d.). https://reports.aashe.org/institutions/colgate-university-ny/report/2022-10-11/OP/food-dining/OP-8/ [accessed August 11, 2025]
297. Association for the Advancement of Sustainability in Higher Education. Florida State University OP-8: Sustainable Dining. (n.d.). https://reports.aashe.org/institutions/florida-state-university-fl/report/2023-03-15/OP/food-dining/OP-8/ [accessed August 8, 2025]
298. Association for the Advancement of Sustainability in Higher Education. Indiana University Bloomington OP-7: Food and Beverage Purchasing. (n.d.). https://reports.aashe.org/institutions/indiana-university-bloomington-in/report/2024-01-18/OP/food-dining/OP-7/ [accessed August 11, 2025]
299. Aramark Joins Client Partners to Present at Advancement of Sustainability in Higher Education (AASHE) Conference - Colleges and Universities to share campus sustainability best practices. PR Newswire (USA) [Internet]. 2011 Oct 4 [cited 2024 Nov 19]. https://infoweb.newsbank.com/apps/news/document-view?p=WORLDNEWS&docref=news/13A2C4184CC40548
300. Association for the Advancement of Sustainability in Higher Education. Muhlenberg College OP-8: Sustainable Dining. (n.d.). https://reports.aashe.org/institutions/muhlenberg-college-pa/report/2024-02-27/OP/food-dining/OP-8/ [accessed August 8, 2025]
301. U of F rated in top ten of best colleges in country for vegetarians. West Palm Beach Examiner (FL). 2011 Dec 28.
302. Northern Arizona University. Plant Based Dining on Campus. (n.d.). https://in.nau.edu/wp-content/uploads/sites/200/2022-Temp-updates-Plant-Based-Dining-Guide-1.pdf [accessed July 8, 2025]
303. Association for the Advancement of Sustainability in Higher Education. Salisbury University OP-8: Sustainable Dining. (n.d.). https://reports.aashe.org/institutions/salisbury-university-md/report/2023-10-30/OP/food-dining/OP-8/ [accessed August 11, 2025]

304. Buzalka M. CulinArt creates plant-based dining guide at Stony Brook University. FoodService Director [Internet]. 2022 Nov 8 [cited 2025 June 30]. https://www.foodservicedirector.com/colleges-universities/culinart-creates-plant-based-dining-guide-at-stony-brook-university
305. Association for the Advancement of Sustainability in Higher Education. University of New Hampshire OP-8: Sustainable Dining. (n.d.). https://reports.aashe.org/institutions/university-of-new-hampshire-nh/report/2024-10-24/OP/food-dining/OP-8/ [accessed August 8, 2025]
306. Gosling N. From the Dining Halls to the Farm Fields: A brief look at UNH's composting program. UNH Today. 2023 June 15.
307. University of New Hampshire. The Wildcat Plate. (n.d.). https://www.unh.edu/dining/nutrition/wildcat-plate [accessed June 30, 2025]
308. de Sousa A. The Invisible Power of 'Nudging' Is Leading Diners to Cut Back on Meat. Bloomberg.com [Internet]. 2024 April 30 [cited 2024 Nov 19]. https://search.ebscohost.com/login.aspx?direct=true&db=bth&AN=176932273&scope=site
309. Gottlieb M, Roazzi M. Peta2 flunks Binghamton University's vegan options. Pipe Dream (Binghamton University) (NY) [Internet]. 2013 Oct 15 [cited 2024 Nov 19]. https://infoweb.newsbank.com/apps/news/document-view?p=WORLDNEWS&docref=news/14988502D7118868
310. Brandeis Hospitality. Menu Information. (n.d.). https://www.brandeishospitality.com/health-wellbeing/menu-information/ [accessed June 16, 2025]
311. Bryn Mawr College. Nutrition. (n.d.). https://www.brynmawr.edu/inside/offices-services/dining-services/nutrition [accessed August 6, 2025]
312. Central Washington University. Campus Dining. (n.d.). https://www.cwu.edu/student-life/dining/nutrition-allergies.php#accordion-b5793686-7b95-487d-8b3b-2005c662dbdd-0 [accessed July 2, 2025]
313. College of Charleston. Plant Based and Vegan Options. (n.d.) https://charleston.campusdish.com/Sustainability/PlantBasedandVegan [accessed on July 2, 2025]
314. Colorado State University. Vegan & Vegetarian Plant-Based Diet Options. (n.d.). https://eatwell.colostate.edu/veganvegetarian-options/ [accessed on July 8, 2025]
315. Columbia University. Food Allergies & Special Diets. (n.d.). https://dining.columbia.edu/content/food-allergies-special-diets [accessed on July 2, 2025]
316. Framingham State University Dining Services. Nutrition. (n.d.). https://framingham.sodexomyway.com/en-us/explore/nutrition [accessed on July 8, 2025]
317. Rojas V. Vegan-friendly campus a goal for ISU students. Indiana Statesman: Indiana State University (Terre Haute, IN) [Internet]. 2014 Nov 17 [cited 2024 Nov 19]. https://infoweb.newsbank.com/apps/news/document-view?p=WORLDNEWS&docref=news/152E609051A45F68

318. Association for the Advancement of Sustainability in Higher Education. Indiana University Bloomington OP-8: Sustainable Dining. (n.d.). https://reports.aashe.org/institutions/indiana-university-bloomington-in/report/2024-01-18/OP/food-dining/OP-8/ [accessed August 8, 2025]
319. Indiana University. Diet and Preferences. (n.d.). https://dining.indiana.edu/nutrition/diet-and-preferences/index.html [accessed July 3, 2025]
320. Johns Hopkins University. Allergens & Dietary Preferences. (n.d.). https://studentaffairs.jhu.edu/dining/wp-content/uploads/sites/72/2023/06/Allergens-and-Dietary-Preference-Key-updated-2024.png [accessed on July 2, 2025]
321. Killett G. Loyola's vegans and vegetarians want more on-campus options. The Maroon: Loyola University - New Orleans (LA) [Internet]. 2021 Oct 18 [cited 2024 Nov 19]. https://infoweb.newsbank.com/apps/news/document-view?p=WORLDNEWS&docref=news/185B9050FA75D9D0
322. From Tikka Masala to Congee, Campus Dining Trends Go Beyond Chicken Tenders & Pizza - Sodexo, Which Runs Dining Services at More Than 600 U.S. Colleges and Universities, Names Top Dining Trends. PR Newswire (USA) [Internet]. 2018 Sept 18 [cited 2024 Nov 19]. https://infoweb.newsbank.com/apps/news/document-view?p=WORLDNEWS&docref=news/16E83A6A05C41300
323. Michigan State University. Sustainability. (n.d.). https://sustainability.msu.edu/Campus/Sustainable-Food/Sustainable-Food-and-Security [accessed July 2, 2025]
324. Everything's Kosher at New Muhlenberg Facility. Foodservice Director [Internet]. 2010 Oct 15 [cited 2024 Nov 19]. 23(10): 6-6,8. https://www.proquest.com/trade-journals/everythings-kosher-at-new-muhlenberg-facility/docview/759551675/se-2
325. NC State Dining. Special Dietary Needs. (n.d.). https://dining.ncsu.edu/nutrition/special-dietary-needs/ [accessed July 2, 2025]
326. Northeastern University Dining. Our Sustainability Commitment. (n.d.). https://new.dineoncampus.com/public/our-sustainability-commitment [accessed July 2, 2025]
327. Enzweiler P. NKU's healthy food policy unique among Kentucky universities. Northerner, The: Northern Kentucky University (Highland Heights, KY) [Internet]. 2015 Feb 6 [cited 2024 Nov 19]. https://infoweb.newsbank.com/apps/news/document-view?p=WORLDNEWS&docref=news/16223F869D6D18A8
328. Oregon State University. Food Labeling. (n.d.). https://uhds.oregonstate.edu/dining/nutrition/food-labeling [accessed July 2, 2025]
329. Allergen Friendly Skidmore. (n.d.). https://www.skidmore.edu/diningservice/documents/AllergenFriendlySkidmore.pdf [accessed July 2, 2025]
330. Skidmore eat healthy strives to improve wellness. Foodservice Director [Internet]. 2012 Jan 15 [cited 2024 Nov 19]. 25(1): 10. https://www.proquest.com/trade-journals/skidmore-eat-healthy-strives-improve-wellness/docview/1001343555/se-2
331. Syracuse University Maxwell School of Citizenship & Public Affairs. Meatless Monday. (n.d.). https://www.maxwell.syr.edu/research/lerner-center/healthy-monday/meatless-monday [accessed June 5, 2025]

332. Association for the Advancement of Sustainability in Higher Education. University of Iowa OP-8: Sustainable Dining. (n.d.). https://reports.aashe.org/institutions/university-of-iowa-ia/report/2024-06-25/OP/food-dining/OP-8/ [accessed August 8, 2025]
333. University at Buffalo. Carbon Footprint. (n.d.). https://ubdining.com/sustainability/carbon-footprint [accessed July 3, 2025]
334. University at Buffalo. Climate Action Plan. (n.d.). https://www.buffalo.edu/climate-action/10-solutions/solution-8.html#:~:text=Fall%202022%20%2D%20Spring%202023,can%20have%20a%20large%20impact [accessed July 3, 2025]
335. University at Buffalo. Nutrition Icons. (n.d.) https://ubdining.com/nutrition/nutrition-icons [accessed July 3, 2025]
336. Fitzpatrick T. Thinking Outside the Bread Box. Food Management [Internet]. 2010 May [cited 2024 Nov 19]. 45(5): 36-40. https://www.proquest.com/trade-journals/thinking-outside-bread-box/docview/215903346/se-2?accountid=14826
337. UC Berkeley Dining. Carbon Footprint. (n.d.). https://dining.berkeley.edu/sustainability/carbon-footprint/ [accessed July 3, 2025]
338. Franchini C, Bartolotto C, Scazzina F, Carpenter CL, Slusser W. Increasing the consumption of environmentally friendly foods in a University dining hall using menu item placement. Nutrients (2023) 15:18, 3873. doi: 10.3390/nu15183873
339. The Veg Heads: On-campus vegan food. Daily Bruin: University of California-Los Angeles (CA) [Internet]. 2013 Nov 12 [cited 2024 Nov 19]. https://infoweb.newsbank.com/apps/news/document-view?p=WORLDNEWS&docref=news/14A0889E04031980
340. UCLA Dining. Fight Climate Change with Food. (n.d.). https://dining.ucla.edu/carbonfootprint/ [accessed July 2, 2025]
341. Boss D. Elevating the Dining Experience at the University of California San Diego: FE&S. Foodservice Equipment & Supplies [Internet]. 2017 June [cited 2024 Nov 19]. 70(6): 46-50. https://www.proquest.com/trade-journals/elevating-dining-experience-at-university/docview/1963838313/se-2 (accessed November 19, 2024)
342. Association for the Advancement of Sustainability in Higher Education. University of Central Florida OP-8: Sustainable Dining. (n.d.). https://reports.aashe.org/institutions/university-of-central-florida-fl/report/2024-04-23/OP/food-dining/OP-8/ [accessed August 8, 2025]
343. University of Colorado Boulder. Dietary Accommodations. (n.d.). https://www.colorado.edu/living/dining/dietary-accommodations [accessed July 3, 2025]
344. Association for the Advancement of Sustainability in Higher Education. University of Colorado Colorado Springs OP-8: Sustainable Dining. (n.d.). https://reports.aashe.org/institutions/university-of-colorado-at-colorado-springs-co/report/2024-03-01/OP/food-dining/OP-8/ [accessed August 8, 2025]
345. University of Maine at Presque Isle. Nutrition. (n.d.). https://presqueisle.sodexomyway.com/en-us/explore/nutrition [accessed July 3, 2025]
346. Association for the Advancement of Sustainability in Higher Education. University of Massachusetts Amherst OP-8: Sustainable Dining. (n.d.). https://reports.aashe.org/institutions/university-of-massachusetts-amherst-ma/report/2023-02-17/OP/food-dining/OP-8/ [accessed August 11, 2025]
347. UMass Amherst. UMass Dining Sustainability Initiatives. (n.d.). https://umassdining.com/sustainability [accessed July 3, 2025]

348. Association for the Advancement of Sustainability in Higher Education. University of Miami OP-8: Sustainable Dining. (n.d.). https://reports.aashe.org/institutions/university-of-miami-fl/report/2025-03-28/OP/food-dining/OP-8/ [accessed August 11, 2025]
349. Association for the Advancement of Sustainability in Higher Education. University of Michigan OP-8: Sustainable Dining. (n.d.). https://reports.aashe.org/institutions/university-of-michigan-mi/report/2022-12-19/OP/food-dining/OP-8/ [accessed August 8, 2025]
350. Michigan Dining. Carbon Impact and MHealthy Icons. (n.d.). https://dining.umich.edu/menus-locations/introducing-new-carbon-and-mhealthy-icons/ [accessed July 3, 2025]
351. Michigan Dining. Sustainable Eating in Dining Halls. (n.d.). https://www.canva.com/design/DAFtm2zCY5w/pu1KgVlWfCrvTazO2oVq1A/view?utm_content=DAFtm2zCY5w&utm_campaign=designshare&utm_medium=link&utm_source=editor#1 [accessed July 3, 2025]
352. Association for the Advancement of Sustainability in Higher Education. University of Minnesota, Duluth OP-8: Sustainable Dining. (n.d.). https://reports.aashe.org/institutions/university-of-minnesota-duluth-mn/report/2024-09-11/OP/food-dining/OP-8/ [accessed August 8, 2025]
353. University of Notre Dame. Nutrition, Allergies & Health. (n.d.). https://dining.nd.edu/nutrition-health/ [accessed July 3, 2025]
354. University of Notre Dame. Sustainability. (n.d.). https://dining.nd.edu/about/values/sustainability/ [accessed July 3, 2025]
355. App-etizing Dining Programs. Food Management [Internet]. 2012 March [cited 2024 Nov 19]. 47(3): 8. https://www.proquest.com/trade-journals/app-etizing-dining-programs/docview/926582583/se-2
356. University of Oregon. UO Sustainability Dashboard. (n.d.). https://pages.uoregon.edu/infographics/sustainability/food.html [accessed July 3, 2025]
357. Nguyen A. Meatless eating is hot on Philadelphia college campuses. Philadelphia Daily News (PA) [Internet]. 2010 Oct 14 [cited 2024 Nov 19]. 25. https://infoweb.newsbank.com/apps/news/document-view?p=WORLDNEWS&docref=news/132DB2CDCA38B6E8
358. University of San Diego. Wellness. (n.d.). https://www.sandiego.edu/dining/wellness.php [accessed July 3, 2025]
359. Association for the Advancement of Sustainability in Higher Education. University of Vermont OP-8: Sustainable Dining. (n.d.). https://reports.aashe.org/institutions/university-of-vermont-vt/report/2023-02-06/OP/food-dining/OP-8/ [accessed August 8, 2025]
360. University of Vermont. Nutrition. (n.d.). https://uvmdining.sodexomyway.com/en-us/explore/nutrition [accessed July 3, 2025]
361. Washington State University. Dietary Needs & Allergens. (n.d.). https://dining.wsu.edu/nutrition/ [accessed July 3, 2025]
362. Alma College. Alma College partners with Metz Culinary Management for campus dining services. The Morning Sun (Mount Pleasant - Alma, MI) [Internet]. 2022 June 24 [cited 2024 Nov 19]. https://infoweb.newsbank.com/apps/news/document-view?p=WORLDNEWS&docref=news/18ADAF169E3AC8E8

363. Buzalka M. C&U Innovator of the Year: Northern Arizona University/Sodexo. Food Management [Internet]. 2018 Jan 27 [cited 2024 Nov 19]. https://www.proquest.com/trade-journals/c-amp-u-innovator-year-northern-arizona/docview/1991578752/se-2?accountid=14826
