# Supplementary material for: Higher education institutions and the use of marketing-mix choice architecture strategies to encourage plant-rich menu options and sustainable dietary patterns: a scoping review
Source: Front Nutr. 2026 Mar 24;13:1774451. doi: 10.3389/fnut.2026.1774451 (PMC13053224; doi:10.3389/fnut.2026.1774451)
Supplement: Supplementary file 4 [file Supplementary_File_4.pdf]

Supplemental File 4: List of identified higher education institutions, region, state, institutional size, campus setting, and MMCA strategies applied

Higher Education Institution	Region	State	Institutional Size	Campus Setting	MMCA Strategies
Alma College	Midwest	Alma, Michigan	Small	Highly residential	Profile, Proximity
American University	South	Washington, D.C.	Medium	Highly residential	Profile, Promotion
Antioch College	Midwest	Yellow Springs, Ohio	Very Small	Highly residential	Profile
Appalachian State University	South	Boone, North Carolina	Large	Primarily residential	Profile
Arizona State University	West	Tempe, Arizona	Very Large	Residential	Profile, Priming/Prompting
Bastyr University	West	Kenmore, Washington	Small	Graduate-focused	Profile
Belmont University	South	Nashville, Tennessee	Medium	Highly residential	Profile
Benedict College	South	Columbia, South Carolina	Small	Highly residential	Profile
Bennington College	Northeast	Bennington, Vermont	Small	Highly residential	Profile
Bentley University	Northeast	Waltham, Massachusetts	Medium	Highly residential	Profile, Priming/Prompting
Berry College	South	Mount Berry, Georgia	Small	Highly residential	Profile
Binghamton University	Northeast	Vestal, New York	Large	Primarily residential	Profile, Promotion, Priming/Prompting
Boston College	Northeast	Chestnut Hill, Massachusetts	Medium	Highly residential	Profile
Boston University	Northeast	Boston, Massachusetts	Very Large	Highly residential	Profile, Promotion, Priming/Prompting
Bowdoin College	Northeast	Brunswick, Maine	Small	Highly residential	Profile, Priming/Prompting
Bowling Green State University	Midwest	Bowling Green, Ohio	Large	Primarily residential	Profile
Brandeis University	Northeast	Waltham, Massachusetts	Medium	Highly residential	Profile, Priming/Prompting
Brigham Young University	West	Provo, Utah	Very Large	Residential	Profile, Promotion, Picks, Priming/Prompting
Bryn Mawr College	Northeast	Bryn Mawr, Pennsylvania	Small	Highly residential	Profile, Priming/Prompting
Bucknell University	Northeast	Lewisburg, Pennsylvania	Small	Highly residential	Profile, Promotion

California Polytechnic State University (Cal Poly)	West	San Luis Obispo, California	Large	Primarily residential	Profile
California State University, Chico (Chico State)	West	Chico, California	Medium	Residential	Profile
Canisius University (formerly Canisius College)	Northeast	Buffalo, New York	Small	Highly residential	Profile, Pricing
Carnegie Mellon University	Northeast	Pittsburgh, Pennsylvania	Medium	Highly residential	Profile, Priming/Prompting
Central Washington University	West	Ellensburg, Washington	Medium	Residential	Profile, Portion, Promotion, Priming/Prompting
Clark University	Northeast	Worcester, Massachusetts	Small	Highly residential	Profile, Promotion
Colby College	Northeast	Waterville, Maine	Small	Highly residential	Profile
Colgate University	Northeast	Hamilton, New York	Small	Highly residential	Profile, Promotion
College of Charleston	South	Charleston, South Carolina	Medium	Primarily residential	Profile, Promotion, Priming/Prompting
College of the Atlantic	Northeast	Bar Harbor, Maine	Very Small	Highly residential	Profile
College of the Holy Cross	Northeast	Worcester, Massachusetts	Small	Highly residential	Profile, Promotion
Colorado Mountain College	West	Glenwood Springs, Colorado	Medium	Primarily non-residential	Profile
Colorado State University	West	Fort Collins, Colorado	Large	Primarily residential	Profile, Promotion, Picks, Priming/Prompting
Columbia University in the City of New York	Northeast	New York, New York	Large	Highly residential	Profile, Portion, Picks, Priming/Prompting
Cornell University	Northeast	Ithaca, New York	Large	Highly residential	Profile, Portion
Davidson College	South	Davidson, North Carolina	Small	Highly residential	Profile
DePaul University	Midwest	Chicago, Illinois	Large	Residential	Profile

Drexel University	Northeast	Philadelphia, Pennsylvania	Large	Residential	Profile, Priming/Prompting
Duke University	South	Durham, North Carolina	Medium	Highly residential	Profile, Promotion
Endicott College	Northeast	Beverly, Massachusetts	Medium	Highly residential	Profile
Fairfield University	Northeast	Fairfield, Connecticut	Medium	Highly residential	Profile
Florida Agricultural and Mechanical University	South	Tallahassee, Florida	Medium	Primarily residential	Profile
Florida Institute of Technology (Florida Tech)	South	Melbourne, Florida	Medium	Highly residential	Profile
Florida State University	South	Tallahassee, Florida	Very Large	Residential	Profile, Pricing, Promotion, Priming/Prompting
Framingham State University	Northeast	Framingham, Massachusetts	Medium	Highly residential	Profile, Priming/Prompting
Franklin & Marshall College	Northeast	Lancaster, Pennsylvania	Small	Highly residential	Profile
Georgetown College	South	Georgetown, Kentucky	Small	Highly residential	Profile
Georgia Institute of Technology (Georgia Tech)	South	Atlanta, Georgia	Very Large	Primarily Residential	Profile, Priming/Prompting
Georgia Southern University	South	Statesboro, Georgia	Large	Residential	Profile
Georgia State University	South	Atlanta, Georgia	Very Large	Residential	Profile, Promotion
Hamilton College	Northeast	Clinton, New York	Small	Highly residential	Profile
Harvard University	Northeast	Cambridge, Massachusetts	Large	Highly residential	Profile, Picks
Indiana State University	Midwest	Terre Haute, Indiana	Medium	Residential	Priming/Prompting
Indiana University Bloomington	Midwest	Bloomington, Indiana	Very Large	Primarily residential	Profile, Promotion, Priming/Prompting
Ithaca College	Northeast	Ithaca, New York	Medium	Highly residential	Profile, Promotion, Proximity

John Jay College of Criminal Justice (CUNY John Jay College of Criminal Justice)	Northeast	New York, New York	Medium	Mostly full-time, non-residential	Profile
Johns Hopkins University	South	Baltimore, Maryland	Large	Primarily residential	Profile, Promotion, Priming/Prompting
Johnson & Wales University (North Miami campus)*	South	North Miami, Florida	N/A	N/A	Profile, Portion
Kent State University	Midwest	Kent, Ohio	Large	Primarily residential	Profile
Lee University	South	Cleveland, Tennessee	Medium	Highly residential	Profile
Lehigh University	Northeast	Bethlehem, Pennsylvania	Medium	Highly residential	Profile
Liberty University	South	Lynchburg, Virginia	Very Large	Online and on-campus learning	Profile
Loyola Marymount University	West	Los Angeles, California	Medium	Primarily residential	Profile
Loyola University New Orleans	South	New Orleans, Louisiana	Medium	Primarily residential	Priming/Prompting
Madonna University	Midwest	Livonia, Michigan	Small	Residential	Profile
Maharishi International University (formerly Maharishi University of Management)	Midwest	Fairfield, Iowa	Small	Online and on-campus learning	Profile
Marist University (formerly Marist College)	Northeast	Poughkeepsie, New York	Medium	Highly residential	Profile
Marquette University	Midwest	Milwaukee, Wisconsin	Medium	Highly residential	Priming/Prompting
Michigan State University	Midwest	East Lansing, Michigan	Very Large	Primarily residential	Profile, Portion, Priming/Prompting

Minnesota State University, Mankato	Midwest	Mankato, Minnesota	Medium	Residential	Profile
Montclair State University	Northeast	Montclair, New Jersey	Large	Primarily residential	Profile, Promotion
Moravian University	Northeast	Bethlehem, Pennsylvania	Small	Highly residential	Profile
Muhlenberg College	Northeast	Allentown, Pennsylvania	Small	Highly residential	Promotion, Priming/Prompting
Nazareth University (formerly Nazareth College)	Northeast	Rochester, New York	Small	Highly residential	Profile
New York University	Northeast	New York, New York	Very Large	Primarily residential	Profile, Promotion, Priming/Prompting, Proximity
North Carolina State University	South	Raleigh, North Carolina	Very Large	Primarily residential	Profile, Promotion, Picks, Priming/Prompting
Northeastern University	Northeast	Boston, Massachusetts	Large	Highly residential	Profile, Portion, Priming/Prompting
Northern Arizona University	West	Flagstaff, Arizona	Large	Primarily residential	Profile, Promotion, Priming/Prompting, Proximity
Northern Kentucky University	South	Highland Heights, Kentucky	Medium	Residential	Priming/Prompting
Northern Michigan University	Midwest	Marquette, Michigan	Medium	Primarily residential	Profile, Priming/Prompting
Northwestern University	Midwest	Evanston, Illinois	Large	Primarily residential	Profile, Promotion
Ohio University	Midwest	Athens, Ohio	Large	Residential	Profile, Promotion
Oklahoma City University	South	Oklahoma City, Oklahoma	Small	Highly residential	Profile
Oregon State University	West	Corvallis, Oregon	Large	Residential	Profile, Portion, Promotion, Picks, Priming/Prompting
Providence College	Northeast	Providence, Rhode Island	Medium	Highly residential	Profile
Quinnipiac University	Northeast	Hamden, Connecticut	Medium	Highly residential	Profile, Promotion
Rice University	South	Houston, Texas	Medium	Highly residential	Profile, Promotion

Rider University	Northeast	Lawrenceville, New Jersey	Medium	Highly residential	Profile
Roanoke College	South	Salem, Virginia	Small	Highly residential	Profile
Rochester Institute of Technology	Northeast	Rochester, New York	Medium	Highly residential	Profile
Rollins College	South	Winter Park, Florida	Small	Highly residential	Profile
Rutgers, The State University of New Jersey (Rutgers University)	Northeast	New Brunswick, New Jersey	Very Large	Primarily residential	Profile, Promotion
Salisbury University	South	Salisbury, Maryland	Medium	Primarily residential	Profile, Promotion, Priming/Prompting
San Diego State University	West	San Diego, California	Large	Residential	Profile, Promotion, Priming/Prompting
Seattle Pacific University	West	Seattle, Washington	Small	Highly residential	Profile
Seattle University	West	Seattle, Washington	Medium	Highly residential	Profile, Portion, Priming/Prompting, Proximity
Skidmore College	Northeast	Saratoga Springs, New York	Small	Highly residential	Profile, Priming/Prompting
Smith College	Northeast	Northampton, Massachusetts	Small	Highly residential	Profile
Southern University and Agricultural & Mechanical College	South	Baton Rouge, Louisiana	Medium	Highly residential	Profile
St. John Fisher University (formerly St. John Fisher College)	Northeast	Rochester, New York	Small	Highly residential	Profile
Stanford University	West	Stanford, California	Large	Highly residential	Place, Profile, Portion, Promotion, Picks, Priming/Prompting, Proximity
Stony Brook University	Northeast	Stony Brook, New York	Large	Highly residential	Profile, Promotion, Priming/Prompting

Syracuse University	Northeast	Syracuse, New York	Large	Highly residential	Profile, Promotion, Priming/Prompting
The Ohio State University	Midwest	Columbus, Ohio	Very Large	Primarily residential	Profile, Pricing, Promotion, Picks, Priming/Prompting
The Pennsylvania State University (Penn State)	Northeast	University Park, Pennsylvania	Very Large	Primarily residential	Profile, Portion, Promotion, Priming/Prompting
The University of Arizona	West	Tucson, Arizona	Very Large	Residential	Profile, Portion, Pricing, Promotion, Picks
The University of Iowa	Midwest	Iowa City, Iowa	Large	Primarily residential	Profile, Promotion, Priming/Prompting
The University of Oklahoma	South	Norman, Oklahoma	Large	Primarily residential	Profile, Promotion
The University of Texas at Austin	South	Austin, Texas	Very Large	Residential	Profile, Portion, Promotion
The University of Utah	West	Salt Lake City, Utah	Large	Residential	Profile, Priming/Prompting
Towson University	South	Towson, Maryland	Large	Primarily residential	Profile, Promotion
Tulane University of Louisiana	South	New Orleans, Louisiana	Medium	Primarily residential	Profile
University at Albany	Northeast	Albany, New York	Medium	Highly residential	Profile, Promotion, Priming/Prompting
University at Buffalo	Northeast	Buffalo, New York	Large	Primarily residential	Profile, Promotion, Priming/Prompting
University of California, Berkeley	West	Berkeley, California	Very Large	Primarily residential	Profile, Portion, Promotion, Picks, Priming/Prompting
University of California, Davis	West	Davis, California	Very Large	Primarily residential	Profile, Portion, Promotion, Picks, Priming/Prompting, Proximity
University of California, Irvine	West	Irvine, California	Large	Highly residential	Place, Profile, Pricing, Priming/Prompting
University of California, Los Angeles	West	Los Angeles, California	Very Large	Highly residential	Profile, Portion, Promotion, Priming/Prompting

University of California, Riverside	West	Riverside, California	Large	Primarily residential	Profile, Portion, Promotion
University of California, San Deigo	West	La Jolla, California	Very Large	Highly residential	Profile, Portion, Pricing, Promotion, Picks, Priming/Prompting
University of California, Santa Barbara	West	Santa Barbara, California	Large	Primarily residential	Profile, Promotion
University of California, Santa Cruz	West	Santa Cruz, California	Large	Primarily residential	Profile, Promotion
University of Central Florida	South	Orlando, Florida	Very Large	Residential	Profile, Priming/Prompting
University of Colorado Boulder	West	Boulder, Colorado	Very Large	Residential	Profile, Portion, Promotion, Picks, Priming/Prompting
University of Colorado Colorado Springs	West	Colorado Springs, Colorado	Medium	Residential	Profile, Priming/Prompting
University of Connecticut	Northeast	Storrs, Connecticut	Large	Highly residential	Profile
University of Dayton	Midwest	Dayton, Ohio	Medium	Highly residential	Profile
University of Florida	South	Gainesville, Florida	Very Large	Primarily residential	Profile
University of Georgia	South	Athens, Georgia	Very Large	Primarily residential	Profile, Portion, Promotion, Picks
University of Hawaii at Mānoa	West	Honolulu, Hawaii	Large	Residential	Profile, Priming/Prompting
University of Illinois at Urbana-Champaign	Midwest	Champaign, Illinois	Very Large	Primarily residential	Profile, Priming/Prompting
University of Maine at Presque Isle	Northeast	Presque Isle, Maine	Small	Online and on-campus learning	Profile, Priming/Prompting
University of Maryland, College Park	South	College Park, Maryland	Very Large	Primarily residential	Profile, Portion, Promotion, Priming/Prompting
University of Massachusetts Amherst	Northeast	Amherst, Massachusetts	Large	Highly residential	Profile, Promotion, Priming/Prompting
University of Miami	South	Coral Gables, Florida	Large	Primarily residential	Profile, Priming/Prompting

University of Michigan-Ann Arbor	Midwest	Ann Arbor, Michigan	Very Large	Primarily residential	Profile, Promotion, Picks, Priming/Prompting
University of Minnesota Duluth	Midwest	Duluth, Minnesota	Medium	Primarily residential	Profile, Priming/Prompting
University of Nebraska-Lincoln	Midwest	Lincoln, Nebraska	Large	Primarily residential	Profile, Promotion
University of New Hampshire	Northeast	Durham, New Hampshire	Medium	Highly residential	Profile, Portion, Promotion, Priming/Prompting
University of North Carolina at Chapel Hill	South	Chapel Hill, North Carolina	Large	Primarily residential	Profile
University of North Texas	South	Denton, Texas	Very Large	Residential	Profile, Portion, Promotion
University of Notre Dame	Midwest	Notre Dame, Indiana	Medium	Highly residential	Profile, Priming/Prompting
University of Oregon	West	Eugene, Oregon	Large	Primarily residential	Profile, Priming/Prompting
University of Pennsylvania	Northeast	Philadelphia, Pennsylvania	Large	Highly residential	Profile, Priming/Prompting
University of Pittsburgh	Northeast	Pittsburgh, Pennsylvania	Large	Primarily residential	Profile, Priming/Prompting
University of Portland	West	Portland, Oregon	Medium	Highly residential	Profile
University of Rochester	Northeast	Rochester, New York	Medium	Highly residential	Profile, Promotion
University of San Diego	West	San Diego, California	Medium	Primarily residential	Profile, Priming/Prompting
University of South Carolina	South	Columbia, South Carolina	Large	Primarily residential	Profile
University of South Florida	South	Tampa, Florida	Very Large	Residential	Profile, Pricing
University of Southern California	West	Los Angeles, California	Very Large	Primarily residential	Profile, Portion, Pricing, Promotion, Picks, Priming/Prompting
University of Vermont	Northeast	Burlington, Vermont	Medium	Highly residential	Profile, Priming/Prompting
University of Virginia	South	Charlottesville, Virginia	Large	Primarily residential	Profile, Promotion, Priming/Prompting
University of Washington	West	Seattle, Washington	Very Large	Primarily residential	Profile, Promotion, Priming/Prompting

University of Wisconsin-Madison	Midwest	Madison, Wisconsin	Very Large	Primarily residential	Profile, Promotion
University of Wisconsin-Oshkosh	Midwest	Oshkosh, Wisconsin	Medium	Primarily residential	Profile, Promotion
University of Wyoming	West	Laramie, Wyoming	Medium	Residential	Profile, Proximity
Vanderbilt University	South	Nashville, Tennessee	Medium	Highly residential	Profile, Portion, Priming/Prompting
Villanova University	Northeast	Villanova, Pennsylvania	Medium	Highly residential	Profile, Portion, Priming/Prompting
Virginia Polytechnic Institute and State University (Virginia Tech)	South	Blacksburg, Virginia	Large	Primarily residential	Profile, Promotion
Wake Forest University	South	Winston-Salem, North Carolina	Medium	Highly residential	Profile, Portion, Priming/Prompting, Proximity
Washington State University	West	Pullman, Washington	Large	Primarily residential	Profile, Pricing, Promotion, Picks, Priming/Prompting, Proximity
Wellesley College	Northeast	Wellesley, Massachusetts	Small	Highly residential	Profile
Western Oregon University	West	Monmouth, Oregon	Medium	Primarily residential	Profile
Williams College	Northeast	Williamstown, Massachusetts	Small	Highly residential	Profile
Yale University	Northeast	New Haven, Connecticut	Medium	Highly residential	Profile, Promotion, Priming/Prompting
